# Lysosomal Disorders Drive Susceptibility to Tuberculosis by Compromising Macrophage Migration

**DOI:** 10.1016/j.cell.2016.02.034

**Published:** 2016-03-24

**Authors:** Russell D. Berg, Steven Levitte, Mary P. O’Sullivan, Seónadh M. O’Leary, C.J. Cambier, James Cameron, Kevin K. Takaki, Cecilia B. Moens, David M. Tobin, Joseph Keane, Lalita Ramakrishnan

**Affiliations:** 1 Molecular & Cellular Biology Graduate Program and Medical Scientist Training Program, University of Washington, Seattle, WA 98195, USA; 2 Department of Medicine, University of Cambridge, MRC Laboratory of Molecular Biology, Cambridge CB2 0QH, UK; 3 Department of Clinical Medicine, Institute of Molecular Medicine, Trinity College Dublin, Dublin 8, Ireland; 4 Immunology Graduate Program, University of Washington, Seattle, WA 98195, USA; 5 Department of Microbiology, University of Washington, Seattle, WA 98195, USA; 6 Fred Hutchinson Cancer Research Center, Seattle, WA 98109, USA; 7 Department of Molecular Genetics and Microbiology, Duke University, Durham, NC 27710, USA; 8 Department of Immunology, Duke University, Durham, NC 27710, USA

## Abstract

A zebrafish genetic screen for determinants of susceptibility to *Mycobacterium marinum* identified a hypersusceptible mutant deficient in lysosomal cysteine cathepsins that manifests hallmarks of human lysosomal storage diseases. Under homeostatic conditions, mutant macrophages accumulate undigested lysosomal material, which disrupts endocytic recycling and impairs their migration to, and thus engulfment of, dying cells. This causes a buildup of unengulfed cell debris. During mycobacterial infection, macrophages with lysosomal storage cannot migrate toward infected macrophages undergoing apoptosis in the tuberculous granuloma. The unengulfed apoptotic macrophages undergo secondary necrosis, causing granuloma breakdown and increased mycobacterial growth. Macrophage lysosomal storage similarly impairs migration to newly infecting mycobacteria. This phenotype is recapitulated in human smokers, who are at increased risk for tuberculosis. A majority of their alveolar macrophages exhibit lysosomal accumulations of tobacco smoke particulates and do not migrate to *Mycobacterium tuberculosis.* The incapacitation of highly microbicidal first-responding macrophages may contribute to smokers’ susceptibility to tuberculosis.

## Highlights

• Lysosomal storage diseases reduce macrophage endocytic recycling and migration
• Reduced macrophage migration increases tuberculosis severity via granuloma breakdown
• Tobacco smoke particles accumulate in lysosomes of smokers’ alveolar macrophages
• Lysosomal particles reduce smokers’ macrophage migration to infecting mycobacteria

## Graphical Abstract

**Figure:**
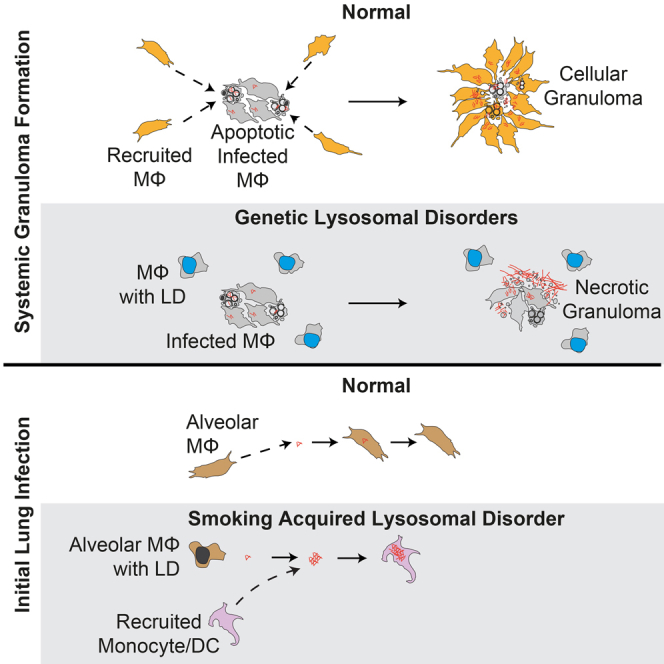

## Teaser

Lysosomal dysfunction caused by genetic mutations or accumulation of tobacco particulates in the lysosomal compartment impairs macrophage function and formation of stable tuberculous granulomas, providing a possible explanation for the increased susceptibility to tuberculosis in smokers.

## Introduction

Tuberculosis (TB) involves a series of interactions between macrophages and the infecting mycobacterium with this proposed sequence of events (Cambier et al., 2014a, Srivastava et al., 2014): inhaled mycobacteria are engulfed by lung alveolar macrophages and, if not cleared during this initial interaction, are transported deeper into the lung. Here, newly recruited myeloid and other immune cells aggregate around the infected cells to form organized granulomas.

The study of zebrafish infected with *M. marinum* has enabled the dissection of these steps of TB pathogenesis, aided by the genetic tractability of this model organism and its optical transparency during its first few weeks of life (Cambier et al., 2014a). Newly infecting bacteria can be transported across epithelial barriers by permissive macrophages (Cambier et al., 2014b). Additional macrophages are recruited to the initial infected macrophage to form the tuberculous granuloma (Cambier et al., 2014a). Cellular expansion of the granuloma, and intracellular bacterial growth within it, proceeds through apoptosis of the infected macrophages and their phagocytosis by newly arriving uninfected macrophages (Davis and Ramakrishnan, 2009). On the one hand, bacterially mediated granuloma expansion can promote infection through bacterial spread into newly recruited macrophages (Davis and Ramakrishnan, 2009). On the other hand, if the supply of uninfected macrophages is limiting, apoptotic infected cells in the granuloma undergo secondary necrosis, causing granuloma breakdown and the release of bacteria into the extracellular space, which enables their accelerated growth (Pagán et al., 2015).

In this work, we characterize a zebrafish mutant identified in a forward genetic screen (Tobin et al., 2010) to reveal how, during genetic lysosomal storage disorders, the accumulation of undegraded products in the macrophage lysosome impairs the migration of these phagocytic cells. The disruption of macrophage migration contributes to the pathogenesis of the lysosomal storage disease in the uninfected state and causes granuloma breakdown during tuberculous infection, which underlies hypersusceptibility. The mutation maps to *snapc1b*, a transcriptional co-regulator that causes lysosomal storage through reduced expression of lysosomal cysteine cathepsins B and L. Using zebrafish models of human lysosomal storage diseases, we generalize our findings to show that the accumulation of diverse biological substrates, as well as inert particles, compromises macrophage migration through the derangement of endocytic recycling. We then show that lysosomal storage in macrophages inhibits their migration to engulf newly infecting bacteria. Because the resident alveolar macrophages of human cigarette smokers have been reported to accumulate particulate material, we asked whether smokers’ macrophages are similarly compromised in their response to mycobacterial infection. We find that the majority of smokers’ alveolar macrophages have enlarged lysosomes filled with opaque material and are impaired in their migration to *M. tuberculosis*. The compromised migration of these highly microbicidal first responding macrophages suggests a mechanistic explanation for the reported association between smoking and TB (Lin et al., 2007).

## Results

### *snapc1b* Zebrafish Mutant Hypersusceptibility to *M. marinum* Infection Is Characterized by Granuloma Breakdown

**Figure 1:**
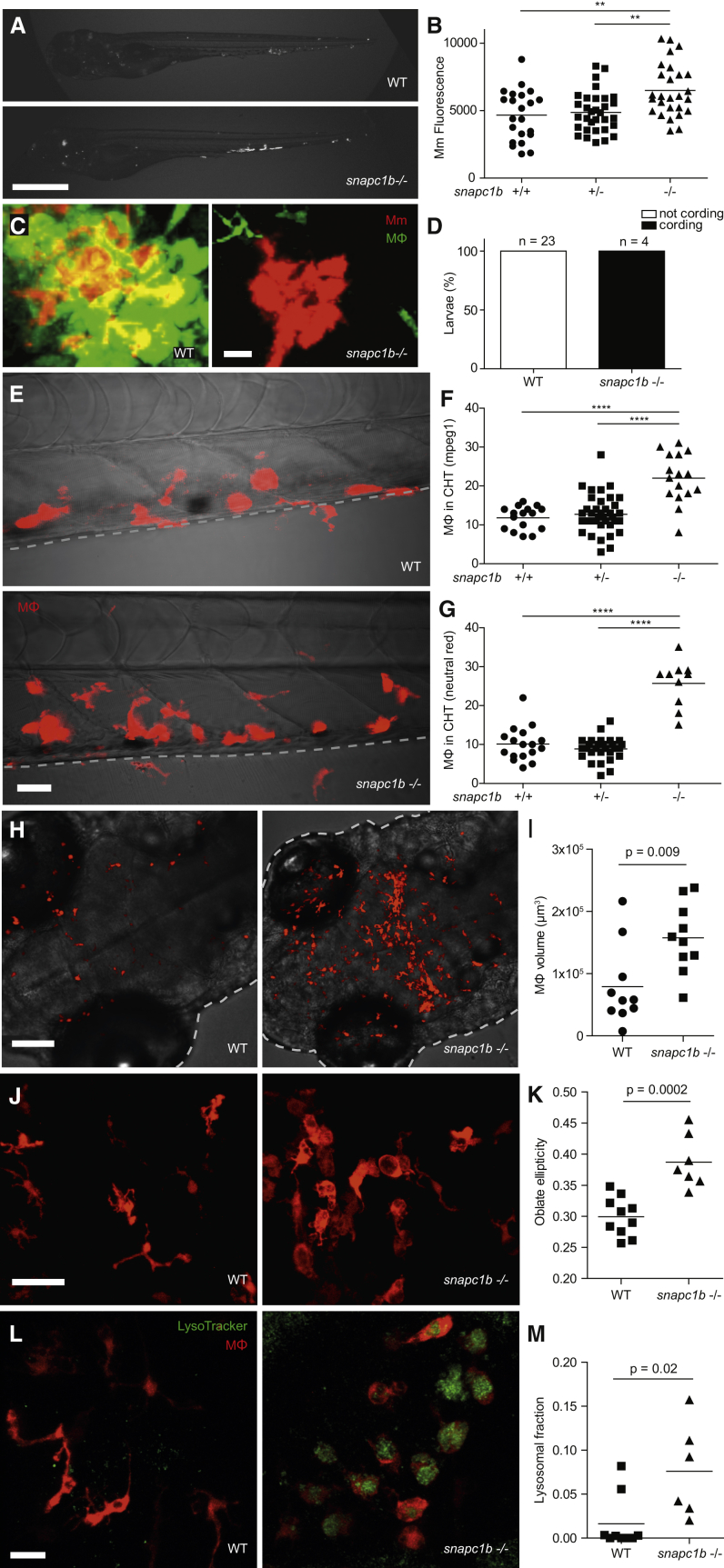
*snapc1b* Mutants Are Hypersusceptible to *M. marinum* and Have Increased Numbers of Macrophages that Display Vacuolated Morphology (A) Representative images of wild-type (WT) and *snapc1b*^*fh111/fh111*^ mutant larvae 4 days post-infection (dpi) with 150 *M. marinum* (Mm). Scale bar, 300 μm. (B) Quantification of Mm burden measured by fluorescence in *snapc1b*^*fh111/+*^ incross larvae at 5 dpi with 240 Mm. (C) Confocal images of green fluorescent macrophages (MΦ) and red fluorescent bacteria in intact granulomas of WT larvae and extracellular corded bacteria following complete granuloma breakdown in *snapc1b* mutant larva at 2 dpi with 200 Mm. Scale bar, 15 μm. (D) Quantification of bacterial cording in larvae from an incross of *snapc1b*^*fh111/+*^ parents at 5 dpi with 200 Mm. (E) Confocal images of the caudal hematopoietic tissue (CHT) of representative WT and *snapc1b* mutant larvae with red fluorescent macrophages at 6 days post-fertilization (dpf). Scale bar, 20 μm. (F and G) Quantification of fluorescent macrophages (F) and neutral red-stained cells (G) in the CHT of *snapc1b*^*fh111/+*^ incross larvae at 6 dpf. (H) Confocal images of fluorescent macrophages in the head of representative WT and *snapc1b* mutant larvae at 3 dpf. Dotted lines indicate the outline of larvae. Scale bar, 100 μm. (I) Total macrophage volume in the brains of WT and *snapc1b* mutant larvae at 5 dpf. Volumetric analysis performed from 3D confocal images on red fluorescence signal. (J) Confocal images of fluorescent macrophages in the brain of WT and *snapc1b* mutant larvae at 3 dpf. Scale bar, 60 μm. (K) Measurement of oblate ellipticity of macrophages in the brains of WT and *snapc1b* mutant larvae at 3 dpf. (L) Confocal images red fluorescent macrophages stained with LysoTracker green in the brains of 3 dpf WT and *snapc1b* mutant larvae. Scale bar, 30 μm. (M) Average lysosomal volume per animal normalized to total macrophage volume. Macrophage and lysosomal volumes were determined by volumetric analysis of red fluorescence (macrophages) and green fluorescence (lysosomes) in 3D confocal images. Statistical significance was assessed by one-way ANOVA with Sidak’s post-test (B, F, and G) or Student’s t test (I, K, and M). See also Figures S1 and S2, and Tables S2 and S3.

**Figure S1:**
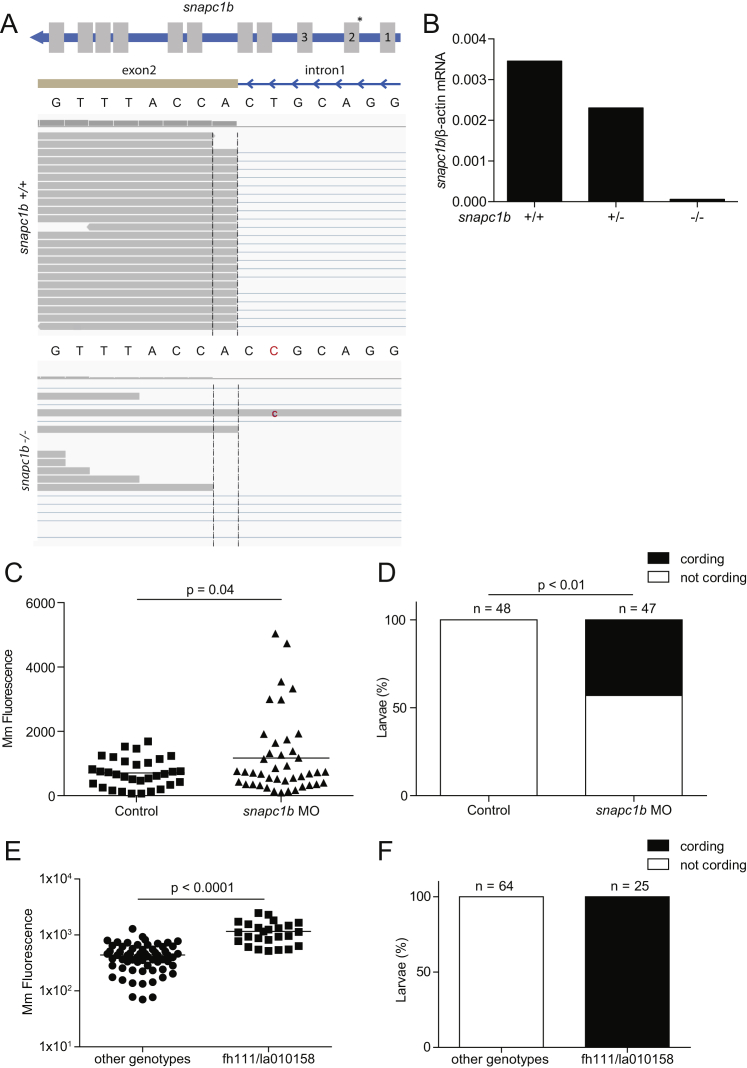
Genetic Disruption of the *snapc1b* Locus Confers Susceptibility to *M. marinum* Infection, Related to Figure 1 (A) Top: Diagram of *snapc1b* gene showing introns (blue), exons (gray), and location of the *fh111* splice acceptor mutation denoted by an asterisk above the relevant exon-intron boundary. Bottom: RNA-sequencing reads aligned to the exon 2 splice acceptor site from WT and *snapc1b*^*fh111/fh111*^ mutant larvae with wild-type and mutant sequence. The *snapc1b(fh111)* mutation is denoted in red. (B) Quantitative real-time PCR of properly spliced *snapc1b* transcript in *snapc1b*^*+/−*^ incross larvae at 6 dpf. Values normalized to transcript level of β*-actin*, representative of two experiments. (C and D) Quantification of bacterial burden (C) and cording (D) in control and morphant larvae at 4 dpi with 250 Mm. (E and F) Quantification of bacterial burden and cording in *snapc1b*^*Tg(la010158)/+*^ × *snapc1b*^*fh111/+*^ cross larvae at 5 dpi with 150 Mm. Statistical significance was assessed by Student’s t test (C,E) and Fisher’s exact test (D).

The zebrafish mutant *fh111*, identified in a forward genetic screen (Tobin et al., 2010), was hypersusceptible to *M. marinum*, displaying increased bacterial growth relative to wild-type siblings after intravenous infection (Figures 1A and 1B). *fh111* infection was characterized by the breakdown of forming granulomas accompanied by bacterial cording, a characteristic morphology acquired by rapidly growing extracellular bacteria after release from necrotic macrophages (Pagán et al., 2015, Tobin et al., 2010) (Figure 1C). We used bacterial cording as a sensitive and specific phenotype to map *fh111* (Figure 1D) (Tobin et al., 2010). *fh111* maps to a splice acceptor site mutation in the exon 1–2 junction of the zebrafish *snapc1b* gene on chromosome 13 (Figure S1A), one of two orthologs of human *SNAPC1* (Small Nuclear RNA Activating Complex Polypeptide 1) that encodes a component of the basal transcriptional machinery for RNA Pol II and III-dependent transcription (Henry et al., 1998). Zebrafish *snapc1b* has higher amino acid identity to human *SNAPC1* than its paralog *snapc1a*, located on chromosome 20 (39% versus 35%, respectively) (Flicek et al., 2014). RNA sequencing (RNA-seq) analysis of wild-type (WT) animals at 6 days post-fertilization (dpf) showed that *snapc1b* RNAs were ∼35-fold more abundant than *snapc1a* RNAs (GEO: GSE74196). We confirmed the location and transcriptional consequence of *fh111* by RNA-seq and qRT-PCR (Figures S1A and S1B). Causality of the *fh111* mutation was confirmed by a splice-blocking antisense oligonucleotide (morpholino) that targeted the same exon 1–2 splice junction of *snapc1b* (Table S1) that phenocopied *fh111* susceptibility (Figures S1C and S1D) and by non-complementation with an independent retroviral insertion allele that disrupts exon 1 of *snapc1b* (*la010158*) (Figures S1E and S1F) (Varshney et al., 2013).

In sum, our findings suggest that Snapc1b deficiency causes hypersusceptibility to mycobacterial infection through early granuloma breakdown, which releases mycobacteria into the extracellular milieu that is more growth permissive than the intracellular environment, culminating in bacterial cording morphology (Pagán et al., 2015).

### Macrophages of *snapc1b* Mutants Are Increased in Number and Have Enlarged Lysosomes

Granuloma breakdown can result from a global reduction in macrophage numbers available to replenish the granuloma (Pagán et al., 2015). We were surprised to find that, even in uninfected *snapc1b* mutants, macrophage numbers were increased as revealed by increased numbers of fluorescent macrophages in transgenic animals (Ellett et al., 2011) and by staining with neutral red, a vital dye that accumulates in macrophages (Davis and Ramakrishnan, 2009) (Figures 1E–1G). The increased abundance of microglia, tissue resident macrophages of the brain derived from a primitive hematopoietic lineage (Clements and Traver, 2013), suggested a derangement in multiple waves of myelopoiesis (Figures 1H and 1I).

**Figure S2:**
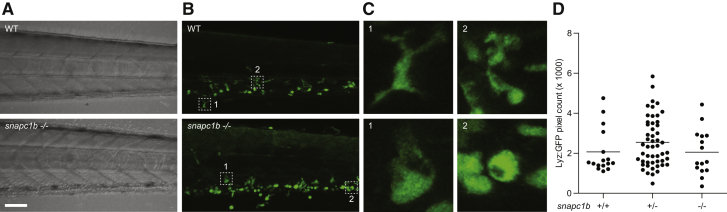
*snapc1b* Mutants Have Numerous Vacuolated Macrophages and Normal Neutrophil Numbers in the Caudal Hematopoietic Tissue, Related to Figure 1 (A and B) (A) Brightfield and (B) confocal images of the CHT of representative WT and snapc1b-/- mutant larvae at 5 dpf. Scale bar 50μm. (C) 8X magnification of outlined regions in (B) showing normal (top) and vacuolated (bottom) morphology. (D) Quantification of Lyz:eGFP positive, green fluorescent neutrophils in *snapc1b*^*+/−*^ incross larvae at 6 dpf.

Most of the macrophages in mutants were enlarged and discoid in shape (Figures 1J, 1K, and S2A–S2C). The enlarged macrophages contained prominent vacuoles, which were revealed to be fused lysosomes by staining with LysoTracker (Figure 1L) (Peri and Nüsslein-Volhard, 2008). The mutant lysosomes were larger than wild-type and occupied a greater proportion of total macrophage volume (Figure 1M). This aberrant macrophage morphology is similar to what has been observed in human lysosomal storage disorders (Kieseier et al., 1997). Our observation of an increased abundance of tissue resident macrophages (histiocytosis) accompanied by increased expression of myeloid growth factors (Table S2) also mirrors findings in several human lysosomal storage disorders (Allen, 2008, Hsu et al., 2012). Neutrophils, the other myeloid cells present at this developmental stage, were not increased in *snapc1b* mutants and displayed normal morphology, consistent with their lack of involvement in homeostatic scavenger functions (Figure S2D; data not shown).

### *snapc1b* Mutant Macrophages Become Vacuolated and Immotile as a Result of Lysosomal Accumulation of Undigested Cell Debris

**Figure 2:**
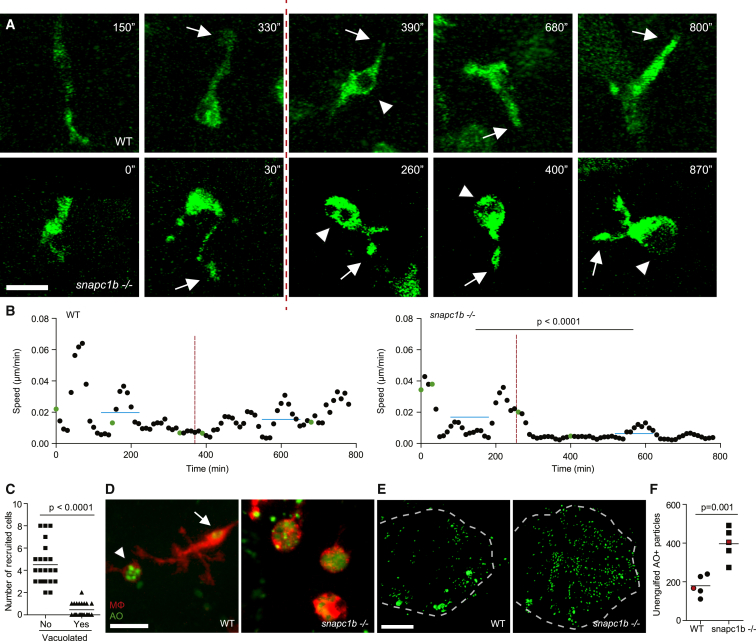
Lysosomal Storage in *snapc1b* Mutants Compromises Physiological Efferocytosis (A) Still images from confocal video of green fluorescent macrophages in *snapc1b* mutant larval and WT sibling brains. Time of image is indicated in minutes. Arrows mark pseudopodia; arrowheads mark vacuoles. Vertical dotted red line indicates the time point immediately following phagocytic event. Scale bar, 15 μm. (B) Speed of WT and *snapc1b* mutant macrophages from the confocal video in (A). Average speed before and after the phagocytic events are indicated by a horizontal blue line. Green dots correspond to time points in the images shown in (A). (C) Migration of normal and vacuolated macrophages from the same animal to CCL2 injected into the HBV. (D) Representative confocal image of red fluorescent macrophages stained with acridine orange (AO) in brains of *snapc1b* mutant larvae and WT siblings at 3 dpf. Arrow marks a wild-type macrophage with very little AO staining. Arrowhead marks a rare AO positive macrophages seen in WT brains. Scale bar, 30 μm. (E and F) Confocal images (E) and quantification (F) of green fluorescent acridine-orange-stained unengulfed cell debris in the brains of *snapc1b* mutant larvae and WT siblings at 5 dpf. Scale bar, 150 μm. Images in (E) denoted as red data points in (F). Statistical significance was assessed by Student’s t test (B and F) and paired t test (C). See also Figure S3.

To understand the development of vacuolated morphology, we compared microglial morphology and dynamics in uninfected animals during physiological efferocytosis of apoptotic neurons. In wild-type animals, macrophages assumed a vacuolated morphology upon phagocytosis of particulate material (presumably cell debris) but reverted to normal within 4 hr (Figure 2A; Movie S1). Vacuolated morphology was accompanied by a transient reduction in speed of homeostatic migration; movement resumed upon reversion to normal morphology (Figures 2A and 2B; Movie S1). In *snapc1b* mutants, macrophages became irreversibly vacuolated after only a few phagocytic events, accompanied by sustained immotility and consequently reduced displacement—36 μm versus 131 μm for wild-type—over a 13-hr observation period (Figures 2A and 2B; Movie S1). These vacuolated macrophages did not have obvious phagocytic defects and continued to extend pseudopods in apparent phagocytic events (Figure 2A; Movie S1). Vacuolated macrophages were also unable to migrate in response to the chemotactic molecule CCL2 injected into the hindbrain ventricle (HBV) (Cambier et al., 2014b), indicating a broad migratory defect toward both cell debris and chemotactic factors (Figure 2C). These findings are consistent with observations that macrophages from patients with Gaucher’s disease, the most common human lysosomal storage disorder, are defective for migration but competent for phagocytosis (Aflaki et al., 2014).

**Figure S3:**
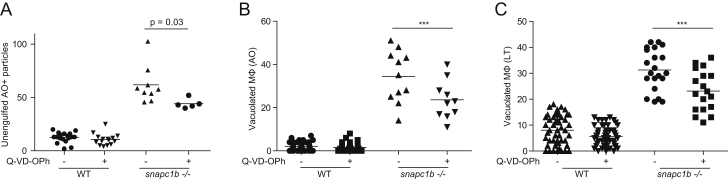
Global Inhibition of Apoptosis Reduces the Abundance of Vacuolated Macrophages in the *snapc1b* Mutant, Related to Figure 2 (A) Quantification of extracellular AO positive particles in WT and *snapc1b* mutant larvae at 3 dpf following treatment with 10 μM Q-VD-OPh or DMSO control. (B) Quantification of AO-positive vacuolated macrophages in *snapc1b* mutant larvae and WT siblings at 3dpf following treatment with 10 μM Q-VD-OPh or DMSO control. (C) Quantification of LysoTracker-positive macrophages in *snapc1b* mutant larvae and WT siblings at 3dpf following treatment with 50 μM Q-VD-OPh or DMSO.

Acridine orange staining confirmed that the lysosomal accumulations in mutant macrophages were phagocytosed apoptotic bodies (Abrams et al., 1993, Peri and Nüsslein-Volhard, 2008) (Figure 2D). Together, these findings suggested that lysosomal accumulation of undigested cell debris in mutant macrophages impairs migration irreversibly and leads to progressive macrophage incapacitation. If so, then we hypothesized that mutants would have an accumulation of extracellular apoptotic bodies in the brain as seen in several human lysosomal storage disorders (Huang et al., 1997). They did (Figures 2E and 2F). Furthermore, a global reduction in apoptosis induced by the pancaspase inhibitor Q-VD-OPh reduced extracellular cell debris (Figure S3A) and decreased the number of macrophages with lysosomal accumulations of the debris and with enlarged lysosomes (Figures S3B and S3C).

Together, these findings show that *snapc1b* mutant macrophages become irreversibly vacuolated due to their inability to degrade phagocytosed cell debris. As more and more macrophages lose their migratory capability, a deficit of functioning macrophages may develop.

### Macrophage Migration Deficit Underlies Granuloma Breakdown in *snapc1b* Mutants

We recently showed that reduction in the macrophage supply reduces granuloma macrophage replenishment to the point at which apoptotic infected macrophages, failing to be engulfed, undergo secondary necrosis (Pagán et al., 2015). Having observed that the vacuolated macrophages of *snapc1b* mutants failed to migrate to dying cells in the uninfected state and failed to migrate to newly infecting bacteria, we wondered whether they were also unable to migrate to dying cells in the tuberculous granuloma. If so, a functional macrophage deficiency could develop in the context of global macrophage excess, providing an explanation for our observation of early granuloma breakdown.

**Figure 3:**
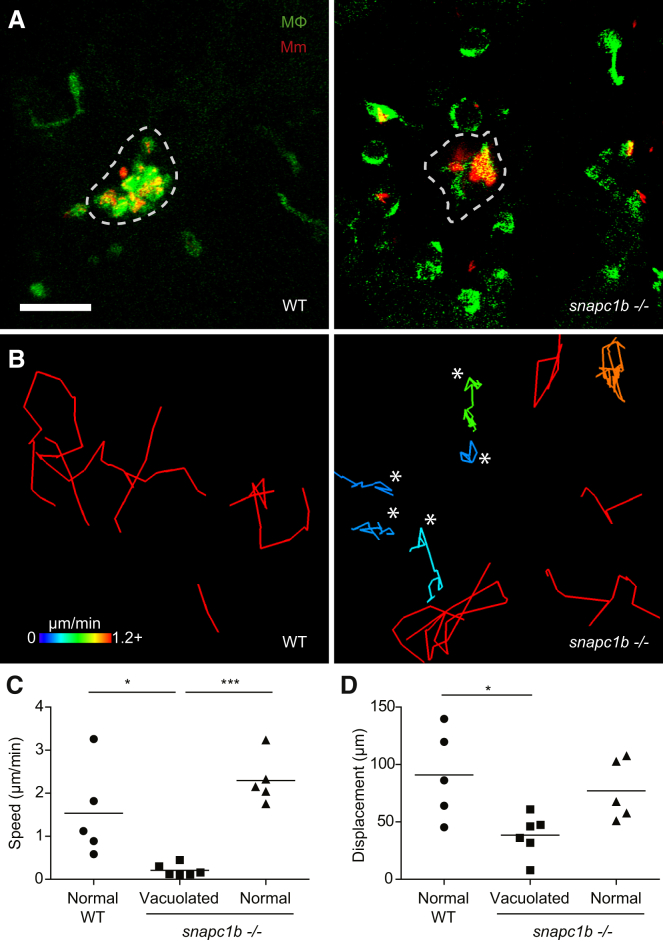
*snapc1b* Mutant Macrophages Fail to Participate in Granuloma Formation (A) Confocal images of granulomas in the hindbrain ventricle of *snapc1b* mutant larvae and WT siblings with green fluorescent macrophages at 2 dpi with 100 red fluorescent Mm. Scale bar, 60 μm. (B) Tracks of macrophage movement following granuloma formation in *snapc1b* mutant larvae and WT siblings shown in (A). Tracks are coded for speed. Tracks created by vacuolated macrophages are indicated with an asterisk. (C and D) Speed (C) and displacement (D) of *snapc1b* mutant and WT sibling macrophages in (A and B). Statistical significance was assessed using one-way ANOVA with Sidak’s post-test.

We performed detailed time-lapse confocal imaging of forming granulomas in wild-type and *snapc1b* mutant larvae over ∼18 hr. Wild-type granulomas retained cellularity over this period through continuous influx of macrophages (Movie S2; Figures 3A and 3B). In contrast, Snapc1b-deficient granulomas broke down soon after formation (Movie S2). Mutant granulomas were surrounded by mostly vacuolated macrophages that failed to migrate into the granuloma (Figures 3A and 3B; Movie S2). The migration deficit in *snapc1b* mutants was specific to the vacuolated macrophages; the morphologically normal macrophages in the mutants maintained displacements and speeds similar to those of wild-type animals (Figures 3C and 3D; Movie S2). Thus, macrophage lysosomal storage leads to granuloma breakdown by preventing migration to the forming structure and is functionally equivalent to a global macrophage deficiency. Our findings suggest that, once the *snapc1b* mutants have exhausted their migration-competent macrophages, the tuberculous granuloma breaks down resulting in bacterial cording.

### Lysosomal Cathepsin Deficiency Underlies *snapc1b* Mutant Macrophage Abnormalities and Hypersusceptibility to *M. marinum*

**Figure 4:**
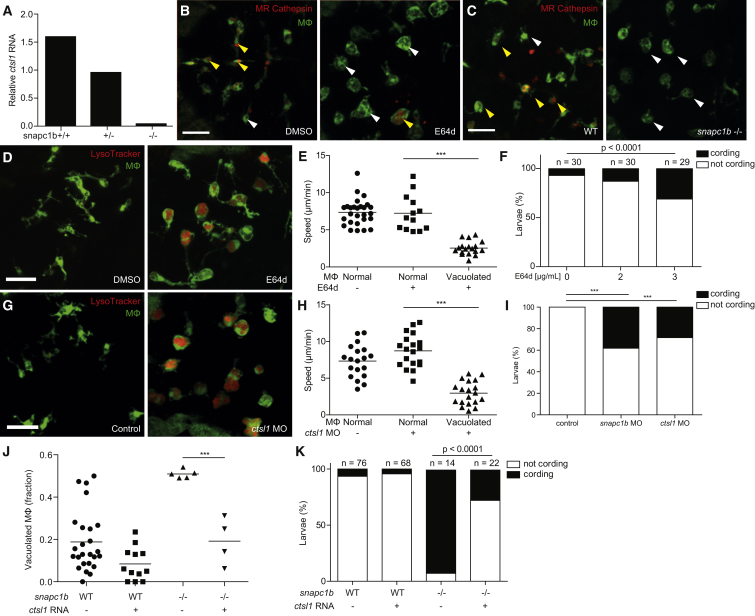
Cathepsin L Deficiency Causes *snapc1b* Mutant Vacuolated Macrophage Morphology and Susceptibility to *M. marinum* (A) Quantitative real-time PCR of relative *ctsl1* transcript in *snapc1b*^*+/−*^ incross larvae at 6 dpf. Values normalized to transcript level of the heterozygous larvae, representative of two experiments. (B and C) Confocal images of green fluorescent macrophages in larvae injected with red fluorescent MR-Cathepsin L at 3 dpf, either following treatment with E64d or DMSO control at 2dpf (B) or in *snapc1b* mutants and WT siblings (C). Yellow or white arrowheads denote macrophages that are positive or negative for MR-Cathepsin, respectively. Scale bar, 50 μm. (D) Confocal images of green fluorescent macrophages stained with LysoTracker red in the brains of 3-dpf E64d-treated and DMSO control larvae. Scale bar, 50 μm. (E) Average macrophage speeds during a 5-hr movie in the brains of 3-dpf E64d-treated and DMSO control larvae. (F) Quantification of bacterial cording in DMSO control and E64d-treated larvae at 5 dpi with 150 Mm. (G) Confocal images of green fluorescent macrophages stained with LysoTracker red in the brains of 3-dpf *ctsl1* morphants and control larvae. Scale bar, 50 μm. (H) Average macrophage speeds during a 5-hr movie in the brains of 3-dpf *ctsl1* morphants and control larvae. (I) Quantification of bacterial cording in control, *snapc1b*, and *ctsl1* morphants at 5 dpi with 200 Mm. (J) Quantification of vacuolated macrophages in the brains of 3-dpf WT or *snapc1b* mutant larvae following injection of *ctsl1* RNA or control at 0 dpf. (K) Quantification of bacterial cording at 2 dpi with 215 Mm in the HBV of *snapc1b* mutants and WT siblings following injection of *ctsl1* RNA or control. Statistical significance was assessed by ANOVA with Sidak’s post test (E, H, and J) or Fisher’s exact test (F, I, and K). See also Figure S4.

Human SNAPC1 is involved in global RNA polymerase II-dependent transcription (Baillat et al., 2012). RNA-seq analysis of *snapc1b* mutants and wild-type siblings revealed more than 1,000 differentially expressed genes in the mutant (Table S3), suggesting zebrafish Snapc1b functions in a similar manner. How might a broadly acting transcriptional regulator produce such specific phenotypes? Guided by the *snapc1b* mutant phenotype, we analyzed the RNA-seq dataset for lysosomal genes including those associated with human lysosomal storage disorders (Table S2) (Platt et al., 2012). Only two, the myeloid cell-specific lysosomal cysteine cathepsins B and L1 (*ctsbb* and *ctsl1*) (Heng et al., 2008), were underrepresented in the mutant, at 9% and 13% of wild-type levels, respectively (Table S2), and we confirmed their commensurate reduction by qRT-PCR analysis (85% and 83%, respectively) (Figure 4A; data not shown). We were able to test lysosomal cathepsin activity in situ using MagicRed (MR)-Cathepsin L, a modified cathepsin L target sequence, which fluoresces only when cleaved (Peri and Nüsslein-Volhard, 2008). In wild-type animals, brain macrophages quickly cleaved injected MR-cathepsin L; this number was reduced as expected by administration of the irreversible pan-cysteine cathepsin inhibitor, E64d (42.7% of macrophages in control versus 3.63% in E64d-treated larvae, p < 0.0001) (Murray et al., 1997) (Figure 4B). *snapc1b* mutants displayed reduced MR-cathepsin-L cleavage compared to wild-type siblings, indicative of reduced lysosomal cathepsin L activity (47.2% of macrophages in WT animals versus 5.3% in mutants, p < 0.0001) (Figure 4C).

We next tested whether cysteine cathepsin deficiency underlies all of the *snapc1b* mutant phenotypes. Inhibition of cysteine cathepsins by E64d recapitulated both baseline and infected *snapc1b* mutant phenotypes—macrophage lysosomal storage with accompanying migratory defects at baseline, and hypersusceptibility to infection with bacterial cording (Figures 4D–4F). We attempted morpholino knockdown of *ctsbb* and *ctsl1* to probe their individual culpabilities in the *snapc1b* mutant phenotypes (Table S1). As the *ctsbb* morpholino was highly toxic, we could only pursue *ctsl1* further. *ctsl1* morphants recapitulated the *snapc1b* phenotypes (Figures 4G–4I). Transient overexpression of *ctsl1* mRNA in *snapc1b* mutant larvae restored normal macrophage morphology in uninfected *snapc1b* mutants and rescued cording (Figures 4J and 4K). Together these experiments implicate cysteine cathepsins in *snapc1b* hypersusceptibility resulting from macrophage incapacitation. Our data ascribe a substantial portion of *snapc1b* phenotypes to cathepsin L1 deficiency though we cannot rule out a minor role for cathepsin B deficiency. Prior findings that cathepsin L knockout mice are not hypersusceptible to *M. tuberculosis* (Nepal et al., 2008) may reflect functional redundancies present in the mouse but not the zebrafish.

**Figure S4:**
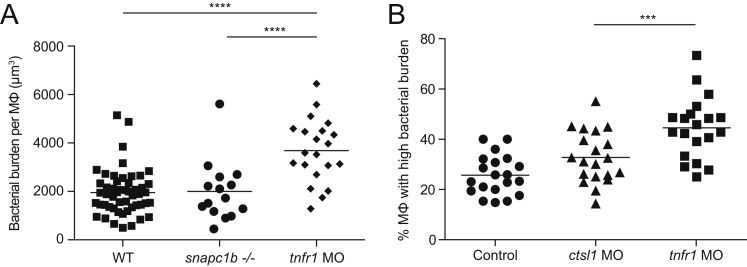
Macrophages of *snapc1b* Mutant and cathepsin L-Deficient Animals Restrict Mycobacterial Growth Normally, Related to Figure 4 (A) Macrophage intracellular bacterial burdens of *snapc1b*^*+/−*^ incross larvae and *tnfr1* morphants infected with 100 red fluorescent Mm at 2 dpf in the caudal vein. Bacterial volume (μm^3^) was quantified per animal from 3D confocal images captured in the tail region at 40 hpi. The intramacrophage replication of Mm is unrestricted in *tnfr1* morphants as expected (Clay et al., 2008; Pagán et al., 2015, Tobin et al., 2010). (B) Percentage of macrophages with high intracellular bacterial burdens in control, *ctsl1* and *tnfr1* morphants infected with ∼75 red fluorescent Mm at 2 dpf in the caudal vein. Bacterial burden was quantified per animal by counting the average number of bacteria per macrophage and categorizing as low (1-5 bacteria) or high (> 5 bacteria). Statistical significance was assessed by one-way ANOVA with Sidak’s post test (A, B).

Our finding that cathepsin L1 deficiency mediated hypersusceptibility prompted us to ask whether this lysosomal hydrolase might play a role in macrophage microbicidal activity for two reasons. First, a deficit in macrophage microbicidal activity (e.g., through TNF deficiency) has been shown to result in granuloma breakdown with bacterial cording (Tobin et al., 2010). Second, in vitro, cathepsin L has been reported to indirectly facilitate mycobacterial killing by cleaving ubiquitin into microbicidal peptides (Alonso et al., 2007). However, we found that macrophages of both *snapc1b* mutants and *ctsl1* morphants restricted bacterial growth normally (Figure S4). These findings suggest that cathepsin L-mediated macrophage microbicidal capacity is dispensable in vivo and confirms that its deficiency induces susceptibility by compromising macrophage migration.

### Zebrafish Models of Human Lysosomal Storage Disorders Display Accelerated Tuberculous Granuloma Breakdown

**Figure 5:**
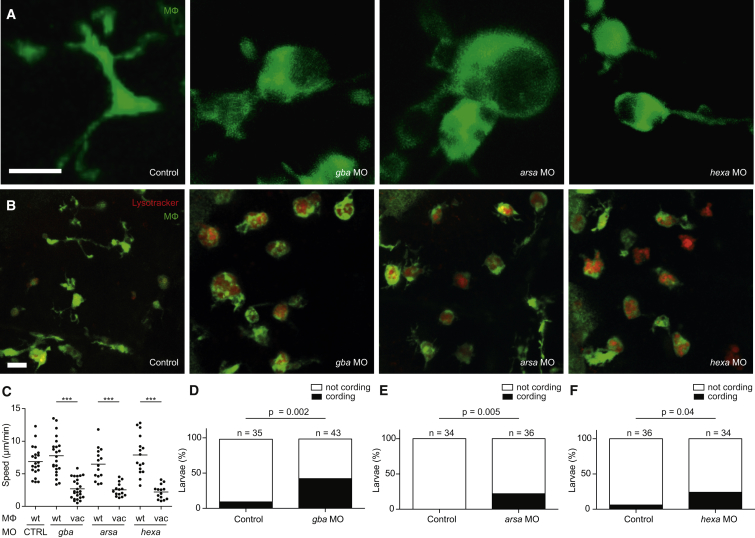
Lysosomal Storage Disorders Disrupt Macrophage Migration and Cause Granuloma Breakdown (A and B) Confocal images of green fluorescent macrophages in the brain of 3-dpf control and morphant larvae, unstained (A) or following staining with LysoTracker Red (B). Scale bars, 10 μm. (C) Quantification of average macrophage speed in control and morphant larvae by macrophage morphology (wt, wild-type; vac, vacuolated). (D–F) Quantification of bacterial cording in control and morphant larvae at 3 dpi with 200 Mm. Statistical significance was determined using paired t tests with Bonferroni correction (C) and Fisher’s exact test (D–F).

While cathepsin deficiency causes protein accumulation in lysosomes, many human genetic lysosomal storage disorders result from the accumulation of diverse lipid species (Platt et al., 2012). Patients with Gaucher’s disease, the most common lysosomal storage disease, have macrophages with migration defects in vitro (Aflaki et al., 2014, Liel et al., 1994) and are susceptible to a variety of pathogens including mycobacteria, though this may be due to concomitant immune defects including pancytopenias (Aker et al., 1993, Jain and Yelwatkar, 2011, Machaczka et al., 2014, Zimran, 2011). We asked whether the mechanism of susceptibility uncovered for *ctsl1* deficiency extended to lysosomal storage disorders characterized by lipid accumulation. Knockdown of the zebrafish orthologs of the genes responsible for Gaucher’s disease, Tay-Sachs disease, and metachromatic leukodystrophy produced increased numbers of vacuolated macrophages with enlarged lysosomes and migratory defects (Tables S1 and S2; Figures 5A–5C). Upon infection, all three exhibited early granuloma breakdown and bacterial cording (Figures 5D–5F). Thus, etiologically diverse lysosomal storage disorders can increase susceptibility to tuberculous infection, regardless of the nature of the accumulated material.

### Macrophage Lysosomal Storage Disrupts Endocytic Recycling

Our work so far had linked macrophage lysosomal storage to impaired migration to increased susceptibility to mycobacteria. Having understood the cellular basis of the link between impaired macrophage migration and susceptibility, we sought to understand how macrophage lysosomal storage might impair migration. Recycling between the endosomal and plasma membranes is known to be required for cell migration. This recycling delivers membrane lipids and proteins required for movement to the plasma membrane and facilitates adjustments in cell-surface area that are critical for cell motility (Bretscher and Aguado-Velasco, 1998, Traynor and Kay, 2007, Veale et al., 2010). Lysosomes share contents with endosomes, and recent evidence suggests that, like endosomes, they participate in recycling to the plasma membrane (Bright et al., 2005, Bright et al., 2015). Accordingly, embryonic fibroblasts isolated from two mouse models of severe lysosomal storage disorders display broad dysregulation of the entire endocytic pathway (Fraldi et al., 2010).

We asked whether endocytic recycling was disrupted in the zebrafish macrophages with lysosomal storage by monitoring the fate of fluorescently labeled high-molecular-weight dextran (10,000 MW) in normal animals and those with macrophage lysosomal storage. Following endocytosis, high-molecular-weight dextran is trafficked to lysosomes but not readily degraded, and its loss from lysosomes strictly reflects trafficking from them. In pulse-chase experiments, dextran-labeled lysosomes have been shown to fuse rapidly with endosomes, and several hours later the dextran is released into the extracellular medium suggesting subsequent fusion events that involve trafficking to the plasma membrane (Bright et al., 2015).

**Figure 6:**
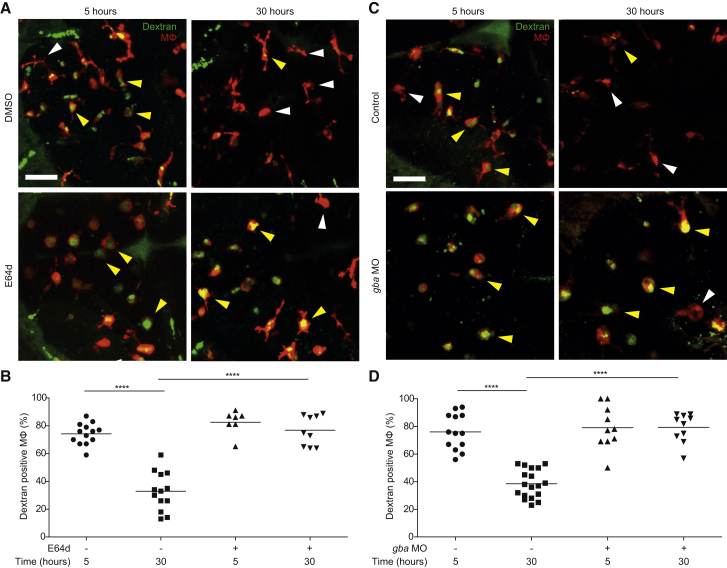
Macrophage Lysosomal Storage Disrupts Endocytic Recycling (A) Confocal images of red fluorescent macrophages following injection of green fluorescent dextran in E64d-treated and DMSO control larvae (3 dpf) at 5 and 30 hr post-injection. Yellow and white arrowheads denote macrophages with and without dextran, respectively. Scale bar, 50 μm. (B) Quantification of the percentage of macrophages that are positive for dextran in E64d-treated and DMSO control larvae (3 dpf) at 5 and 30 hr post-injection. (C) Confocal images of red fluorescent macrophages following injection of green fluorescent dextran in *gba* morphants and control larvae (3 dpf) at 5 and 30 hr post-injection. Yellow and white arrowheads denote macrophages with and without dextran, respectively. Scale bar, 50 μm. (D) Quantification of the percentage of macrophages that are positive for dextran in *gba* morphants and control larvae (3 dpf) at 5 and 30 hr post-injection. See also Figure S5.

We injected fluorescent dextran into the brains of 3-dpf zebrafish larvae—wild-type, cathepsin deficient by E64d treatment, and *gba*-deficient morphants. In all groups, 74%–82% of the macrophages had taken up the dye within 5 hr (Figure 6). After 30 hr, only 33% and 39% of the macrophages in the wild-type fish retained the dextran, whereas 77% and 79% did in the cathepsin and *gba*-deficient animals, respectively (Figure 6), suggesting that stalling of the entire endocytic system is a common feature of lysosomal storage diseases and underlies the defective migration displayed by vacuolated macrophages.

**Figure S5:**
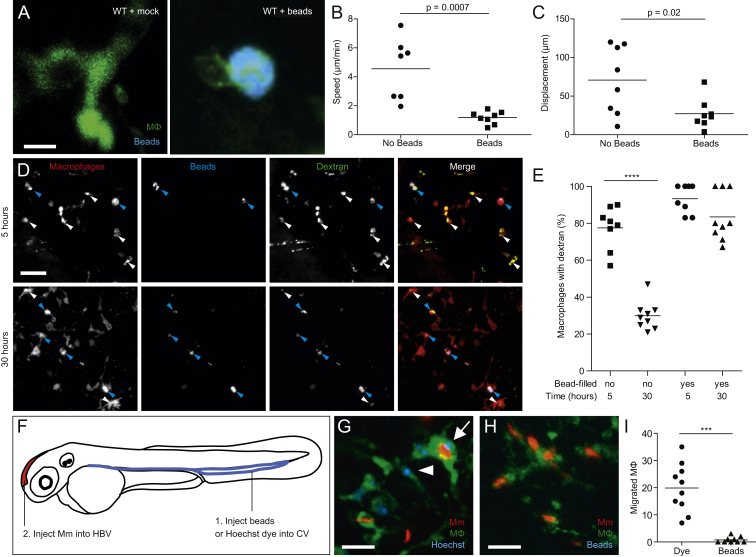
Lysosomal Accumulation of Inert Particles Compromises Endocytic Recycling and Migration to the Initial Site of Mycobacterial Infection, Related to Figure 6 (A) Confocal images of green fluorescent macrophages in larvae mock-injected or injected with 5x10^5^ blue fluorescent 1 μm polystyrene beads. Scale bar, 12 μm. (B and C) Speed (B) and displacement (C) of macrophages with and without beads. (D) Confocal images of red fluorescent macrophages in 3dpf larvae pre-loaded with blue fluorescent polystyrene beads as in (A), injected 12 hr later with green fluorescent dextran and imaged at 5 and 30 hr post-dextran injection. Blue and white arrowheads denote macrophages containing dextran, with and without blue beads, respectively. Scale bar, 50 μm. (E) Quantification of macrophages that retained dextran at 5 and 30 hr post injection. (F) Diagram showing the experimental outline in which 2 dpf larvae were injected with Hoechst dye or beads in the CV followed by infection in the HBV with 200 Mm. (G and H) Confocal images of larval HBV containing green-fluorescent macrophages following CV injections with Hoechst (G) or blue fluorescent beads (H). Arrow and arrowhead denote Hoechst-positive macrophages that have migrated from the CHT, with and without phagocytosed red fluorescent Mm, respectively. Scale bar, 10 μm. (I) Number of macrophages in the HBV after injection of dye or beads in the CV followed by Mm infection in the HBV. Statistical significance was assessed using Student’s t test (B, C, and I), and one-way ANOVA with Sidak’s post test (E).

Our finding that the lysosomal accumulation of diverse biomolecules compromises endocytic recycling, and thus cell motility, suggested a common mechanism independent of the specific lysosomal substrate. If so, then lysosomal storage induced by non-biological particles should produce the same phenotypes. We injected beads into the HBV, which were phagocytosed by brain resident macrophages (Figure S5A). Bead-laden macrophages were compromised for homeostatic migration and exhibited disruption of endocytic recycling (Figures S5B–S5E).

### Lysosomal Accumulation Compromises Macrophage Migration to Newly Infecting Mycobacteria

In addition to their role in forming and maintaining the granuloma, resident macrophages are the first cells to migrate to mycobacteria at the initial site of infection (Cambier et al., 2014a, Philips and Ernst, 2012). This first macrophage-mycobacterium interaction can be visualized in the zebrafish hindbrain ventricle (HBV), a cavity into which phagocytes migrate in response to mycobacteria (Cambier et al., 2014b). In the *snapc1b* mutant, only the subset of brain-resident macrophages that still had normal morphology migrated to the bacteria and phagocytosed them, while the vacuolated macrophages, failing to migrate from the adjacent brain parenchyma, remained uninfected (data not shown). We could not directly test the migration of bead-laden brain resident macrophages, as only a minority of them engulfed sufficient numbers of beads injected into the HBV. So we injected either beads or the nuclear stain Hoechst 33342 into the caudal vein followed by bacteria into the HBV (Figure S5F). As observed previously, the Hoechst-stained macrophages could be discerned by their blue nuclei and were morphologically normal (Figure S5G) (Davis and Ramakrishnan, 2009). After confirming that similar numbers of circulating macrophages were labeled blue by either dye or beads, we injected bacteria into the HBV (Figure S5F). Multiple Hoechst-positive macrophages migrated to the HBV in response to the bacteria, as expected (Davis and Ramakrishnan, 2009), but hardly any bead-filled ones did (Figures S5G–S5I). Thus, the accumulation of indigestible inert particles in macrophage lysosomes compromises their migration so as to preclude their ability to phagocytose infecting mycobacteria.

### Lysosomal Accumulation of Tobacco Smoke Particulates Compromises Macrophage Migration to *M. tuberculosis* in Humans

Human TB is thought to begin when mycobacteria are phagocytosed by pulmonary alveolar macrophages, the resident macrophages at the air-lung interface (Bates et al., 1965, Hocking and Golde, 1979, Ratcliffe and Wells, 1948, Verrall et al., 2014). Consistent with their role in primary defense against diverse inhaled bacteria (Green and Kass, 1964, Hocking and Golde, 1979), the ability of a substantial number of individuals to clear *M. tuberculosis* early after infection has been ascribed to the microbicidal activity of the alveolar macrophage (Verrall et al., 2014). Despite their central defensive role, many alveoli are normally devoid of macrophages because their numbers are limiting (Betz et al., 1993, Ferin, 1982). Therefore, efficient and complete phagocytosis of inhaled particulates is predicated on the rapid migration of alveolar macrophages from nearby alveoli (Lehnert, 1992, Peão et al., 1993). This migration should be particularly relevant to TB, the outcome of which depends upon the fate of the 1-3 bacteria deposited in a distal alveolus, which might not contain a macrophage (Bates et al., 1965, Ratcliffe and Wells, 1948). In light of our findings that bead-laden macrophages were compromised for migration to newly infecting bacteria in the zebrafish, we wondered whether the accumulation of tobacco smoke particulates in the alveolar macrophages of cigarette smokers (Harris et al., 1970, Martin, 1973) might be similarly compromised, accounting for the poorly understood association between smoking and the acquisition of new TB infection (Anderson et al., 1997, den Boon et al., 2005). If an infecting mycobacterium were to be deposited in a macrophage-deficient alveolus and not rapidly phagocytosed by nearby alveolar macrophages rendered immotile secondary to lysosomal engorgement, it would have an extended period of extracellular growth before engulfment by alveolar macrophages or other myeloid cells recruited from afar.

**Figure 7:**
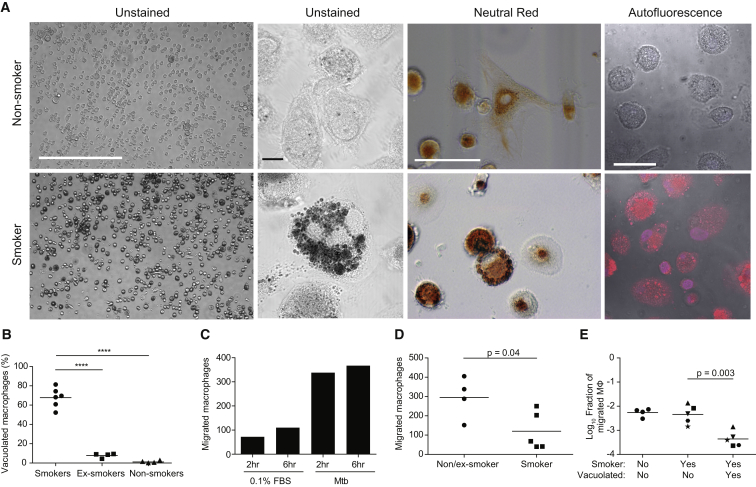
Lysosomal Accumulation of Tobacco Smoke Products in Alveolar Macrophages Compromises Migration to *M. tuberculosis* (A) Representative images showing the characteristics of macrophages isolated by bronchoalveolar lavage from smokers and nonsmokers. Scale bars (left to right), 400, 10, 20, and 10 μm. (B) Percentage of vacuolated macrophages was assessed in smokers, ex-smokers, and nonsmokers. Vacuolated macrophages were scored based on their autofluorescence and morphology. (C) Number of macrophages that migrated through a transwell was assessed at 2 and 6 hr of incubation with either 0.1% fetal bovine serum (FBS) or Mtb H37Ra using macrophages from an ex-smoker. Values represent averages of a single experiment performed in triplicate. (D) Number of macrophages from non/ex-smokers that migrated through a transwell toward Mtb H37Ra (assessed following 2 hr incubation). (E) Fraction of macrophages that migrated in the transwell assay calculated from initial versus migrated macrophages of each morphology. Samples from smokers are split into vacuolated and normal with unique symbols for each patient. Statistical significance was assessed by one-way ANOVA with Sidak’s post-test (B), Student’s t test (D), paired t test (E). See also Figure S6 and Table S4.

We examined alveolar macrophages obtained from smokers, nonsmokers, and ex-smokers by bronchoalveolar lavage (O’Leary et al., 2014) (Table S4; Supplemental Experimental Procedures). The majority of smokers’ alveolar macrophages exhibited vacuolated morphology and had accumulated opaque material in large lysosomal inclusions as evidenced by staining with neutral red, a vital dye that concentrates in lysosomes (Figures 7A and 7B). The abnormal cells were readily identified by their autofluorescence, consistent with previous findings (Martin, 1973). These cells were present at a lower frequency in ex-smokers and virtually absent in nonsmokers (Figures 7A and 7B).

**Figure S6:**
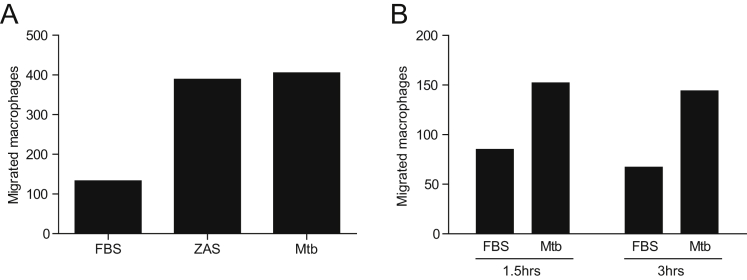
Alveolar Macrophage Migration to ZAS and Mtb, Related to Figure 7 (A) Migration of macrophages from nonsmoker SJH209 to 0.1% FBS, ZAS or Mtb at 2 hr. (B) Migration of macrophages from ex-smoker to Mtb at 1.5 and 3 hr in transwell assay.

Using a transwell assay, we confirmed prior reports that alveolar macrophages from nonsmokers and ex-smokers migrate to zymosan-activated serum, a rich source of the chemoattractant C5a (Figure S6A) (Barlow et al., 2008, Sweeney et al., 2015). In this assay, nonsmokers’ and ex-smokers’ alveolar macrophages also migrated to *M. tuberculosis* within 2 hr (Figures 7C and S6B). Migration of smokers’ macrophages to *M. tuberculosis* was impaired (Figure 7D). Our hypothesis predicts that this overall migration impairment is due to a selective inability of the vacuolated subset to migrate. Indeed, by calculating the fraction of the smokers’ normal versus vacuolated macrophages that migrated, we found that the migration impairment was specific to the vacuolated subset (Figure 7E). In sum, we show that the majority of smokers’ macrophages fail to migrate toward *M. tuberculosis* due to lysosomal accumulation of particulates, and their non-participation may contribute to the susceptibility of these individuals to TB.

## Discussion

We have described a zebrafish mutant in the *snapc1b* basal transcription factor component that displays the hallmark characteristics of human lysosomal storage disorders and is hypersusceptible to *M. marinum* infection. RNA-seq of *snapc1b* mutants revealed reduced expression of the lysosomal degradative cathepsins L and B, and pharmacological inhibition of cathepsin activity or knockdown of cathepsin L recapitulates the key mutant phenotypes of vacuolated macrophage morphology and susceptibility to infection.

Though cathepsin L is involved in the lysosomal degradation of phagocytosed material, its deficiency here mediates susceptibility to mycobacteria not by reducing macrophage microbicidal capacity but rather by causing lysosomal accumulation of undigested cell debris. This disrupts endocytic membrane recycling and thereby compromises macrophage migration in a variety of contexts. By modeling human lysosomal storage diseases in the zebrafish, we find that the accumulation of diverse substrates causes susceptibility to infection through this same mechanism. Our studies provide insights into the fundamental and common role played by macrophages as scavengers of dying cells during homeostasis and during tuberculous granuloma maintenance. These insights shed light on the protective role of tissue macrophages in early tuberculous infection and how lysosomal accumulation of tobacco smoke products may compromise this role.

### Macrophage Migration Defects Due to Lysosomal Accumulation of Undigested Cell Debris Contribute to the Pathogenesis of Lysosomal Storage Disorders

Sequential live visualization of the developing *snapc1b* mutant highlights the continuous scavenging role of macrophages under homeostatic conditions. Our work suggests that the accumulation of undigested cell debris in macrophage lysosomes may itself contribute substantially to the pathogenesis of human lysosomal storage diseases. We find that defects in macrophage degradative function render the cell vacuolated, immotile, and unable to further perform a critical scavenging function, which depends on directed migration to the dying cell (Hochreiter-Hufford and Ravichandran, 2013). This may contribute to the accumulation of unphagocytosed debris from cells undergoing apoptosis in the course of homeostatic tissue remodeling and repair, and the pathological consequences of their secondary necrosis.

The increased number of apoptotic bodies observed in human lysosomal storage disorders has been attributed to increased cell death triggered by the accumulation of lysosomal substrates (Huang et al., 1997). However, macrophages also accumulate lysosomal substrates in a variety of human lysosomal storage diseases (Kieseier et al., 1997); our findings suggest that the resultant immotility of an increasing proportion of macrophages may contribute to the accumulation of dead cells. Because tissue turnover is high in the developing brain, the macrophage scavenging deficit we propose may be particularly relevant for the pathogenesis of the neurological manifestations of lysosomal storage disorders hitherto attributed to neuronal dysfunction (Jeyakumar et al., 2005). Hematopoietic stem cell transplants in humans and mice improve clinical manifestations of lysosomal storage disorders, including neurological ones that are recalcitrant to enzyme replacement therapy (Biffi et al., 2004, Malatack et al., 2003, Norflus et al., 1998). In light of our findings, we speculate that hematopoietic stem cell transplantation alleviates disease pathology by restoring macrophage degradative function and consequently migration to engulf cell debris.

### Macrophage Migration Defects Caused by Lysosomal Accumulation Promote Tuberculous Granuloma Breakdown

Our studies of the *snapc1b* mutant, in which vacuolated macrophages fail to migrate into the tuberculous granuloma, reveal the inextricable link between macrophage homeostatic and immune function. Like the brain, the forming tuberculous granuloma is an environment with high cell turnover and the maintenance of its cellularity depends on the continuous migration of new macrophages that engulf dying infected macrophages (Davis and Ramakrishnan, 2009, Pagán et al., 2015). In the context of tissue remodeling, the clearance of dying cells prevents their secondary necrosis and release of inflammatory material into the extracellular space (Hochreiter-Hufford and Ravichandran, 2013). Likewise, in the TB granuloma, timely engulfment of dying infected macrophages prevents their secondary necrosis and release of bacteria into the extracellular milieu (Pagán et al., 2015). Granuloma breakdown is clinically significant because it increases both disease severity and risk of transmission (Cambier et al., 2014a).

Human lysosomal storage disorders are rare and often lethal within the first year of life, and thus unlikely to be significant contributors to the global burden of TB. Likewise, *snapc1b* mutant zebrafish fail to reach adulthood, and *SNAPC1*-null mutations in humans are likely embryonic lethal. However, even relatively small reductions in the macrophage supply to the granuloma can accelerate its breakdown (Pagán et al., 2015). It is possible that subtle alterations in macrophage degradative function, caused by altered expression of SNAPC1, or lysosomal cathepsins or other hydrolases, could create local macrophage deficits and increased susceptibility to TB. Thus, macrophage lysosomal accumulation from diverse genetic etiologies may together be not insignificant contributors to the global TB burden.

### Lysosomal Accumulation in Alveolar Macrophages of Smokers May Contribute to TB Susceptibility

Finally, our findings that lysosomal storage also compromises the migration of lung resident alveolar macrophages to mycobacteria suggests a mechanism for the observed susceptibility of smokers to new TB infection. There is accumulating evidence for a role for alveolar macrophages being first-responding protective cells in TB. In mice, aerosolized *M. tuberculosis* is found almost exclusively in alveolar macrophages for the first 7 days, after which infection moves into other myeloid cells such as monocytes and dendritic cells recruited from the lung interstitium or circulation (Srivastava et al., 2014, Urdahl, 2014). Their greater microbicidal capacity is mirrored in humans whose alveolar macrophages inhibit *M. tuberculosis* growth in contrast to peripheral blood monocytes, which are growth permissive (Aston et al., 1998). Our findings suggest that migration defects resulting from macrophage lysosomal engorgement impede the rapid engulfment (and therefore eradication) of infecting microbes at points of entry and may therefore facilitate bacterial entry into growth-permissive cells.

Cigarette smoking increases not only the risk of progression to active pulmonary TB disease, but also the risk of new TB infection, suggesting defective early response mechanisms in smokers (Anderson et al., 1997, den Boon et al., 2005, Gyawali et al., 2012). Smokers’ alveolar macrophages phagocytose bacteria and yeast normally and have normal bactericidal activity against *M. tuberculosis* (Cohen and Cline, 1971, Harris et al., 1970, O’Leary et al., 2014). The incapacitation of alveolar macrophages by tobacco smoke particulates may contribute to increased risk of infection in two ways: (1) delayed time to phagocytosis by the alveolar macrophage, allowing for a longer extracellular growth period by the bacteria, and (2) increased chance of initial phagocytosis by a recruited, growth-permissive macrophage.

In addition to providing an explanation for the increased susceptibility of individuals with genetic lysosomal storage disorders to respiratory, skin, and mucosal infections (Jain and Yelwatkar, 2011, Machaczka et al., 2014), our findings may constitute a basis for the susceptibility of smokers to other respiratory infections (Bagaitkar et al., 2008, Lin et al., 2007), Finally, this mechanism may also contribute to the poorly understood association between indoor air pollution and TB (Sumpter and Chandramohan, 2013).

Smokers’ increased susceptibility to infection may be reversible. A longitudinal study of alveolar macrophages after transplant of a smoker’s lung into a nonsmoker revealed a progressive decrease in “smokers alveolar macrophages” from >90% to 3% in 3 years (Marques et al., 1997). In our cohort, not only did ex-smokers have significantly fewer alveolar macrophages with lysosomal storage than smokers, but overall migration to *M. tuberculosis* was restored. These findings provide an additional rationale for smoking cessation as a prescription for TB prevention.

## Experimental Procedures

Detailed methods and bacterial and zebrafish strains associated with all procedures below are available in Supplemental Experimental Procedures.

### Zebrafish Husbandry and Larval Injections

Zebrafish husbandry and experiments were conducted according to guidelines from the UK Home Office, and the US NIH (approved by the University of Washington Institutional Animal Care and Use Committee). The wild-type AB strain was used for experiments except those in which the *snapc1b(fh111)* line or transgenic lines were used. Unless noted, crosses using *snapc1b(fh111)* were performed as heterozygote incrosses, which were genotyped at the completion of the experiment, to ensure blinded scoring of phenotypes. Except where noted, “WT” refers to *snapc1b*^*fh111*/+^ and *snapc1b*^*+/+*^. Bacteria, beads, and dye were injected into the caudal vein and/or hindbrain ventricle.

### Human Alveolar Macrophage Experiments

Alveolar macrophages (AM) were retrieved at bronchoscopy after informed consent and as approved by the Research Ethics Committee of St. James’ Hospital, using a protocol that preserves viability of macrophages from both smokers and nonsmokers (O’Leary et al., 2014). Macrophage migration and microscopical visualization procedures are detailed in Supplemental Experimental Procedures.

### Bacterial Strains

Wild-type *M. marinum* (Mm) (strain M - ATCC #BAA-535) expressing tdTomato under the constitutive promoter *msp12* was used for fluorescence microscopy and quantification of intracellular bacterial burdens (Takaki et al., 2013). The attenuated Δ*erp* mutant Mm was used to enumerate intracellular bacteria (Cosma et al., 2006, Takaki et al., 2013), and WT Mm was used for all other assays. Bacterial were cultures and prepared for injection as described (Takaki et al., 2013).

*M. tuberculosis* H37Ra (ATCC 25177) was used for the human alveolar macrophage studies and prepared as described in Supplemental Experimental Procedures.

### Statistical Analyses

Statistical analyses were performed using Prism 6 (GraphPad). Not significant, p ≥ 0.05, ^∗^p < 0.05; ^∗∗^p < 0.01; ^∗∗∗^p < 0.001; ^∗∗∗∗^p < 0.0001.

## Author Contributions

R.D.B., S.L., C.J.C., J.C., K.T., C.B.M., D.M.T., and L.R. conceived, designed, and analyzed zebrafish experiments; R.D.B., S.L., C.J.C., J.C., K.T., and D.M.T. performed these experiments; S.L., M.P.O., S.M.O., J.K., and L.R. conceived, designed, and analyzed human experiments; M.P.O. and S.M.O. performed these experiments; S.L., R.D.B., and L.R. wrote the paper with input from J.K., D.M.T., M.P.O., S.M.O., and C.B.M.; K.T., and S.L. prepared the figures.

## Accession Numbers

The accession number for the RNA-seq data reported in this paper is GEO: GSE74196.

## Supplemental Information

Document S1. Supplemental Experimental Procedures and Tables S1, S2, and S4

Table S3. Differentially Expressed Genes in snapc1b Mutants, Related to Figure 1

Movie S1. Video Shows a Green Fluorescent Macrophage in the Brain of a Wild-Type and Mutant Zebrafish Larvae at 3 dpf, Related to Figure 2

Movie S2. Video Shows a Forming Granuloma with Green Fluorescent Macrophages and Red Fluorescent Mm in the Brain of a Wild-Type and snapc1b Mutant Zebrafish Larvae at 2 dpi, Related to Figure 3

Document S2. Article plus Supplemental Information
