# Supplementary material for: Lysosomal Disorders Drive Susceptibility to Tuberculosis by Compromising Macrophage Migration
Source: Cell. 2016 Mar 24;165(1):139–52. doi: 10.1016/j.cell.2016.02.034 (PMC4819607; doi:10.1016/j.cell.2016.02.034)
Supplement: Document S1. Supplemental Experimental Procedures and Tables S1, S2, and S4 [file mmc1.pdf]

Cell, Volume *165*

Supplemental Information

Lysosomal Disorders Drive Susceptibility to Tuberculosis by Compromising Macrophage Migration

Russell D. Berg, Steven Levitte, Mary P. O'Sullivan, Seónadh M. O'Leary, C.J. Cambier, James Cameron, Kevin K. Takaki, Cecilia B. Moens, David M. Tobin, Joseph Keane, and Lalita Ramakrishnan

Supplemental Experimental Procedures

Bacterial culture

All Mm strains were grown under hygromycin (Mediatech) selection in Middlebrook's 7H9 medium (Difco) supplemented with glycerol, oleic acid, albumin, dextrose, and Tween-80 (Sigma) (Takaki et al., 2013). Single-cell stocks of Mm were prepared as previously described prior to injection (Takaki et al., 2013). Inocula were determined by microinjection onto 7H10 plates.

Zebrafish husbandry and larval injections

The Tg(*mpeg1:YFP)*^{w200}, Tg(*mpeg1:Brainbow)*^{w201} (expressing tdTomato) and Tg(*lysC:eGFP)*^{nz117} lines were used as previously described (Hall et al., 2007; Pagan et al., 2015; Roca and Ramakrishnan, 2013). All transgenic and *snapc1b(fh111)* lines were maintained as outcrosses to AB.

Bead injections

As a chemoattractant, heat killed bacteria were prepared by incubating a 2x10^4/uL culture of Mm in 7H9.OADS at 80°C for 30min. Sterile blue fluorescent 1 micron beads (Life Technologies F-8814) were mixed with heat killed Mm at a 1:9 ratio of beads:heat-killed Mm and then diluted to 3.64×10^3 beads/nL. Approximately 5 nL of the resulting bead:heat-killed Mm mixture was injected into the hindbrain ventricle of 2 dpf larvae for a total of 1.8×10^4 beads per larva. Images were captured using confocal laser microscopy 24 hours following injection.

Dye and protein injections

LysoTracker Red DND-99 dye (DMSO solution, Life Technologies) was diluted 1:25 in PBS prior to injection of 5 nl into the HBV of 3 dpf larvae. MagicRed-Cathepsin (Immunochemistry Technologies, LLC) was resuspended at the concentration suggested by the manufacturer in DMSO, diluted 1:1 in 1xPBS and injected into the brain of 3 dpf larvae. Dextran-Alexa488 (10,000MW, Molecular Probes) was resuspended in 1xPBS to a concentration of 1 mg/mL, then diluted 1:100 prior to injection into the brain of 3 dpf larvae. CCL2 was injected into the HBV as previously described (Cambier et al., 2014).

Construction of Venus-V2A-*ctsl1* plasmid and *in vitro* transcription

The zebrafish *ctsl1* gene was amplified from cDNA and used to replace *csf1a* in a vector containing pCMV:nlsVenus-V2A (a gift from D. Parichy) by Gibson Assembly (Life Technologies). In vitro transcription was performed with mMessage mMachine SP6 kit (Life Technologies).

Morpholino and RNA injections

All morpholinos used in this work (Table S1) and *in vitro* transcribed *ctsl1* mRNA were diluted in a 1x Tango Buffer (Thermo Scientific), 2% phenol red sodium salt solution (Sigma) and injected 1 nL into the yolk of 1-2 cell stage embryos (Tobin et al., 2012).

Zebrafish mutagenesis, screening and positional cloning

fh111 carriers were identified by infection of gynodiploid larvae (Johnson et al., 1995) with 150-200 CFU of green fluorescent Mm. Putative *fh111* carriers were outcrossed to the wildtype WIK strain and mutants and carriers identified by random crosses between siblings. Bulk segregant analysis (BSA) was performed on pools of mutant progeny and phenotypically wildtype as described (Bahary et al., 2004). Intermediate and fine mapping were conducted using published markers and new markers generated by resequencing of mutant and wild-type progeny within the linked region. Fine mapping determined the causative locus lay between a WIK AseI site at 37.97 mb and a SNP at 38 mb on Chromosome 13. This region contained a single gene, *snapc1b*. Sequencing of all exons and splice junctions within the critical region in mutant embryos and their siblings identified a single splice acceptor site mutation immediately upstream of exon 2, in which the canonical A of the highly conserved AG immediately upstream of the splice acceptor site was converted to GG at position chr13:37996163 (A to G transition). We detected no recombination events at this locus in 854 total meioses from mapping crosses.

Staining

Neutral red staining of 6 dpf larvae was performed as described (Herbomel et al., 2001). Acridine Orange staining was performed by soaking larvae in fish water containing 2 μg/mL acridine orange (ImmunoChemistry, Bloomington, MN) for 30 minutes at 29°C in the dark. Larvae were washed for 5 minutes in fish water at room

temperature and then mounted for microscopy as previously described (Yang et al., 2012).TUNEL assay assay staining of larvae was performed by incubationfor 24 hr in 50 µM of Q-VD-OPh (R&D Systems, Inc.) dissolved in DMSO, after which TUNEL staining was performed as described (Volkman et al., 2004).

Drug treatments

E64d. E64d (Sigma) was dissolved in DMSO and added to fish water to a final concentration of 2-3 µg/mL in 0.5% DMSO. Larvae at 2 dpf were treated with the resulting E64d solution for 24 hours and then transferred to clean fish water for the duration of the experiment.

Q-VD-OPh: Larvae were treated with 50 µM Q-VD-OPh in 0.5% DMSO commencing at 2 dpf with daily water changes.

Genotyping

HRM genotyping: High resolution melt genotyping was conducted using the CFX Connect Real- Time PCR Detection System (BioRad) and the EvaGreen iTag Precision melt supermix. HRM primers were used at final concentration of 0.2 mM with ~10 ng of gDNA in 10 ul reaction. PCR was conducted using Biorad HRM standard cycling parameters.

snapc1b$^{\mathbf{Tg}(\boldsymbol{la010158})}$: The retroviral insertion mutant *snapc1b*$^{Tg(la010158)}$ (ZIRC) (GenBank ID JM495858.1) was genotyped using agarose gel electrophoresis using primers flanking the insertion site and a primer specific for the retroviral insertion (GT186). Wildtype Forward: 5' - GCTGAAATCCATGTCCTTCCA - 3'. GT186 Forward: 5' - GAGTGATTGACTACCCGTCAGCGG - 3'. Common Reverse: 5' - TGCTTTTCTCATGAGCTTCTCT - 3'

snapc1b$^{\boldsymbol{fh111}}$**:** *snapc1b*^{fh111} mutants were genotyped using High Resolution Melt (Tucker and Huynh, 2014). An insertion-deletion polymorphism is present in intron 1-2 in the wildtype AB population. The genomic sequences are

*

Reference 5'- ATTTTTTTCCCTG---C**A**GTGGTAAAC -3'

Fh111 5'- ATTTTTTTCCCTG---C**G**GTGGTAAAC -3'

WT INDEL 5'- ATT----------AAAC**A**GTGGTAAAC -3'

All genotypes are distinguishable with HRM sequencing primers, which bind outside of the polymorphic region: snapc1b_HRM Forward: 5' - CCACAAATTCCAAATGACATTGA -3'. snapc1b_HRM Reverse: 5' - CCTTTTCTCTCTTGGCTCTTGTTT -3'.

Quantitative RT-PCR

Quantitative RT-PCR was performed as previously described (Clay et al., 2007). Total RNA from batches of ~30 embryos per biological replicate was isolated with TRIzol Reagent (Life Technologies) and used to synthesize cDNA with Superscript II reverse transcriptase and oligo dT primers (Invitrogen). Quantitative RT-PCR assays were performed with SYBR green PCR Master Mix (Applied Biosystems) on an ABI Prism 7300 Real Time PCR System (Applied Biosystems). Each biological replicate was run in triplicate, and average values were plotted. Data were normalized to *b-actin* for ΔCt analysis. Primers used for RT-qPCR in this study were: Spliced *snapc1b* Forward: 5'- TGA AAC ACT GCT TGG TCG TC -3'; Spliced *snapc1b* Reverse: 5'- CCC GAC TCT GAT CTG GAA AG -3'; *ctsbb* Forward: 5'- GAG GAA CAG AGG AAC AGA CTT TA-3'; *ctsbb* Reverse: 5'- TGA TCA TCT CAT CAT GTG TGT GA-3'; *ctsl1* Forward: 5'- AGG AAG AGT CAC ACC GTC AG -3'; *ctsl1* Reverse: 5'- GTC ACA TAG CCC TTG TCC CT -3'*; b-actin* Forward 5'- ACC TGA CAG ACT ACC TGA TG -3'; *b-actin* Reverse 5'- TGA AGG TGG TCT CAT GGA TAC -3'; *tnf* Forward 5'- AGG CAA TTT CAC TTC CAA GG -3'; *tnf* Reverse 5'- CAA GCC ACC TGA AGA AAA GG -3'.

RNA-sequencing and analysis

Individual larvae were euthanized at 6 dpf and a small piece of tissue from the head of each animal removed with forceps. The head was placed in HotShot DNA extraction bufferand the torso was placed in 50 μL Trizol for RNA isolation (Invitrogen). gDNA was genotyped by HRM and mutant RNA pooled. Trizol extraction followed as per manufacturer's protocol. RNA was purified with LiCl extraction. Library construction was conducted by the Fred Hutchinson Cancer Research Center Genomics Core and proceeded as described (Miller et al., 2013). Approximately 1 μg RNA was poly-A selected, chemically fragmented and cDNA reverse transcribed using random hexamers. Libraries were prepared using the TruSeq Illumina protocol. Sequencing was performed on an Illumina HiSeq 2000 using 50 bp paired-end reads. RNA sequencing reads were aligned to the zebrafish reference genome

(Zv9.69) using the TopHat/Bowtie aligner (Trapnell et al., 2013). Transcriptome expression analysis was then conducted using the Cufflinks software pipeline (Trapnell et al., 2013). Validation of splicing perturbations in *snapc1b* mutants was assessed using IGV (Thorvaldsdóttir et al., 2013).

Microscopy

Zebrafish: Fluorescence microscopy was performed as previously described (Takaki et al., 2013; Yang et al., 2012). Quantification of bacterial burdens, enumeration of macrophage numbers, and assessments of mycobacterial cording were performed with a Nikon Eclipse Ti-E inverted microscope fitted with 2x, 4x and 10x objectives. Cording was visually assessed as previously described (Clay et al., 2008). For confocal microscopy, larvae were anesthetized in fish water containing 0.025% Tricaine and embedded in 1.5% low melting point agarose on optical bottom plates (MatTek Corporation). A Nikon A1 confocal microscope with a 20x Plan Apo 0.75 NA objective was used to generate 40-120 μm z-stacks with 1–2 μm step size. The galvano scanner was used for all static imaging, and the resonant scanner was used for time-lapse imaging. Time-lapse images were taken at 3-15 minute intervals for 5-20 hours. Data were acquired with NIS Elements Version 4.3 (Nikon). Macrophage tracks were generated and volume rendering performed using Imaris 7.7-8.0 (Bitplane Scientific Software). Movies were exported from Imaris to iMovie (Apple Corporation) for addition of labels and stitching. All microscope scoring of staining, bacterial morphology and macrophage morphology was performed blinded.

Human: Adherence purified AM were fixed with 2% paraformaldehyde, stained with Hoechst 33342 (10 μg/ml) and analyzed using a Zeiss LSM 510 laser confocal microscope (Carl Zeiss Microscopy GmbH, Oberkochen, Germany) to detect autofluorescence. Phase contrast images were collected with a transmitted light detector. Images were generated and viewed using Zen software (Carl Zeiss Microscopy).

Human alveolar macrophage migration assay

Human alveolar macrophages: Non-smokers were defined as those subjects reporting no smoking history, ex-smokers were defined as those having ceased smoking more than 6 months prior to sampling and smokers were defined as those currently smoking. Bronchial washing fluid was filtered through a 100 μm nylon strainer (BD Falcon, BD Bioscience, Belgium) and centrifuged at 390 g for 10 min. AM were resuspended in RPMI

1640 culture media supplemented with 0.1% heat inactivated fetal bovine serum (FBS, Gibco), 50 U/ml amphotericin B and 50 µg/ml cefotaxime. For characterization of initial cell population, 0.5 x 10^5 cells were seeded on an 8 chamber Lab-teks® (Thermo Fisher Scientific), fixed with ice cold methanol containing 10 µg/ml of Hoechst 33258 (Sigma Aldrich) for 4 minutes.

Mycobacteria: *M. tuberculosis* H37Ra was obtained from the American Type Culture Collection (ATCC 25177, Manassas, VA) and prepared as previously described (Ryan et al, 2011) and resuspended at 1 x 10^8 CFU/ml in RPMI 1640 with 0.1% heat inactivated fetal bovine serum (RPMI/0.1% hiFBS).

Migration assay: The migration assay was carried out in transwell chambers consisting of 8 µm pore polycarbonate cell culture inserts in a 24-well companion plate (Corning Costar™, Nijmegen, Netherlands) (Opalek et al., 2007). Experiments were performed in triplicate unless otherwise stated. Mtb, Tuberculin PPD (30µg/ml) (Statens Serum Institut, Denmark) diluted in RPMI/0.1% hiFBS, or RPMI/0.1% hiFBS alone or RPMI/10% zymosan-activated serum (ZAS) were placed in the lower chambers. AM (0.5 x 10^5 cells) were added to the upper chambers and plates were incubated at 37°C, 5% CO_2 for 2 hrs unless otherwise stated. Migrated cells were fixed in ice-cold methanol containing 10 µg/ml of Hoechst 33258 (Sigma Aldrich) for 4 minutes. AM were counted by fluorescent microscopy (Olympus IX51, Olympus Europa GmbH, Germany) to establish the number of migrating cells and the percentage vacuolated AM in the initial and migrating cell populations.

Neutral red staining of lysosomes: Adherence-purified AM cultured in 24 well plates were stained by adding neutral red (Sigma) (40 µg/ml) and incubating for 30 min at 37°C, 5% CO_2 (Repetto et al., 2008). Images were obtained using an Olympus IX51 microscope with a DP71 CCD camera and cellSens software.

Table S1. Morpholinos used in this study, related to Experimental Procedures.

Gene[a]	Used name[b]	Morpholino sequence	MO target	MO concentration (mM)	Reference
snapc1b	snapc1b	CTTGTTTACCACTGCAGGGAAA	i1/e2	0.05	This work
snapc1b	snapc1b	ACCACTTCCCTGAAATGCTCCA	atg/5'UTR	0.5	This work
ctsl.1	ctsl1	GTTTGTTTTAATTTCCTACCATC	e3i3	1.3	This work
ctsbb	ctsbb	ACACTGCAGATGGAGAAAACA	i3e4	0.05	This work
ctsbb	ctsbb atg	GCGAACACACACACACGCCAC	atg/5'UTR	0.05	This work
tnfr1	tnfr1	CTGCATTGTGACTTACTTATCG	E5/i6	0.32	(Roca & Ramakrishnan, 2013)
hexa	hexa	ACATTGCCAGGAAGTACTCAC	e2/i2	1	This work
gba	gba	CGTCTGATTATTGTTACACACC	e1/i1	0.5	This work
arsa	arsa	TATGTTTGACCTGGCTTTACCT	e1/i1	1	This work

[a]Gene abbreviations follow the "Zebrafish Nomenclature Guidelines" (https://wiki.zfin.org/display/general/ZFIN+Zebrafish+Nomenclature+Guidelines)

[b]Name, often simplified, that was used in this paper

Table S2. Selected differentially expressed genes in *snapc1b* mutants, related to Figure 1.

Category	Gene	*snapc1b* +/+ FPKM	*snapc1b* -/- FPKM	Fold change (mutant/wt)	Associated LSD
Myelopoeisis	csf1rb	1.07	0.32	0.29	
	csf3r	0.85	0.71	0.84	
	csf1a	1.78	1.85	1.04	
	csf1ra	5.24	5.86	1.12	
	csf2rb	0.34	0.46	1.37	
	csf1b	2.60	3.75	1.44	
	spi1b	2.57	2.47	0.96	
	spi1a	1.63	2.46	1.51	
	irf8	0.89	1.99	2.23	
	mpeg1 (2 of 3)	11.05	32.07	2.90	
	mpeg1	3.12	4.82	1.54	
	gata1a	1.78	1.24	0.69	
	gata1b	0.09	0.03	0.35	
	gata2a	4.31	3.49	0.81	
	gata2b	0.49	0.40	0.80	
	gata3	20.87	15.79	0.76	
	gata4	3.26	2.70	0.83	
	gata5	5.73	7.57	1.32	
	gata6	9.49	10.00	1.05	
	cebp1	0.41	0.29	0.71	
	cebpa	16.82	17.12	1.02	
	cebpb	24.19	44.02	1.82	
	cebpd	116.14	137.37	1.18	
	cebpg	31.68	29.80	0.94	
Inflammation	atf3	8.93	15.99	1.79	
	cebpb	24.19	44.02	1.82	
	elf3	15.67	28.85	1.84	
	junbb	16.86	38.72	2.30	
	junba	11.23	36.13	3.22	
	ptgs2a	6.72	10.64	1.58	
	rgs4	22.31	45.92	2.06	
	ptgs2b	2.62	5.71	2.18	
	ncf1	2.48	5.44	2.20	
	plek	1.23	118.44	96.27	
	m17	0.04	0.21	5.10	
	il1b	0.89	9.07	10.24	
	mmp13a	2.23	10.09	4.52	
	mmp9	3.89	23.70	6.09	

	irg1	0.46	3.62	7.93	
	irg1l	9.42	91.73	9.74	
	tnfa	0.034	0.18	2.44	
Lysosomal	npc1	15.07	20.63	1.37	
	npc2	59.17	63.20	1.07	
	smpd1	7.82	9.84	1.26	
	hexb	26.29	25.22	0.96	
	hexa	8.24	6.58	0.80	Tay-Sachs
	arsa	3.48	2.59	0.75	Metachromatic leukodystrophy
	arsb	6.71	7.20	1.07	
	gba	4.82	5.00	1.04	Gaucher’s
	gla	0.52	0.42	0.80	
	ctsa	23.99	24.66	1.03	
	ctsba	72.88	96.18	1.32	
	**ctsbb**	**57.38**	**5.18**	**0.09**	
	ctsc	25.25	24.17	0.96	
	ctsd	98.61	134.24	1.36	
	ctsf	66.41	60.22	0.91	
	ctsh	44.06	22.82	0.52	
	ctsk	14.80	19.61	1.33	
	ctskl	5.20	3.85	0.74	
	**ctsl.1**	**378.02**	**47.93**	**0.13**	
	ctsl1a	305.85	655.09	2.14	
	galns	2.57	2.56	0.99	
	gnsa	10.79	10.74	1.00	
	gnsb	13.39	10.69	0.80	
	naglu	1.03	1.16	1.13	
	neu1	15.07	20.63	1.37	
	psap	59.17	63.20	1.07	
	scpep1	7.82	9.84	1.26	
	sgsh	26.29	25.22	0.96	
	idua	8.24	6.58	0.80	
	tpp1	3.48	2.59	0.75	
	tmem55a	6.71	7.20	1.07	
	tmem55b(1 of 2)	4.82	5.00	1.04	
	tmem55b (2 of 2)	0.52	0.42	0.80	
	lamp1	23.99	24.66	1.03	
	lamp2	71.91	86.35	1.20	
	atp6v1h	29.75	30.88	1.04	
	clcn7	5.19	6.04	1.16	
	**FPKM = Fragments per kilobase mapped**				

Table S4. Characteristics and migration of alveolar macrophages retrieved by bronchoalveolar lavage from non-smokers, ex-smokers and smokers, related to Figure 7.

				**Initial AM population**		**Migrated AM to Mtb H37Ra**	
**Sample**	**Age years (mean ± SEM)**	**Smoking status**	**Pack years (years ceased)**	**# cells counted**	**% vacuolar cells (mean ± SEM)**	**# cells counted**	**% vacuolar cells (mean ± SEM)**
SJH209		non-smoker	-	320	2.81	405[a]	non-detectable
SJH222		non-smoker	-	441	1.36	-	-
SJH223		non-smoker	-	580	0.00	-	-
SJH226		non-smoker	-	479	0.42	-	-
	**52.75 ± 4.27**				**1.15 ± 0.62**		
SJH210		ex-smoker	40 (6)	107	9.35	288[ab] (284[c])	non-detectable
SJH211		ex-smoker	27 (16)	201	4.48	152[ab] (144[c])	non-detectable
SJH212		ex-smoker	(0.5)	418	8.61	338	non-detectable
SJH219		ex-smoker	54 (34)	439	9.57	-	-
	**55.25 ± 9.74**				**8.00 ± 1.19**		
SJH214		smoker	56	441	52.15	203	2.96
SJH215		smoker	100	398	60.80	40	30.00
SJH216		smoker	20	429	74.36	41	21.95
SJH220		smoker	5.7	412	69.66	250	19.20
SJH227		smoker	52.5	424	81.37	68	32.35
SJH218		smoker	47	531	68.17	-	-
	**65 ± 3.04**				**67.75 ± 4.18**		**21.29 ± 5.19**

[a] Experiments not performed in triplicate [b] migrated cells at 1.5hr timepoint [c] migrated cells at 3hr timepoint

Supplemental References

Bahary, N., Davidson, A., Ransom, D., Shepard, J., Stern, H., Trede, N., Zhou, Y., Barut, B., and Zon, L.I. (2004). The Zon laboratory guide to positional cloning in zebrafish. Methods Cell Biol *77*, 305-329.

Cambier, C.J., Takaki, K.K., Larson, R.P., Hernandez, R.E., Tobin, D.M., Urdahl, K.B., Cosma, C.L., and Ramakrishnan, L. (2014). Mycobacteria manipulate macrophage recruitment through coordinated use of membrane lipids. Nature *505*, 218-222.

Clay, H., Davis, J.M., Beery, D., Huttenlocher, A., Lyons, S.E., and Ramakrishnan, L. (2007). Dichotomous role of the macrophage in early Mycobacterium marinum infection of the zebrafish. Cell Host Microbe *2*, 29-39.

Clay, H., Volkman, H.E., and Ramakrishnan, L. (2008). Tumor necrosis factor signaling mediates resistance to mycobacteria by inhibiting bacterial growth and macrophage death. Immunity *29*, 283-294.

Cosma, C.L., Swaim, L.E., Volkman, H., Ramakrishnan, L., and Davis, J.M. (2006). Zebrafish and frog models of Mycobacterium marinum infection. Curr Protoc Microbiol *Chapter 10*, Unit 10B.12.

Hall, C., Flores, M.V., Storm, T., Crosier, K., and Crosier, P. (2007). The zebrafish lysozyme C promoter drives myeloid-specific expression in transgenic fish. BMC Dev Biol *7*, 42.

Herbomel, P., Thisse, B., and Thisse, C. (2001). Zebrafish Early Macrophages Colonize Cephalic Mesenchyme and Developing Brain, Retina, and Epidermis through a M-CSF Receptor-Dependent Invasive Process. Developmental Biology *238*, 274-288.

Johnson, S.L., Africa, D., Horne, S., and Postlethwait, J.H. (1995). Half-tetrad analysis in zebrafish: mapping the ros mutation and the centromere of linkage group I. Genetics *139*, 1727-1735.

Miller, A.C., Obholzer, N.D., Shah, A.N., Megason, S.G., and Moens, C.B. (2013). RNA-seq-based mapping and candidate identification of mutations from forward genetic screens. Genome Res *23*, 679-686.

Opalek, J.M., Ali, N.A., Lobb, J.M., Hunter, M.G., and Marsh, C.B. (2007). Alveolar macrophages lack CCR2 expression and do not migrate to CCL2. Journal of inflammation *4*, 19.

Pagan, A.J., Yang, C.T., Cameron, J., Swaim, L.E., Ellett, F., Lieschke, G.J., and Ramakrishnan, L. (2015). Myeloid Growth Factors Promote Resistance to Mycobacterial Infection by Curtailing Granuloma Necrosis through Macrophage Replenishment. Cell Host Microbe *18*, 15-26.

Repetto, G., del Peso, A., and Zurita, J.L. (2008). Neutral red uptake assay for the estimation of cell viability/cytotoxicity. Nat Protoc *3*, 1125-1131.

Roca, F.J., and Ramakrishnan, L. (2013). TNF dually mediates resistance and susceptibility to mycobacteria via mitochondrial reactive oxygen species. Cell *153*, 521-534.

Takaki, K., Davis, J.M., Winglee, K., and Ramakrishnan, L. (2013). Evaluation of the pathogenesis and treatment of Mycobacterium marinum infection in zebrafish. Nat Protoc *8*, 1114-1124.

Thorvaldsdóttir, H., Robinson, J.T., and Mesirov, J.P. (2013). Integrative Genomics Viewer (IGV): high-performance genomics data visualization and exploration. Brief Bioinform *14*, 178-192.

Tobin, D., Roca, F., Oh, S., McFarland, R., Vickery, T., Ray, J., Ko, D., Zou, Y., Bang, N., CHau, T., *et al.* (2012). Host genotype-specific therapies can optimize the inflammatory response to mycobacterial infections. Cell *148*, 434-446.

Trapnell, C., Hendrickson, D.G., Sauvageau, M., Goff, L., Rinn, J.L., and Pachter, L. (2013). Differential analysis of gene regulation at transcript resolution with RNA-seq. Nat Biotechnol *31*, 46-53.

Tucker, E.J., and Huynh, B.L. (2014). Genotyping by high-resolution melting analysis. Methods Mol Biol *1145*, 59-66.

Volkman, H.E., Clay, H., Beery, D., Chang, J.C., Sherman, D.R., and Ramakrishnan, L. (2004). Tuberculous granuloma formation is enhanced by a mycobacterium virulence determinant. PLoS Biol *2*, e367.

Yang, C.T., Cambier, C.J., Davis, J.M., Hall, C.J., Crosier, P.S., and Ramakrishnan, L. (2012). Neutrophils exert protection in the early tuberculous granuloma by oxidative killing of mycobacteria phagocytosed from infected macrophages. Cell Host Microbe *12*, 301-312.
