# Supplementary figures and images for: Lysosomal Disorders Drive Susceptibility to Tuberculosis by Compromising Macrophage Migration
Source: Cell. 2016 Mar 24;165(1):139–52. doi: 10.1016/j.cell.2016.02.034 (PMC4819607; doi:10.1016/j.cell.2016.02.034)

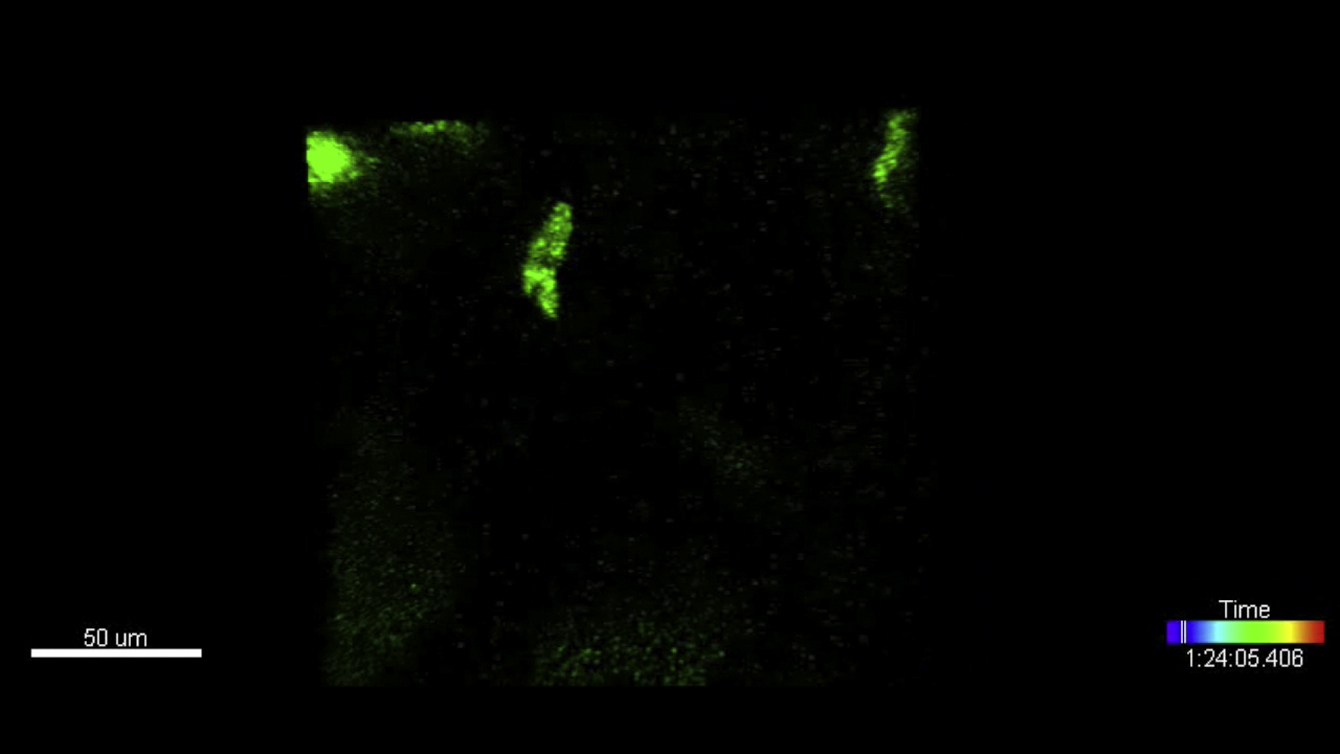

Supplement: Movie S1. Video Shows a Green Fluorescent Macrophage in the Brain of a Wild-Type and Mutant Zebrafish Larvae at 3 dpf, Related to Figure 2 [file mmc3.jpg]

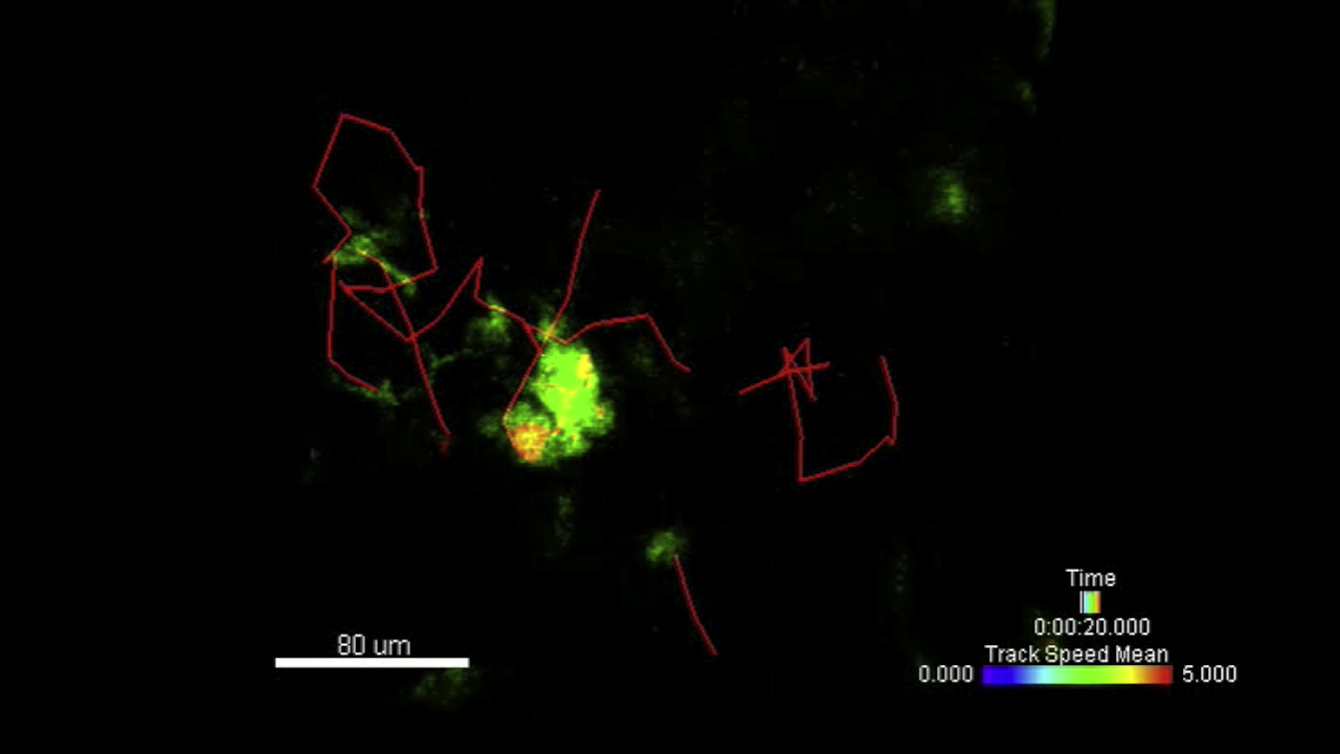

Supplement: Movie S2. Video Shows a Forming Granuloma with Green Fluorescent Macrophages and Red Fluorescent Mm in the Brain of a Wild-Type and snapc1b Mutant Zebrafish Larvae at 2 dpi, Related to Figure 3 [file mmc4.jpg]
