# Supplementary material for: Lysosomal Disorders Drive Susceptibility to Tuberculosis by Compromising Macrophage Migration
Source: Cell. 2016 Mar 24;165(1):139–52. doi: 10.1016/j.cell.2016.02.034 (PMC4819607; doi:10.1016/j.cell.2016.02.034)
Supplement: Document S2. Article plus Supplemental Information [file mmc5.pdf]

Cell

Article

Lysosomal Disorders Drive Susceptibility to Tuberculosis by Compromising Macrophage Migration

Graphical Abstract

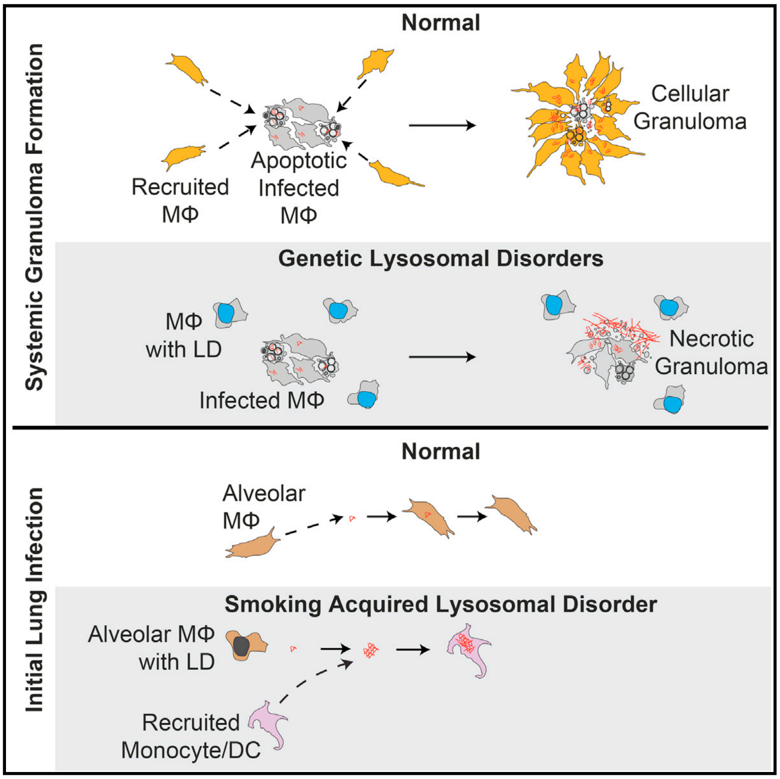

Authors

Russell D. Berg, Steven Levitte, Mary P. O'Sullivan, ..., David M. Tobin, Joseph Keane, Lalita Ramakrishnan

Correspondence

josephmk@tcd.ie (J.K.), lr404@hermes.cam.ac.uk (L.R.)

In Brief

Lysosomal dysfunction caused by genetic mutations or accumulation of tobacco particulates in the lysosomal compartment impairs macrophage function and formation of stable tuberculous granulomas, providing a possible explanation for the increased susceptibility to tuberculosis in smokers.

Highlights

- Lysosomal storage diseases reduce macrophage endocytic recycling and migration
- Reduced macrophage migration increases tuberculosis severity via granuloma breakdown
- Tobacco smoke particles accumulate in lysosomes of smokers' alveolar macrophages
- Lysosomal particles reduce smokers' macrophage migration to infecting mycobacteria

Accession Numbers

GSE74196

CrossMark
Berg et al., 2016, Cell *165*, 139–152
March 24, 2016 ©2016 The Authors
http://dx.doi.org/10.1016/j.cell.2016.02.034

CellPress

Cell

Lysosomal Disorders Drive Susceptibility to Tuberculosis by Compromising Macrophage Migration

Russell D. Berg,[1,9] Steven Levitte,[1,2,9] Mary P. O'Sullivan,[3] Seónadh M. O'Leary,[3] C.J. Cambier,[4,10] James Cameron,[5,11] Kevin K. Takaki,[2] Cecilia B. Moens,[6] David M. Tobin,[7,8] Joseph Keane,[3,*] and Lalita Ramakrishnan[2,5,*]
[1]Molecular & Cellular Biology Graduate Program and Medical Scientist Training Program, University of Washington, Seattle, WA 98195, USA
[2]Department of Medicine, University of Cambridge, MRC Laboratory of Molecular Biology, Cambridge CB2 0QH, UK
[3]Department of Clinical Medicine, Institute of Molecular Medicine, Trinity College Dublin, Dublin 8, Ireland
[4]Immunology Graduate Program, University of Washington, Seattle, WA 98195, USA
[5]Department of Microbiology, University of Washington, Seattle, WA 98195, USA
[6]Fred Hutchinson Cancer Research Center, Seattle, WA 98109, USA
[7]Department of Molecular Genetics and Microbiology, Duke University, Durham, NC 27710, USA
[8]Department of Immunology, Duke University, Durham, NC 27710, USA
[9]Co-first author
[10]Present address: Department of Chemistry, Stanford University, Stanford, CA 94305, USA
[11]Present address: Environmental and Fisheries Science Division, National Oceanic and Atmospheric Administration, Seattle, WA 98115, USA
*Correspondence: josephmk@tcd.ie (J.K.), lr404@hermes.cam.ac.uk (L.R.)
http://dx.doi.org/10.1016/j.cell.2016.02.034

SUMMARY

A zebrafish genetic screen for determinants of susceptibility to *Mycobacterium marinum* identified a hypersusceptible mutant deficient in lysosomal cysteine cathepsins that manifests hallmarks of human lysosomal storage diseases. Under homeostatic conditions, mutant macrophages accumulate undigested lysosomal material, which disrupts endocytic recycling and impairs their migration to, and thus engulfment of, dying cells. This causes a buildup of unengulfed cell debris. During mycobacterial infection, macrophages with lysosomal storage cannot migrate toward infected macrophages undergoing apoptosis in the tuberculous granuloma. The unengulfed apoptotic macrophages undergo secondary necrosis, causing granuloma breakdown and increased mycobacterial growth. Macrophage lysosomal storage similarly impairs migration to newly infecting mycobacteria. This phenotype is recapitulated in human smokers, who are at increased risk for tuberculosis. A majority of their alveolar macrophages exhibit lysosomal accumulations of tobacco smoke particulates and do not migrate to *Mycobacterium tuberculosis.* The incapacitation of highly microbicidal first-responding macrophages may contribute to smokers' susceptibility to tuberculosis.

INTRODUCTION

Tuberculosis (TB) involves a series of interactions between macrophages and the infecting mycobacterium with this proposed sequence of events (Cambier et al., 2014a; Srivastava et al., 2014): inhaled mycobacteria are engulfed by lung alveolar macrophages and, if not cleared during this initial interaction, are transported deeper into the lung. Here, newly recruited myeloid and other immune cells aggregate around the infected cells to form organized granulomas.

The study of zebrafish infected with *M. marinum* has enabled the dissection of these steps of TB pathogenesis, aided by the genetic tractability of this model organism and its optical transparency during its first few weeks of life (Cambier et al., 2014a). Newly infecting bacteria can be transported across epithelial barriers by permissive macrophages (Cambier et al., 2014b). Additional macrophages are recruited to the initial infected macrophage to form the tuberculous granuloma (Cambier et al., 2014a). Cellular expansion of the granuloma, and intracellular bacterial growth within it, proceeds through apoptosis of the infected macrophages and their phagocytosis by newly arriving uninfected macrophages (Davis and Ramakrishnan, 2009). On the one hand, bacterially mediated granuloma expansion can promote infection through bacterial spread into newly recruited macrophages (Davis and Ramakrishnan, 2009). On the other hand, if the supply of uninfected macrophages is limiting, apoptotic infected cells in the granuloma undergo secondary necrosis, causing granuloma breakdown and the release of bacteria into the extracellular space, which enables their accelerated growth (Pagán et al., 2015).

In this work, we characterize a zebrafish mutant identified in a forward genetic screen (Tobin et al., 2010) to reveal how, during genetic lysosomal storage disorders, the accumulation of undegraded products in the macrophage lysosome impairs the migration of these phagocytic cells. The disruption of macrophage migration contributes to the pathogenesis of the lysosomal storage disease in the uninfected state and causes granuloma breakdown during tuberculous infection, which underlies hypersusceptibility. The mutation maps to *snapc1b*,

This is an open access article under the CC BY license (http://creativecommons.org/licenses/by/4.0/).

a transcriptional co-regulator that causes lysosomal storage through reduced expression of lysosomal cysteine cathepsins B and L. Using zebrafish models of human lysosomal storage diseases, we generalize our findings to show that the accumulation of diverse biological substrates, as well as inert particles, compromises macrophage migration through the derangement of endocytic recycling. We then show that lysosomal storage in macrophages inhibits their migration to engulf newly infecting bacteria. Because the resident alveolar macrophages of human cigarette smokers have been reported to accumulate particulate material, we asked whether smokers' macrophages are similarly compromised in their response to mycobacterial infection. We find that the majority of smokers' alveolar macrophages have enlarged lysosomes filled with opaque material and are impaired in their migration to *M. tuberculosis*. The compromised migration of these highly microbicidal first responding macrophages suggests a mechanistic explanation for the reported association between smoking and TB (Lin et al., 2007).

RESULTS

snapc1b Zebrafish Mutant Hypersusceptibility to *M. marinum* Infection Is Characterized by Granuloma Breakdown

The zebrafish mutant *fh111*, identified in a forward genetic screen (Tobin et al., 2010), was hypersusceptible to *M. marinum*, displaying increased bacterial growth relative to wild-type siblings after intravenous infection (Figures 1A and 1B). *fh111* infection was characterized by the breakdown of forming granulomas accompanied by bacterial cording, a characteristic morphology acquired by rapidly growing extracellular bacteria after release from necrotic macrophages (Pagán et al., 2015; Tobin et al., 2010) (Figure 1C). We used bacterial cording as a sensitive and specific phenotype to map *fh111* (Figure 1D) (Tobin et al., 2010). *fh111* maps to a splice acceptor site mutation in the exon 1–2 junction of the zebrafish *snapc1b* gene on chromosome 13 (Figure S1A), one of two orthologs of human *SNAPC1* (Small Nuclear RNA Activating Complex Polypeptide 1) that encodes a component of the basal transcriptional machinery for RNA Pol II and III-dependent transcription (Henry et al., 1998). Zebrafish *snapc1b* has higher amino acid identity to human *SNAPC1* than its paralog *snapc1a*, located on chromosome 20 (39% versus 35%, respectively) (Flicek et al., 2014). RNA sequencing (RNA-seq) analysis of wild-type (WT) animals at 6 days post-fertilization (dpf) showed that *snapc1b* RNAs were ~35-fold more abundant than *snapc1a* RNAs (GEO: GSE74196). We confirmed the location and transcriptional consequence of *fh111* by RNA-seq and qRT-PCR (Figures S1A and S1B). Causality of the *fh111* mutation was confirmed by a splice-blocking antisense oligonucleotide (morpholino) that targeted the same exon 1–2 splice junction of *snapc1b* (Table S1) that phenocopied *fh111* susceptibility (Figures S1C and S1D) and by non-complementation with an independent retroviral insertion allele that disrupts exon 1 of *snapc1b* (*la010158*) (Figures S1E and S1F) (Varshney et al., 2013).

In sum, our findings suggest that Snapc1b deficiency causes hypersusceptibility to mycobacterial infection through early granuloma breakdown, which releases mycobacteria into the extracellular milieu that is more growth permissive than the intracellular environment, culminating in bacterial cording morphology (Pagán et al., 2015).

Macrophages of *snapc1b* Mutants Are Increased in Number and Have Enlarged Lysosomes

Granuloma breakdown can result from a global reduction in macrophage numbers available to replenish the granuloma (Pagán et al., 2015). We were surprised to find that, even in uninfected *snapc1b* mutants, macrophage numbers were increased as revealed by increased numbers of fluorescent macrophages in transgenic animals (Ellett et al., 2011) and by staining with neutral red, a vital dye that accumulates in macrophages (Davis and Ramakrishnan, 2009) (Figures 1E–1G). The increased abundance of microglia, tissue resident macrophages of the brain derived from a primitive hematopoietic lineage (Clements and Traver, 2013), suggested a derangement in multiple waves of myelopoiesis (Figures 1H and 1I).

Most of the macrophages in mutants were enlarged and discoid in shape (Figures 1J, 1K, and S2A–S2C). The enlarged macrophages contained prominent vacuoles, which were revealed to be fused lysosomes by staining with LysoTracker (Figure 1L) (Peri and Nüsslein-Volhard, 2008). The mutant lysosomes were larger than wild-type and occupied a greater proportion of total macrophage volume (Figure 1M). This aberrant macrophage morphology is similar to what has been observed in human lysosomal storage disorders (Kieseier et al., 1997). Our observation of an increased abundance of tissue resident macrophages (histiocytosis) accompanied by increased expression of myeloid growth factors (Table S2) also mirrors findings in several human lysosomal storage disorders (Allen, 2008; Hsu et al., 2012). Neutrophils, the other myeloid cells present at this developmental stage, were not increased in *snapc1b* mutants and displayed normal morphology, consistent with their lack of involvement in homeostatic scavenger functions (Figure S2D; data not shown).

snapc1b Mutant Macrophages Become Vacuolated and Immotile as a Result of Lysosomal Accumulation of Undigested Cell Debris

To understand the development of vacuolated morphology, we compared microglial morphology and dynamics in uninfected animals during physiological efferocytosis of apoptotic neurons. In wild-type animals, macrophages assumed a vacuolated morphology upon phagocytosis of particulate material (presumably cell debris) but reverted to normal within 4 hr (Figure 2A; Movie S1). Vacuolated morphology was accompanied by a transient reduction in speed of homeostatic migration; movement resumed upon reversion to normal morphology (Figures 2A and 2B; Movie S1). In *snapc1b* mutants, macrophages became irreversibly vacuolated after only a few phagocytic events, accompanied by sustained immotility and consequently reduced displacement—36 μm versus 131 μm for wild-type—over a 13-hr observation period (Figures 2A and 2B; Movie S1). These vacuolated macrophages did not have obvious phagocytic defects and continued to extend pseudopods in apparent phagocytic events (Figure 2A; Movie S1). Vacuolated macrophages were also unable to migrate in response to the chemotactic molecule

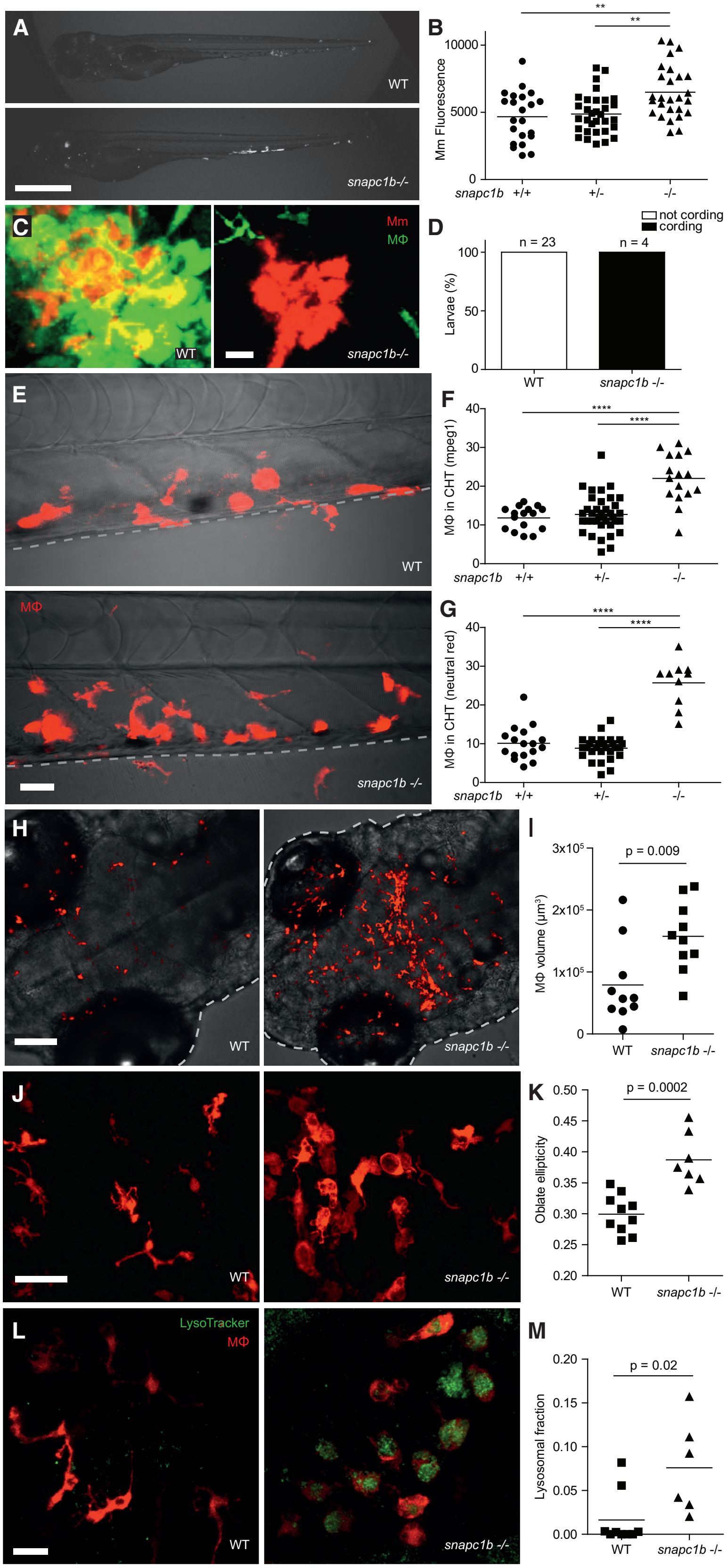

Figure 1. *snapc1b* Mutants Are Hypersusceptible to *M. marinum* and Have Increased Numbers of Macrophages that Display Vacuolated Morphology

(A) Representative images of wild-type (WT) and *snapc1b*$^{fh111/fh111}$ mutant larvae 4 days post-infection (dpi) with 150 *M. marinum* (Mm). Scale bar, 300 μm.

(B) Quantification of Mm burden measured by fluorescence in *snapc1b*$^{fh111/+}$ incross larvae at 5 dpi with 240 Mm.

(C) Confocal images of green fluorescent macrophages (MΦ) and red fluorescent bacteria in intact granulomas of WT larvae and extracellular corded bacteria following complete granuloma breakdown in *snapc1b* mutant larva at 2 dpi with 200 Mm. Scale bar, 15 μm.

(D) Quantification of bacterial cording in larvae from an incross of *snapc1b*$^{fh111/+}$ parents at 5 dpi with 200 Mm.

(E) Confocal images of the caudal hematopoietic tissue (CHT) of representative WT and *snapc1b* mutant larvae with red fluorescent macrophages at 6 days post-fertilization (dpf). Scale bar, 20 μm.

(F and G) Quantification of fluorescent macrophages (F) and neutral red-stained cells (G) in the CHT of *snapc1b*$^{fh111/+}$ incross larvae at 6 dpf.

(H) Confocal images of fluorescent macrophages in the head of representative WT and *snapc1b* mutant larvae at 3 dpf. Dotted lines indicate the outline of larvae. Scale bar, 100 μm.

(I) Total macrophage volume in the brains of WT and *snapc1b* mutant larvae at 5 dpf. Volumetric analysis performed from 3D confocal images on red fluorescence signal.

(J) Confocal images of fluorescent macrophages in the brain of WT and *snapc1b* mutant larvae at 3 dpf. Scale bar, 60 μm.

(K) Measurement of oblate ellipticity of macrophages in the brains of WT and *snapc1b* mutant larvae at 3 dpf.

(L) Confocal images red fluorescent macrophages stained with LysoTracker green in the brains of 3 dpf WT and *snapc1b* mutant larvae. Scale bar, 30 μm.

(M) Average lysosomal volume per animal normalized to total macrophage volume. Macrophage and lysosomal volumes were determined by volumetric analysis of red fluorescence (macrophages) and green fluorescence (lysosomes) in 3D confocal images.

Statistical significance was assessed by one-way ANOVA with Sidak's post-test (B, F, and G) or Student's t test (I, K, and M). See also Figures S1 and S2, and Tables S2 and S3.

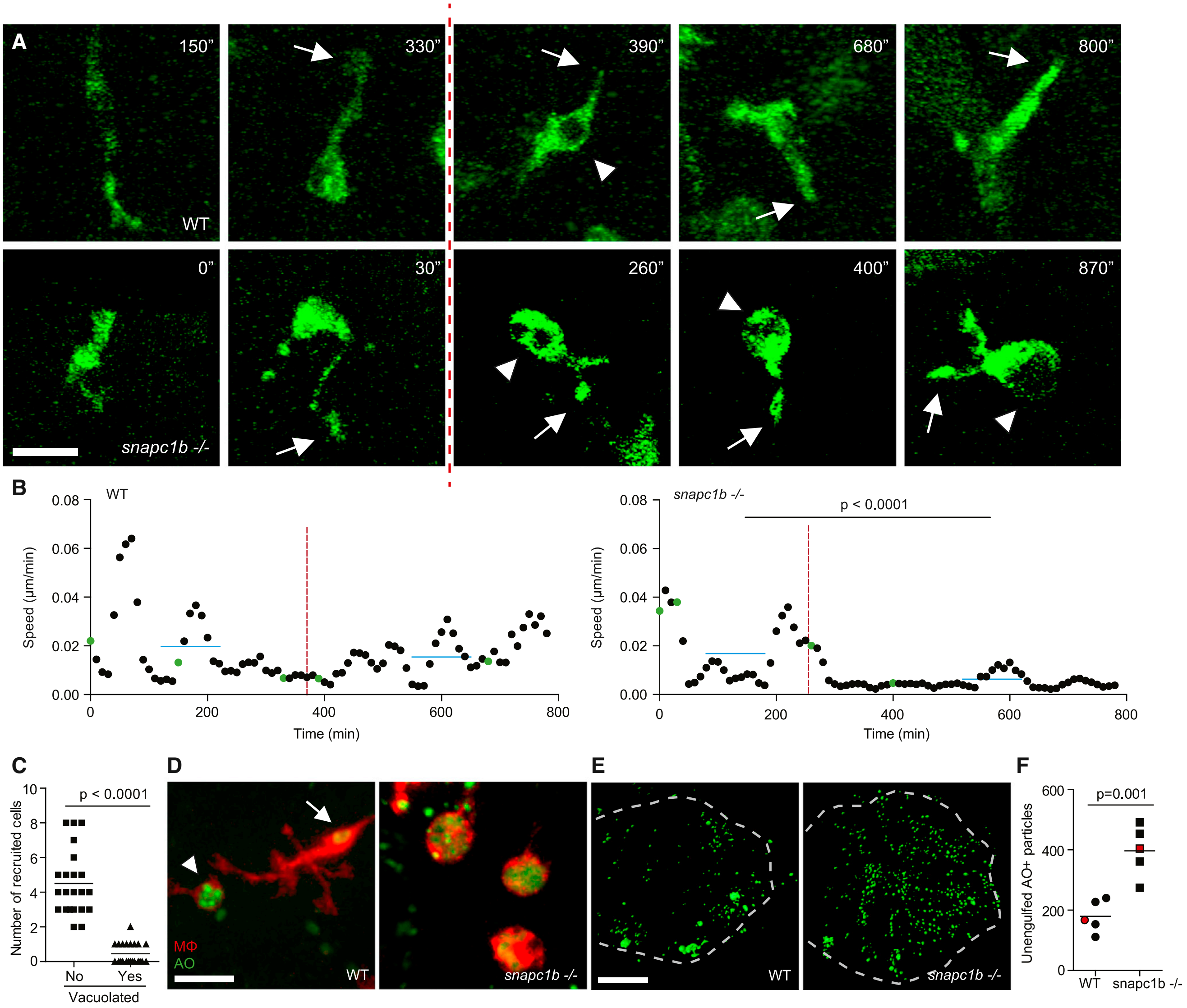

Figure 2. Lysosomal Storage in *snapc1b* Mutants Compromises Physiological Efferocytosis

(A) Still images from confocal video of green fluorescent macrophages in *snapc1b* mutant larval and WT sibling brains. Time of image is indicated in minutes. Arrows mark pseudopodia; arrowheads mark vacuoles. Vertical dotted red line indicates the time point immediately following phagocytic event. Scale bar, 15 μm.

(B) Speed of WT and *snapc1b* mutant macrophages from the confocal video in (A). Average speed before and after the phagocytic events are indicated by a horizontal blue line. Green dots correspond to time points in the images shown in (A).

(C) Migration of normal and vacuolated macrophages from the same animal to CCL2 injected into the HBV.

(D) Representative confocal image of red fluorescent macrophages stained with acridine orange (AO) in brains of *snapc1b* mutant larvae and WT siblings at 3 dpf. Arrow marks a wild-type macrophage with very little AO staining. Arrowhead marks a rare AO positive macrophages seen in WT brains. Scale bar, 30 μm.

(E and F) Confocal images (E) and quantification (F) of green fluorescent acridine-orange-stained unengulfed cell debris in the brains of *snapc1b* mutant larvae and WT siblings at 5 dpf. Scale bar, 150 μm. Images in (E) denoted as red data points in (F).

Statistical significance was assessed by Student’s t test (B and F) and paired t test (C). See also Figure S3.

CCL2 injected into the hindbrain ventricle (HBV) (Cambier et al., 2014b), indicating a broad migratory defect toward both cell debris and chemotactic factors (Figure 2C). These findings are consistent with observations that macrophages from patients with Gaucher’s disease, the most common human lysosomal storage disorder, are defective for migration but competent for phagocytosis (Aflaki et al., 2014).

Acridine orange staining confirmed that the lysosomal accumulations in mutant macrophages were phagocytosed apoptotic bodies (Abrams et al., 1993; Peri and Nüsslein-Volhard, 2008) (Figure 2D). Together, these findings suggested that lysosomal accumulation of undigested cell debris in mutant macrophages impairs migration irreversibly and leads to progressive macrophage incapacitation. If so, then we

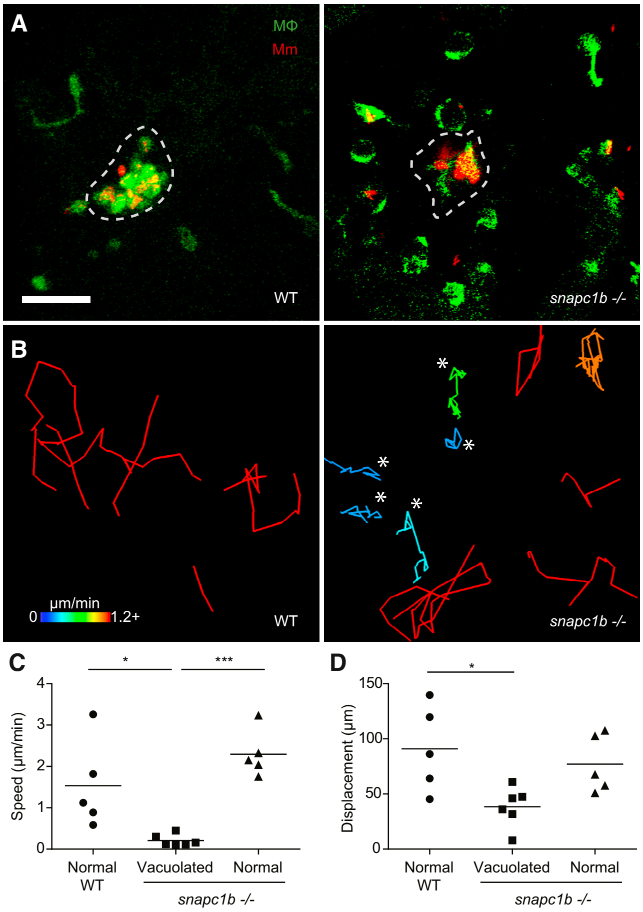

Figure 3. *snapc1b* Mutant Macrophages Fail to Participate in Granuloma Formation

(A) Confocal images of granulomas in the hindbrain ventricle of *snapc1b* mutant larvae and WT siblings with green fluorescent macrophages at 2 dpi with 100 red fluorescent Mm. Scale bar, 60 μm.

(B) Tracks of macrophage movement following granuloma formation in *snapc1b* mutant larvae and WT siblings shown in (A). Tracks are coded for speed. Tracks created by vacuolated macrophages are indicated with an asterisk.

(C and D) Speed (C) and displacement (D) of *snapc1b* mutant and WT sibling macrophages in (A and B). Statistical significance was assessed using one-way ANOVA with Sidak's post-test.

hypothesized that mutants would have an accumulation of extracellular apoptotic bodies in the brain as seen in several human lysosomal storage disorders (Huang et al., 1997). They did (Figures 2E and 2F). Furthermore, a global reduction in apoptosis induced by the pancaspase inhibitor Q-VD-OPh reduced extracellular cell debris (Figure S3A) and decreased the number of macrophages with lysosomal accumulations of the debris and with enlarged lysosomes (Figures S3B and S3C).

Together, these findings show that *snapc1b* mutant macrophages become irreversibly vacuolated due to their inability to degrade phagocytosed cell debris. As more and more macrophages lose their migratory capability, a deficit of functioning macrophages may develop.

Macrophage Migration Deficit Underlies Granuloma Breakdown in *snapc1b* Mutants

We recently showed that reduction in the macrophage supply reduces granuloma macrophage replenishment to the point at which apoptotic infected macrophages, failing to be engulfed, undergo secondary necrosis (Pagán et al., 2015). Having observed that the vacuolated macrophages of *snapc1b* mutants failed to migrate to dying cells in the uninfected state and failed to migrate to newly infecting bacteria, we wondered whether they were also unable to migrate to dying cells in the tuberculous granuloma. If so, a functional macrophage deficiency could develop in the context of global macrophage excess, providing an explanation for our observation of early granuloma breakdown.

We performed detailed time-lapse confocal imaging of forming granulomas in wild-type and *snapc1b* mutant larvae over ~18 hr. Wild-type granulomas retained cellularity over this period through continuous influx of macrophages (Movie S2; Figures 3A and 3B). In contrast, Snapc1b-deficient granulomas broke down soon after formation (Movie S2). Mutant granulomas were surrounded by mostly vacuolated macrophages that failed to migrate into the granuloma (Figures 3A and 3B; Movie S2). The migration deficit in *snapc1b* mutants was specific to the vacuolated macrophages; the morphologically normal macrophages in the mutants maintained displacements and speeds similar to those of wild-type animals (Figures 3C and 3D; Movie S2). Thus, macrophage lysosomal storage leads to granuloma breakdown by preventing migration to the forming structure and is functionally equivalent to a global macrophage deficiency. Our findings suggest that, once the *snapc1b* mutants have exhausted their migration-competent macrophages, the tuberculous granuloma breaks down resulting in bacterial cording.

Lysosomal Cathepsin Deficiency Underlies *snapc1b* Mutant Macrophage Abnormalities and Hypersusceptibility to *M. marinum*

Human SNAPC1 is involved in global RNA polymerase II-dependent transcription (Baillat et al., 2012). RNA-seq analysis of *snapc1b* mutants and wild-type siblings revealed more than 1,000 differentially expressed genes in the mutant (Table S3), suggesting zebrafish Snapc1b functions in a similar manner. How might a broadly acting transcriptional regulator produce such specific phenotypes? Guided by the *snapc1b* mutant phenotype, we analyzed the RNA-seq dataset for lysosomal genes including those associated with human lysosomal storage disorders (Table S2) (Platt et al., 2012). Only two, the myeloid cell-specific lysosomal cysteine cathepsins B and L1 (*ctsbb* and *ctsl1*) (Heng et al., 2008), were underrepresented in the mutant, at 9% and 13% of wild-type levels, respectively (Table S2), and we confirmed their commensurate reduction by qRT-PCR analysis (85% and 83%, respectively) (Figure 4A; data not shown). We were able to test lysosomal cathepsin activity in situ using MagicRed (MR)-Cathepsin L, a modified cathepsin L target sequence, which fluoresces only when cleaved (Peri and Nüsslein-Volhard, 2008). In wild-type animals, brain macrophages quickly cleaved injected MR-cathepsin L; this number was reduced as expected by administration of the irreversible

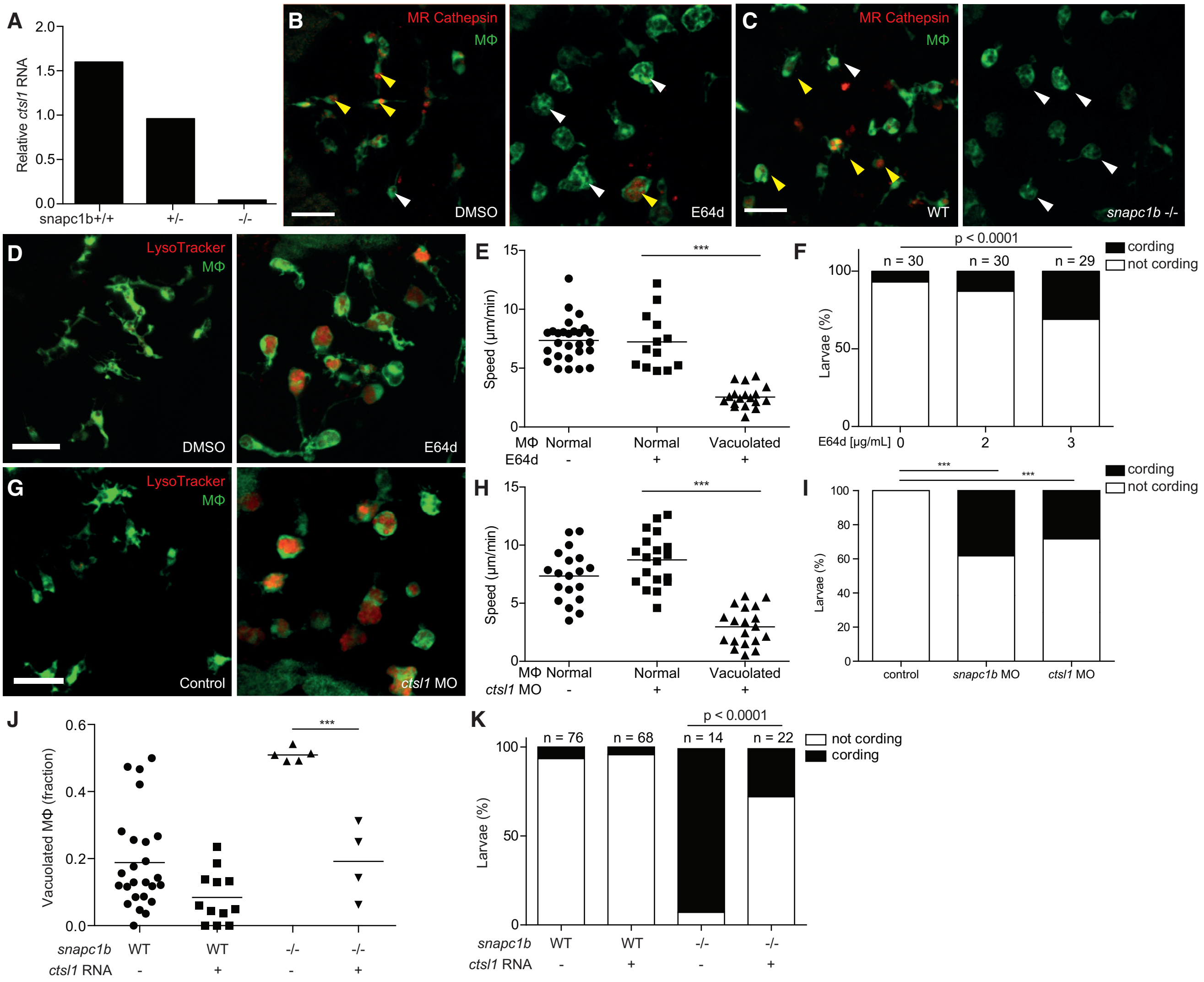

Figure 4. Cathepsin L Deficiency Causes *snapc1b* Mutant Vacuolated Macrophage Morphology and Susceptibility to *M. marinum*

(A) Quantitative real-time PCR of relative *ctsl1* transcript in *snapc1b*$^{+/-}$ incross larvae at 6 dpf. Values normalized to transcript level of the heterozygous larvae, representative of two experiments.

(B and C) Confocal images of green fluorescent macrophages in larvae injected with red fluorescent MR-Cathepsin L at 3 dpf, either following treatment with E64d or DMSO control at 2dpf (B) or in *snapc1b* mutants and WT siblings (C). Yellow or white arrowheads denote macrophages that are positive or negative for MR-Cathepsin, respectively. Scale bar, 50 μm.

(D) Confocal images of green fluorescent macrophages stained with LysoTracker red in the brains of 3-dpf E64d-treated and DMSO control larvae. Scale bar, 50 μm.

(E) Average macrophage speeds during a 5-hr movie in the brains of 3-dpf E64d-treated and DMSO control larvae.

(F) Quantification of bacterial cording in DMSO control and E64d-treated larvae at 5 dpi with 150 Mm.

(G) Confocal images of green fluorescent macrophages stained with LysoTracker red in the brains of 3-dpf *ctsl1* morphants and control larvae. Scale bar, 50 μm.

(H) Average macrophage speeds during a 5-hr movie in the brains of 3-dpf *ctsl1* morphants and control larvae.

(I) Quantification of bacterial cording in control, *snapc1b*, and *ctsl1* morphants at 5 dpi with 200 Mm.

(J) Quantification of vacuolated macrophages in the brains of 3-dpf WT or *snapc1b* mutant larvae following injection of *ctsl1* RNA or control at 0 dpf.

(K) Quantification of bacterial cording at 2 dpi with 215 Mm in the HBV of *snapc1b* mutants and WT siblings following injection of *ctsl1* RNA or control.

Statistical significance was assessed by ANOVA with Sidak's post test (E, H, and J) or Fisher's exact test (F, I, and K). See also Figure S4.

pan-cysteine cathepsin inhibitor, E64d (42.7% of macrophages in control versus 3.63% in E64d-treated larvae, $p < 0.0001$) (Murray et al., 1997) (Figure 4B). *snapc1b* mutants displayed reduced MR-cathepsin-L cleavage compared to wild-type siblings, indicative of reduced lysosomal cathepsin L activity (47.2% of macrophages in WT animals versus 5.3% in mutants, $p < 0.0001$) (Figure 4C).

We next tested whether cysteine cathepsin deficiency underlies all of the *snapc1b* mutant phenotypes. Inhibition of cysteine cathepsins by E64d recapitulated both baseline and infected

snapc1b mutant phenotypes—macrophage lysosomal storage with accompanying migratory defects at baseline, and hypersusceptibility to infection with bacterial cording (Figures 4D–4F). We attempted morpholino knockdown of *ctsbb* and *ctsl1* to probe their individual culpabilities in the *snapc1b* mutant phenotypes (Table S1). As the *ctsbb* morpholino was highly toxic, we could only pursue *ctsl1* further. *ctsl1* morphants recapitulated the *snapc1b* phenotypes (Figures 4G–4I). Transient overexpression of *ctsl1* mRNA in *snapc1b* mutant larvae restored normal macrophage morphology in uninfected *snapc1b* mutants and rescued cording (Figures 4J and 4K). Together these experiments implicate cysteine cathepsins in *snapc1b* hypersusceptibility resulting from macrophage incapacitation. Our data ascribe a substantial portion of *snapc1b* phenotypes to cathepsin L1 deficiency though we cannot rule out a minor role for cathepsin B deficiency. Prior findings that cathepsin L knockout mice are not hypersusceptible to *M. tuberculosis* (Nepal et al., 2008) may reflect functional redundancies present in the mouse but not the zebrafish.

Our finding that cathepsin L1 deficiency mediated hypersusceptibility prompted us to ask whether this lysosomal hydrolase might play a role in macrophage microbicidal activity for two reasons. First, a deficit in macrophage microbicidal activity (e.g., through TNF deficiency) has been shown to result in granuloma breakdown with bacterial cording (Tobin et al., 2010). Second, in vitro, cathepsin L has been reported to indirectly facilitate mycobacterial killing by cleaving ubiquitin into microbicidal peptides (Alonso et al., 2007). However, we found that macrophages of both *snapc1b* mutants and *ctsl1* morphants restricted bacterial growth normally (Figure S4). These findings suggest that cathepsin L-mediated macrophage microbicidal capacity is dispensable in vivo and confirms that its deficiency induces susceptibility by compromising macrophage migration.

Zebrafish Models of Human Lysosomal Storage Disorders Display Accelerated Tuberculous Granuloma Breakdown

While cathepsin deficiency causes protein accumulation in lysosomes, many human genetic lysosomal storage disorders result from the accumulation of diverse lipid species (Platt et al., 2012). Patients with Gaucher's disease, the most common lysosomal storage disease, have macrophages with migration defects in vitro (Aflaki et al., 2014; Liel et al., 1994) and are susceptible to a variety of pathogens including mycobacteria, though this may be due to concomitant immune defects including pancytopenias (Aker et al., 1993; Jain and Yelwatkar, 2011; Machaczka et al., 2014; Zimran, 2011). We asked whether the mechanism of susceptibility uncovered for *ctsl1* deficiency extended to lysosomal storage disorders characterized by lipid accumulation. Knockdown of the zebrafish orthologs of the genes responsible for Gaucher's disease, Tay-Sachs disease, and metachromatic leukodystrophy produced increased numbers of vacuolated macrophages with enlarged lysosomes and migratory defects (Tables S1 and S2; Figures 5A–5C). Upon infection, all three exhibited early granuloma breakdown and bacterial cording (Figures 5D–5F). Thus, etiologically diverse lysosomal storage disorders can increase susceptibility to tuberculous infection, regardless of the nature of the accumulated material.

Macrophage Lysosomal Storage Disrupts Endocytic Recycling

Our work so far had linked macrophage lysosomal storage to impaired migration to increased susceptibility to mycobacteria. Having understood the cellular basis of the link between impaired macrophage migration and susceptibility, we sought to understand how macrophage lysosomal storage might impair migration. Recycling between the endosomal and plasma membranes is known to be required for cell migration. This recycling delivers membrane lipids and proteins required for movement to the plasma membrane and facilitates adjustments in cell-surface area that are critical for cell motility (Bretscher and Aguado-Velasco, 1998; Traynor and Kay, 2007; Veale et al., 2010). Lysosomes share contents with endosomes, and recent evidence suggests that, like endosomes, they participate in recycling to the plasma membrane (Bright et al., 2005, 2015). Accordingly, embryonic fibroblasts isolated from two mouse models of severe lysosomal storage disorders display broad dysregulation of the entire endocytic pathway (Fraldi et al., 2010).

We asked whether endocytic recycling was disrupted in the zebrafish macrophages with lysosomal storage by monitoring the fate of fluorescently labeled high-molecular-weight dextran (10,000 MW) in normal animals and those with macrophage lysosomal storage. Following endocytosis, high-molecular-weight dextran is trafficked to lysosomes but not readily degraded, and its loss from lysosomes strictly reflects trafficking from them. In pulse-chase experiments, dextran-labeled lysosomes have been shown to fuse rapidly with endosomes, and several hours later the dextran is released into the extracellular medium suggesting subsequent fusion events that involve trafficking to the plasma membrane (Bright et al., 2015).

We injected fluorescent dextran into the brains of 3-dpf zebrafish larvae—wild-type, cathepsin deficient by E64d treatment, and *gba*-deficient morphants. In all groups, 74%–82% of the macrophages had taken up the dye within 5 hr (Figure 6). After 30 hr, only 33% and 39% of the macrophages in the wild-type fish retained the dextran, whereas 77% and 79% did in the cathepsin and *gba*-deficient animals, respectively (Figure 6), suggesting that stalling of the entire endocytic system is a common feature of lysosomal storage diseases and underlies the defective migration displayed by vacuolated macrophages.

Our finding that the lysosomal accumulation of diverse biomolecules compromises endocytic recycling, and thus cell motility, suggested a common mechanism independent of the specific lysosomal substrate. If so, then lysosomal storage induced by non-biological particles should produce the same phenotypes. We injected beads into the HBV, which were phagocytosed by brain resident macrophages (Figure S5A). Bead-laden macrophages were compromised for homeostatic migration and exhibited disruption of endocytic recycling (Figures S5B–S5E).

Lysosomal Accumulation Compromises Macrophage Migration to Newly Infecting Mycobacteria

In addition to their role in forming and maintaining the granuloma, resident macrophages are the first cells to migrate to mycobacteria at the initial site of infection (Cambier et al., 2014a; Philips and Ernst, 2012). This first macrophage-mycobacterium interaction can be visualized in the zebrafish hindbrain ventricle (HBV),

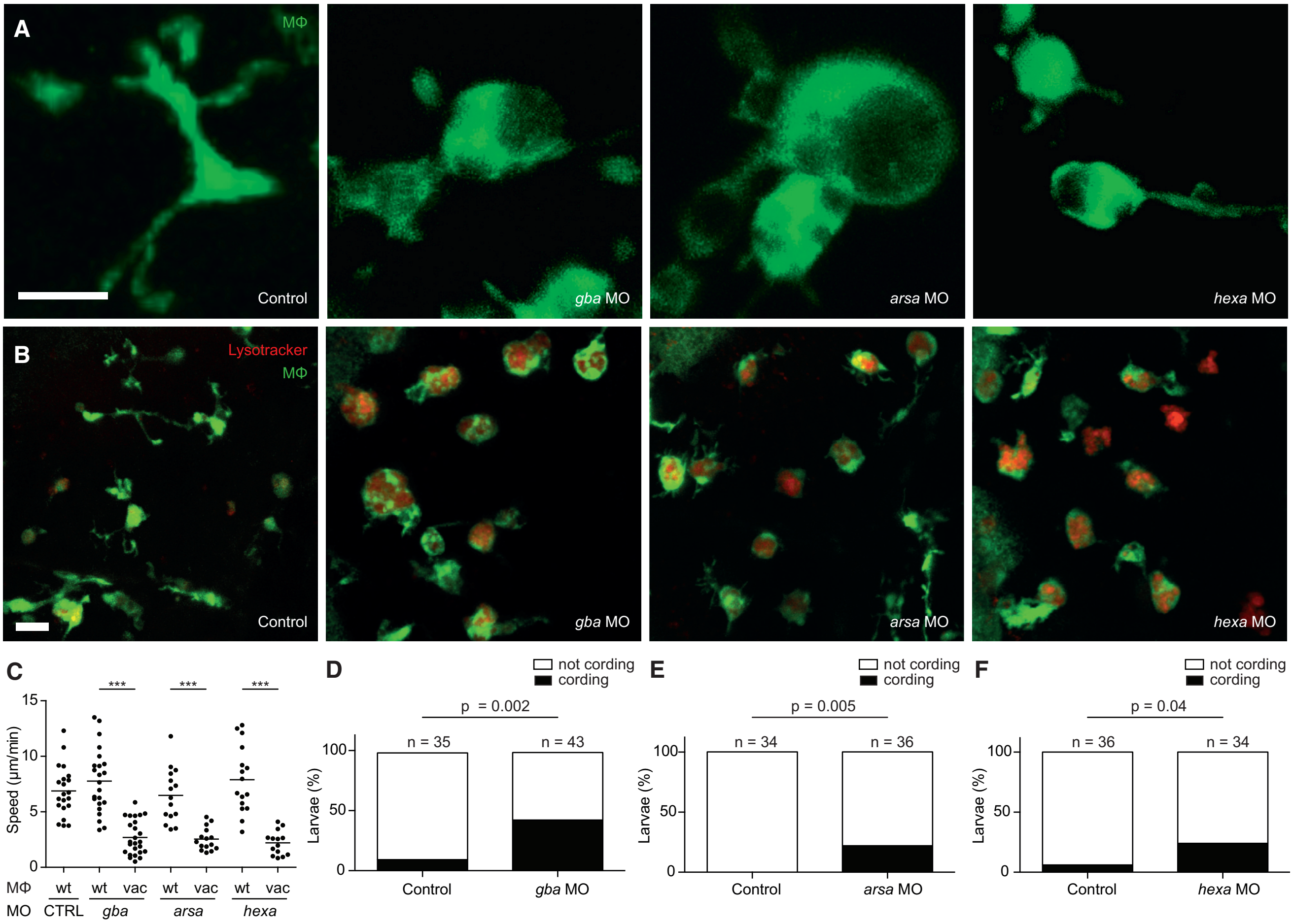

Figure 5. Lysosomal Storage Disorders Disrupt Macrophage Migration and Cause Granuloma Breakdown

(A and B) Confocal images of green fluorescent macrophages in the brain of 3-dpf control and morphant larvae, unstained (A) or following staining with LysoTracker Red (B). Scale bars, 10 μm.

(C) Quantification of average macrophage speed in control and morphant larvae by macrophage morphology (wt, wild-type; vac, vacuolated).

(D–F) Quantification of bacterial cording in control and morphant larvae at 3 dpi with 200 Mm. Statistical significance was determined using paired t tests with Bonferroni correction (C) and Fisher's exact test (D–F).

a cavity into which phagocytes migrate in response to mycobacteria (Cambier et al., 2014b). In the *snapc1b* mutant, only the subset of brain-resident macrophages that still had normal morphology migrated to the bacteria and phagocytosed them, while the vacuolated macrophages, failing to migrate from the adjacent brain parenchyma, remained uninfected (data not shown). We could not directly test the migration of bead-laden brain resident macrophages, as only a minority of them engulfed sufficient numbers of beads injected into the HBV. So we injected either beads or the nuclear stain Hoechst 33342 into the caudal vein followed by bacteria into the HBV (Figure S5F). As observed previously, the Hoechst-stained macrophages could be discerned by their blue nuclei and were morphologically normal (Figure S5G) (Davis and Ramakrishnan, 2009). After confirming that similar numbers of circulating macrophages were labeled blue by either dye or beads, we injected bacteria into the HBV (Figure S5F). Multiple Hoechst-positive macrophages migrated to the HBV in response to the bacteria, as expected (Davis and Ramakrishnan, 2009), but hardly any bead-filled ones did (Figures S5G–S5I). Thus, the accumulation of indigestible inert particles in macrophage lysosomes compromises their migration so as to preclude their ability to phagocytose infecting mycobacteria.

Lysosomal Accumulation of Tobacco Smoke Particulates Compromises Macrophage Migration to *M. tuberculosis* in Humans

Human TB is thought to begin when mycobacteria are phagocytosed by pulmonary alveolar macrophages, the resident macrophages at the air-lung interface (Bates et al., 1965; Hocking and Golde, 1979; Ratcliffe and Wells, 1948; Verrall et al., 2014). Consistent with their role in primary defense against diverse inhaled bacteria (Green and Kass, 1964; Hocking and Golde, 1979), the ability of a substantial number of individuals to clear *M. tuberculosis* early after infection has been ascribed to the microbicidal activity of the alveolar macrophage (Verrall et al.,

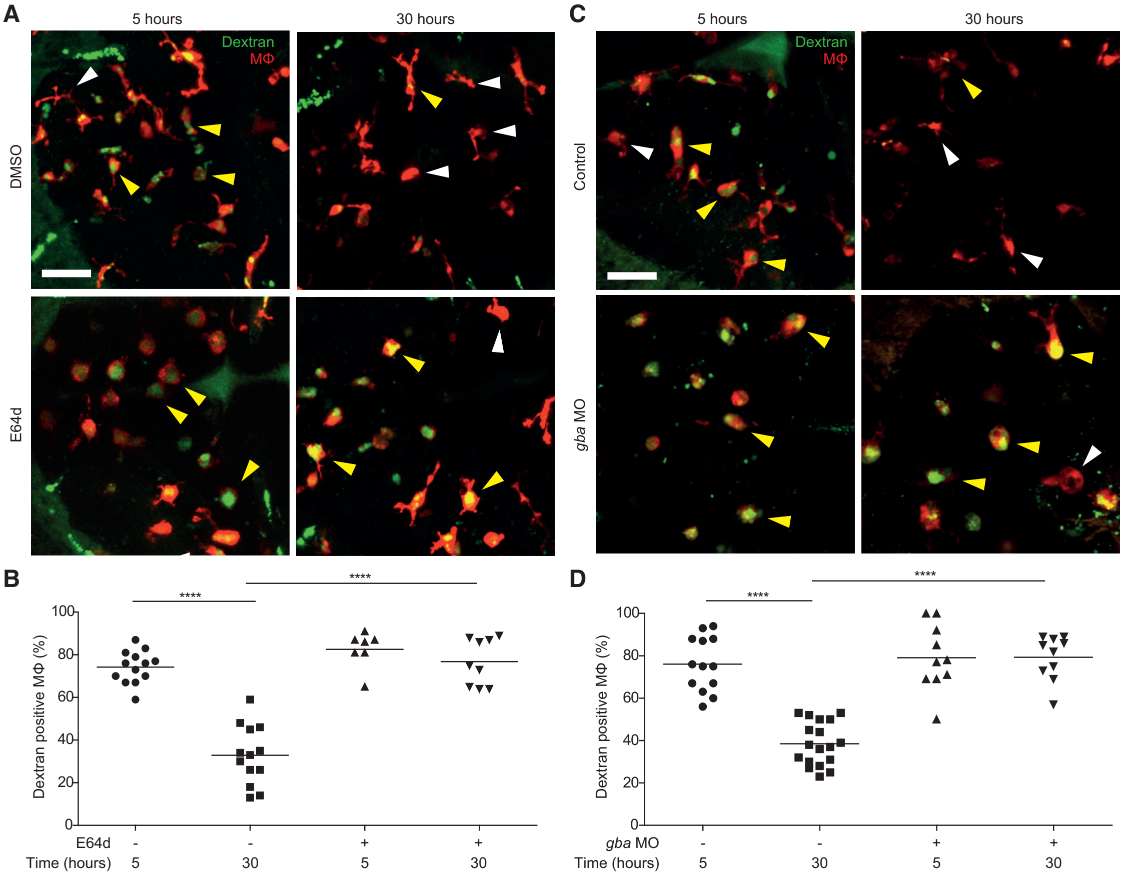

Figure 6. Macrophage Lysosomal Storage Disrupts Endocytic Recycling

(A) Confocal images of red fluorescent macrophages following injection of green fluorescent dextran in E64d-treated and DMSO control larvae (3 dpf) at 5 and 30 hr post-injection. Yellow and white arrowheads denote macrophages with and without dextran, respectively. Scale bar, 50 μm.

(B) Quantification of the percentage of macrophages that are positive for dextran in E64d-treated and DMSO control larvae (3 dpf) at 5 and 30 hr post-injection.

(C) Confocal images of red fluorescent macrophages following injection of green fluorescent dextran in *gba* morphants and control larvae (3 dpf) at 5 and 30 hr post-injection. Yellow and white arrowheads denote macrophages with and without dextran, respectively. Scale bar, 50 μm.

(D) Quantification of the percentage of macrophages that are positive for dextran in *gba* morphants and control larvae (3 dpf) at 5 and 30 hr post-injection.

See also Figure S5.

2014). Despite their central defensive role, many alveoli are normally devoid of macrophages because their numbers are limiting (Betz et al., 1993; Ferin, 1982). Therefore, efficient and complete phagocytosis of inhaled particulates is predicated on the rapid migration of alveolar macrophages from nearby alveoli (Lehnert, 1992; Peão et al., 1993). This migration should be particularly relevant to TB, the outcome of which depends upon the fate of the 1-3 bacteria deposited in a distal alveolus, which might not contain a macrophage (Bates et al., 1965; Ratcliffe and Wells, 1948). In light of our findings that bead-laden macrophages were compromised for migration to newly infecting bacteria in the zebrafish, we wondered whether the accumulation of tobacco smoke particulates in the alveolar macrophages of cigarette smokers (Harris et al., 1970; Martin, 1973) might be similarly compromised, accounting for the poorly understood association between smoking and the acquisition of new TB infection (Anderson et al., 1997; den Boon et al., 2005). If an infecting mycobacterium were to be deposited in a macrophage-deficient alveolus and not rapidly phagocytosed by nearby alveolar macrophages rendered immotile secondary to lysosomal engorgement, it would have an extended period of extracellular growth before engulfment by alveolar macrophages or other myeloid cells recruited from afar.

We examined alveolar macrophages obtained from smokers, nonsmokers, and ex-smokers by bronchoalveolar lavage (O'Leary et al., 2014) (Table S4; Supplemental Experimental Procedures). The majority of smokers' alveolar macrophages exhibited vacuolated morphology and had accumulated opaque material in large lysosomal inclusions as evidenced by staining with neutral red, a vital dye that concentrates in lysosomes (Figures 7A and 7B). The abnormal cells were readily identified by their autofluorescence, consistent with previous findings (Martin, 1973). These cells were present at a lower frequency in ex-smokers and virtually absent in nonsmokers (Figures 7A and 7B).

Using a transwell assay, we confirmed prior reports that alveolar macrophages from nonsmokers and ex-smokers migrate

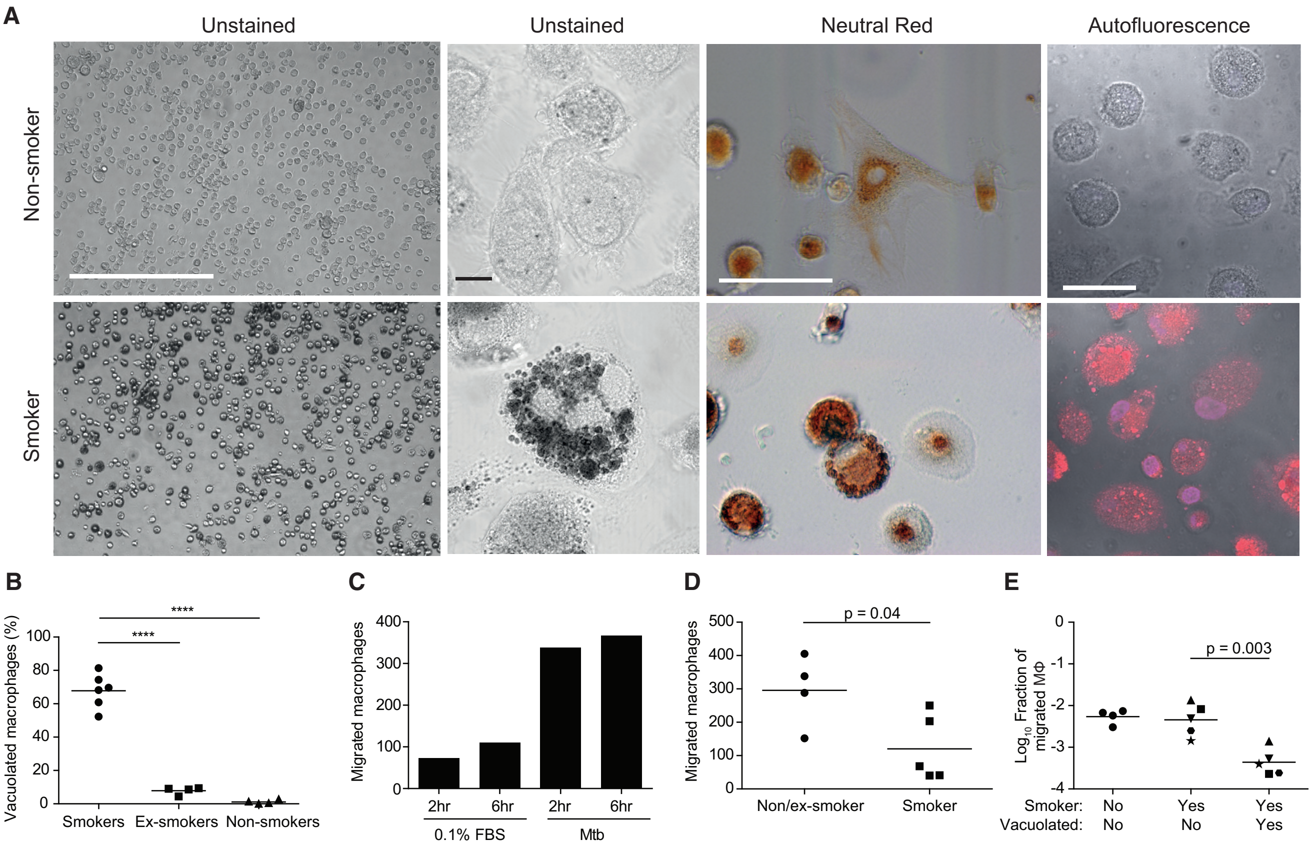

Figure 7. Lysosomal Accumulation of Tobacco Smoke Products in Alveolar Macrophages Compromises Migration to *M. tuberculosis*

(A) Representative images showing the characteristics of macrophages isolated by bronchoalveolar lavage from smokers and nonsmokers. Scale bars (left to right), 400, 10, 20, and 10 μm.

(B) Percentage of vacuolated macrophages was assessed in smokers, ex-smokers, and nonsmokers. Vacuolated macrophages were scored based on their autofluorescence and morphology.

(C) Number of macrophages that migrated through a transwell was assessed at 2 and 6 hr of incubation with either 0.1% fetal bovine serum (FBS) or Mtb H37Ra using macrophages from an ex-smoker. Values represent averages of a single experiment performed in triplicate.

(D) Number of macrophages from non/ex-smokers that migrated through a transwell toward Mtb H37Ra (assessed following 2 hr incubation).

(E) Fraction of macrophages that migrated in the transwell assay calculated from initial versus migrated macrophages of each morphology. Samples from smokers are split into vacuolated and normal with unique symbols for each patient.

Statistical significance was assessed by one-way ANOVA with Sidak's post-test (B), Student's t test (D), paired t test (E). See also Figure S6 and Table S4.

to zymosan-activated serum, a rich source of the chemoattractant C5a (Figure S6A) (Barlow et al., 2008; Sweeney et al., 2015). In this assay, nonsmokers' and ex-smokers' alveolar macrophages also migrated to *M. tuberculosis* within 2 hr (Figures 7C and S6B). Migration of smokers' macrophages to *M. tuberculosis* was impaired (Figure 7D). Our hypothesis predicts that this overall migration impairment is due to a selective inability of the vacuolated subset to migrate. Indeed, by calculating the fraction of the smokers' normal versus vacuolated macrophages that migrated, we found that the migration impairment was specific to the vacuolated subset (Figure 7E). In sum, we show that the majority of smokers' macrophages fail to migrate toward *M. tuberculosis* due to lysosomal accumulation of particulates, and their nonparticipation may contribute to the susceptibility of these individuals to TB.

DISCUSSION

We have described a zebrafish mutant in the *snapc1b* basal transcription factor component that displays the hallmark characteristics of human lysosomal storage disorders and is hypersusceptible to *M. marinum* infection. RNA-seq of *snapc1b* mutants revealed reduced expression of the lysosomal degradative cathepsins L and B, and pharmacological inhibition of cathepsin activity or knockdown of cathepsin L recapitulates the key mutant phenotypes of vacuolated macrophage morphology and susceptibility to infection.

Though cathepsin L is involved in the lysosomal degradation of phagocytosed material, its deficiency here mediates susceptibility to mycobacteria not by reducing macrophage microbicidal capacity but rather by causing lysosomal accumulation of undigested cell debris. This disrupts endocytic membrane recycling

and thereby compromises macrophage migration in a variety of contexts. By modeling human lysosomal storage diseases in the zebrafish, we find that the accumulation of diverse substrates causes susceptibility to infection through this same mechanism. Our studies provide insights into the fundamental and common role played by macrophages as scavengers of dying cells during homeostasis and during tuberculous granuloma maintenance. These insights shed light on the protective role of tissue macrophages in early tuberculous infection and how lysosomal accumulation of tobacco smoke products may compromise this role.

Macrophage Migration Defects Due to Lysosomal Accumulation of Undigested Cell Debris Contribute to the Pathogenesis of Lysosomal Storage Disorders

Sequential live visualization of the developing *snapc1b* mutant highlights the continuous scavenging role of macrophages under homeostatic conditions. Our work suggests that the accumulation of undigested cell debris in macrophage lysosomes may itself contribute substantially to the pathogenesis of human lysosomal storage diseases. We find that defects in macrophage degradative function render the cell vacuolated, immotile, and unable to further perform a critical scavenging function, which depends on directed migration to the dying cell (Hochreiter-Hufford and Ravichandran, 2013). This may contribute to the accumulation of unphagocytosed debris from cells undergoing apoptosis in the course of homeostatic tissue remodeling and repair, and the pathological consequences of their secondary necrosis.

The increased number of apoptotic bodies observed in human lysosomal storage disorders has been attributed to increased cell death triggered by the accumulation of lysosomal substrates (Huang et al., 1997). However, macrophages also accumulate lysosomal substrates in a variety of human lysosomal storage diseases (Kieseier et al., 1997); our findings suggest that the resultant immotility of an increasing proportion of macrophages may contribute to the accumulation of dead cells. Because tissue turnover is high in the developing brain, the macrophage scavenging deficit we propose may be particularly relevant for the pathogenesis of the neurological manifestations of lysosomal storage disorders hitherto attributed to neuronal dysfunction (Jeyakumar et al., 2005). Hematopoietic stem cell transplants in humans and mice improve clinical manifestations of lysosomal storage disorders, including neurological ones that are recalcitrant to enzyme replacement therapy (Biffi et al., 2004; Malatack et al., 2003; Norflus et al., 1998). In light of our findings, we speculate that hematopoietic stem cell transplantation alleviates disease pathology by restoring macrophage degradative function and consequently migration to engulf cell debris.

Macrophage Migration Defects Caused by Lysosomal Accumulation Promote Tuberculous Granuloma Breakdown

Our studies of the *snapc1b* mutant, in which vacuolated macrophages fail to migrate into the tuberculous granuloma, reveal the inextricable link between macrophage homeostatic and immune function. Like the brain, the forming tuberculous granuloma is an environment with high cell turnover and the maintenance of its cellularity depends on the continuous migration of new macrophages that engulf dying infected macrophages (Davis and Ramakrishnan, 2009; Pagán et al., 2015). In the context of tissue remodeling, the clearance of dying cells prevents their secondary necrosis and release of inflammatory material into the extracellular space (Hochreiter-Hufford and Ravichandran, 2013). Likewise, in the TB granuloma, timely engulfment of dying infected macrophages prevents their secondary necrosis and release of bacteria into the extracellular milieu (Pagán et al., 2015). Granuloma breakdown is clinically significant because it increases both disease severity and risk of transmission (Cambier et al., 2014a).

Human lysosomal storage disorders are rare and often lethal within the first year of life, and thus unlikely to be significant contributors to the global burden of TB. Likewise, *snapc1b* mutant zebrafish fail to reach adulthood, and *SNAPC1*-null mutations in humans are likely embryonic lethal. However, even relatively small reductions in the macrophage supply to the granuloma can accelerate its breakdown (Pagán et al., 2015). It is possible that subtle alterations in macrophage degradative function, caused by altered expression of SNAPC1, or lysosomal cathepsins or other hydrolases, could create local macrophage deficits and increased susceptibility to TB. Thus, macrophage lysosomal accumulation from diverse genetic etiologies may together be not insignificant contributors to the global TB burden.

Lysosomal Accumulation in Alveolar Macrophages of Smokers May Contribute to TB Susceptibility

Finally, our findings that lysosomal storage also compromises the migration of lung resident alveolar macrophages to mycobacteria suggests a mechanism for the observed susceptibility of smokers to new TB infection. There is accumulating evidence for a role for alveolar macrophages being first-responding protective cells in TB. In mice, aerosolized *M. tuberculosis* is found almost exclusively in alveolar macrophages for the first 7 days, after which infection moves into other myeloid cells such as monocytes and dendritic cells recruited from the lung interstitium or circulation (Srivastava et al., 2014; Urdahl, 2014). Their greater microbicidal capacity is mirrored in humans whose alveolar macrophages inhibit *M. tuberculosis* growth in contrast to peripheral blood monocytes, which are growth permissive (Aston et al., 1998). Our findings suggest that migration defects resulting from macrophage lysosomal engorgement impede the rapid engulfment (and therefore eradication) of infecting microbes at points of entry and may therefore facilitate bacterial entry into growth-permissive cells.

Cigarette smoking increases not only the risk of progression to active pulmonary TB disease, but also the risk of new TB infection, suggesting defective early response mechanisms in smokers (Anderson et al., 1997; den Boon et al., 2005; Gyawali et al., 2012). Smokers' alveolar macrophages phagocytose bacteria and yeast normally and have normal bactericidal activity against *M. tuberculosis* (Cohen and Cline, 1971; Harris et al., 1970; O'Leary et al., 2014). The incapacitation of alveolar macrophages by tobacco smoke particulates may contribute to increased risk of infection in two ways: (1) delayed time to phagocytosis by the alveolar macrophage, allowing for a longer

extracellular growth period by the bacteria, and (2) increased chance of initial phagocytosis by a recruited, growth-permissive macrophage.

In addition to providing an explanation for the increased susceptibility of individuals with genetic lysosomal storage disorders to respiratory, skin, and mucosal infections (Jain and Yelwatkar, 2011; Machaczka et al., 2014), our findings may constitute a basis for the susceptibility of smokers to other respiratory infections (Bagaitkar et al., 2008; Lin et al., 2007), Finally, this mechanism may also contribute to the poorly understood association between indoor air pollution and TB (Sumpter and Chandramohan, 2013).

Smokers' increased susceptibility to infection may be reversible. A longitudinal study of alveolar macrophages after transplant of a smoker's lung into a nonsmoker revealed a progressive decrease in "smokers alveolar macrophages" from >90% to 3% in 3 years (Marques et al., 1997). In our cohort, not only did ex-smokers have significantly fewer alveolar macrophages with lysosomal storage than smokers, but overall migration to *M. tuberculosis* was restored. These findings provide an additional rationale for smoking cessation as a prescription for TB prevention.

EXPERIMENTAL PROCEDURES

Detailed methods and bacterial and zebrafish strains associated with all procedures below are available in Supplemental Experimental Procedures.

Zebrafish Husbandry and Larval Injections

Zebrafish husbandry and experiments were conducted according to guidelines from the UK Home Office, and the US NIH (approved by the University of Washington Institutional Animal Care and Use Committee). The wild-type AB strain was used for experiments except those in which the *snapc1b(fh111)* line or transgenic lines were used. Unless noted, crosses using *snapc1b(fh111)* were performed as heterozygote incrosses, which were genotyped at the completion of the experiment, to ensure blinded scoring of phenotypes. Except where noted, "WT" refers to *snapc1b*$^{fh111/+}$ and *snapc1b*$^{+/+}$. Bacteria, beads, and dye were injected into the caudal vein and/or hindbrain ventricle.

Human Alveolar Macrophage Experiments

Alveolar macrophages (AM) were retrieved at bronchoscopy after informed consent and as approved by the Research Ethics Committee of St. James' Hospital, using a protocol that preserves viability of macrophages from both smokers and nonsmokers (O'Leary et al., 2014). Macrophage migration and microscopical visualization procedures are detailed in Supplemental Experimental Procedures.

Bacterial Strains

Wild-type *M. marinum* (Mm) (strain M - ATCC #BAA-535) expressing tdTomato under the constitutive promoter *msp12* was used for fluorescence microscopy and quantification of intracellular bacterial burdens (Takaki et al., 2013). The attenuated Δ*erp* mutant Mm was used to enumerate intracellular bacteria (Cosma et al., 2006; Takaki et al., 2013), and WT Mm was used for all other assays. Bacterial were cultures and prepared for injection as described (Takaki et al., 2013).

M. tuberculosis H37Ra (ATCC 25177) was used for the human alveolar macrophage studies and prepared as described in Supplemental Experimental Procedures.

Statistical Analyses

Statistical analyses were performed using Prism 6 (GraphPad). Not significant, $p \geq 0.05$, *$p < 0.05$; **$p < 0.01$; ***$p < 0.001$; ****$p < 0.0001$.

ACCESSION NUMBERS

The accession number for the RNA-seq data reported in this paper is GEO: GSE74196.

SUPPLEMENTAL INFORMATION

Supplemental Information includes Supplemental Experimental Procedures, six figures, four tables, and two movies and can be found with this article online at http://dx.doi.org/10.1016/j.cell.2016.02.034.

AUTHOR CONTRIBUTIONS

R.D.B., S.L., C.J.C., J.C., K.T., C.B.M., D.M.T., and L.R. conceived, designed, and analyzed zebrafish experiments; R.D.B., S.L., C.J.C., J.C., K.T., and D.M.T. performed these experiments; S.L., M.P.O., S.M.O., J.K., and L.R. conceived, designed, and analyzed human experiments; M.P.O. and S.M.O. performed these experiments; S.L., R.D.B., and L.R. wrote the paper with input from J.K., D.M.T., M.P.O., S.M.O., and C.B.M.; K.T., and S.L. prepared the figures.

ACKNOWLEDGMENTS

We thank P. Luzio, G. Griffiths, and R. Kay for insights and advice on lysosome biology and cell migration; G. Godaly for advice on human macrophage cell migration assays; P. Edelstein, A. Pagán, and S. Falkow for manuscript review; N. Goodwin and R. Keeble for zebrafish husbandry; and F. O'Connell, the research nurses of the Wellcome Trust-HRB Clinical Research Facility, and the staff and patients of the St. James's Hospital Bronchoscopy clinic, Dublin. This work was supported by the Wellcome Trust (L.R.), the NIH (R37AI054503, L.R., R01NS082567, C.B.M., 5F30HL110455, R.D.B., 1DP2-OD008614, D.M.T.), the National Institute of Health Research Cambridge Biomedical Research Centre (L.R.), the Health Research Board of Ireland (HRA_POR/2013/387, M.P.O'S. and CSA/2012/16, J.K.), and The Royal City of Dublin Hospital Trust (grant 146, J.K.).

Received: July 24, 2015
Revised: November 30, 2015
Accepted: February 2, 2016
Published: March 24, 2016

REFERENCES

Abrams, J.M., White, K., Fessler, L.I., and Steller, H. (1993). Programmed cell death during Drosophila embryogenesis. Development *117*, 29–43.

Aflaki, E., Stubblefield, B.K., Maniwang, E., Lopez, G., Moaven, N., Goldin, E., Marugan, J., Patnaik, S., Dutra, A., Southall, N., et al. (2014). Macrophage models of Gaucher disease for evaluating disease pathogenesis and candidate drugs. Sci. Transl. Med. *6*, 240ra73.

Aker, M., Zimran, A., Abrahamov, A., Horowitz, M., and Matzner, Y. (1993). Abnormal neutrophil chemotaxis in Gaucher disease. Br. J. Haematol. *83*, 187–191.

Allen, T.C. (2008). Pulmonary Langerhans cell histiocytosis and other pulmonary histiocytic diseases: a review. Arch. Pathol. Lab. Med. *132*, 1171–1181.

Alonso, S., Pethe, K., Russell, D.G., and Purdy, G.E. (2007). Lysosomal killing of Mycobacterium mediated by ubiquitin-derived peptides is enhanced by autophagy. Proc. Natl. Acad. Sci. USA *104*, 6031–6036.

Anderson, R.H., Sy, F.S., Thompson, S., and Addy, C. (1997). Cigarette smoking and tuberculin skin test conversion among incarcerated adults. Am. J. Prev. Med. *13*, 175–181.

Aston, C., Rom, W.N., Talbot, A.T., and Reibman, J. (1998). Early inhibition of mycobacterial growth by human alveolar macrophages is not due to nitric oxide. Am. J. Respir. Crit. Care Med. *157*, 1943–1950.

Bagaitkar, J., Demuth, D.R., and Scott, D.A. (2008). Tobacco use increases susceptibility to bacterial infection. Tob. Induc. Dis. *4*, 12.

Baillat, D., Gardini, A., Cesaroni, M., and Shiekhattar, R. (2012). Requirement for SNAPC1 in transcriptional responsiveness to diverse extracellular signals. Mol. Cell. Biol. *32*, 4642–4650.

Barlow, P.G., Brown, D.M., Donaldson, K., MacCallum, J., and Stone, V. (2008). Reduced alveolar macrophage migration induced by acute ambient particle (PM10) exposure. Cell Biol. Toxicol. *24*, 243–252.

Bates, J.H., Potts, W.E., and Lewis, M. (1965). Epidemiology of primary tuberculosis in an industrial school. N. Engl. J. Med. *272*, 714–717.

Betz, P., Nerlich, A., Penning, R., and Eisenmenger, W. (1993). Alveolar macrophages and the diagnosis of drowning. Forensic Sci. Int. *62*, 217–224.

Biffi, A., De Palma, M., Quattrini, A., Del Carro, U., Amadio, S., Visigalli, I., Sessa, M., Fasano, S., Brambilla, R., Marchesini, S., et al. (2004). Correction of metachromatic leukodystrophy in the mouse model by transplantation of genetically modified hematopoietic stem cells. J. Clin. Invest. *113*, 1118–1129.

Bretscher, M.S., and Aguado-Velasco, C. (1998). EGF induces recycling membrane to form ruffles. Curr. Biol. *8*, 721–724.

Bright, N.A., Gratian, M.J., and Luzio, J.P. (2005). Endocytic delivery to lysosomes mediated by concurrent fusion and kissing events in living cells. Curr. Biol. *15*, 360–365.

Bright, N.A., Wartosch, L., and Luzio, J.P. (2015). Lysosome fusion in cultured mammalian cells. Methods Cell Biol. *126*, 101–118.

Cambier, C.J., Falkow, S., and Ramakrishnan, L. (2014a). Host evasion and exploitation schemes of Mycobacterium tuberculosis. Cell *159*, 1497–1509.

Cambier, C.J., Takaki, K.K., Larson, R.P., Hernandez, R.E., Tobin, D.M., Urdahl, K.B., Cosma, C.L., and Ramakrishnan, L. (2014b). Mycobacteria manipulate macrophage recruitment through coordinated use of membrane lipids. Nature *505*, 218–222.

Clay, H., Volkman, H.E., and Ramakrishnan, L. (2008). TNF signaling mediates resistance to mycobacteria by inhibiting bacterial growth and macrophage death. Immunity *29*, 283–294.

Clements, W.K., and Traver, D. (2013). Signalling pathways that control vertebrate haematopoietic stem cell specification. Nat. Rev. Immunol. *13*, 336–348.

Cohen, A.B., and Cline, M.J. (1971). The human alveolar macrophage: isolation, cultivation in vitro, and studies of morphologic and functional characteristics. J. Clin. Invest. *50*, 1390–1398.

Cosma, C.L., Swaim, L.E., Volkman, H., Ramakrishnan, L., and Davis, J.M. (2006). Zebrafish and frog models of Mycobacterium marinum infection. Curr. Protoc. Microbiol. *Chapter 10*, Unit 10B.12.

Davis, J.M., and Ramakrishnan, L. (2009). The role of the granuloma in expansion and dissemination of early tuberculous infection. Cell *136*, 37–49.

den Boon, S., van Lill, S.W., Borgdorff, M.W., Verver, S., Bateman, E.D., Lombard, C.J., Enarson, D.A., and Beyers, N. (2005). Association between smoking and tuberculosis infection: a population survey in a high tuberculosis incidence area. Thorax *60*, 555–557.

Ellett, F., Pase, L., Hayman, J.W., Andrianopoulos, A., and Lieschke, G.J. (2011). mpeg1 promoter transgenes direct macrophage-lineage expression in zebrafish. Blood *117*, e49–e56.

Ferin, J. (1982). Pulmonary alveolar pores and alveolar macrophage-mediated particle clearance. Anat. Rec. *203*, 265–272.

Flicek, P., Amode, M.R., Barrell, D., Beal, K., Billis, K., Brent, S., Carvalho-Silva, D., Clapham, P., Coates, G., Fitzgerald, S., et al. (2014). Ensembl 2014. Nucleic Acids Res. *42*, D749–D755.

Fraldi, A., Annunziata, F., Lombardi, A., Kaiser, H.J., Medina, D.L., Spampanato, C., Fedele, A.O., Polishchuk, R., Sorrentino, N.C., Simons, K., and Ballabio, A. (2010). Lysosomal fusion and SNARE function are impaired by cholesterol accumulation in lysosomal storage disorders. EMBO J. *29*, 3607–3620.

Green, G.M., and Kass, E.H. (1964). The role of the alveolar macrophage in the clearance of bacteria from the lung. J. Exp. Med. *119*, 167–176.

Gyawali, N., Gurung, R., Poudyal, N., Amatya, R., Niraula, S.R., Jha, P., and Bhattacharya, S.K. (2012). Prevalence of tuberculosis in household contacts of sputum smears positive cases and associated demographic risk factors. Nepal Med. Coll. J. *14*, 303–307.

Harris, J.O., Swenson, E.W., and Johnson, J.E., 3rd. (1970). Human alveolar macrophages: comparison of phagocytic ability, glucose utilization, and ultrastructure in smokers and nonsmokers. J. Clin. Invest. *49*, 2086–2096.

Heng, T.S., and Painter, M.W.; Immunological Genome Project Consortium (2008). The Immunological Genome Project: networks of gene expression in immune cells. Nat. Immunol. *9*, 1091–1094.

Henry, R.W., Mittal, V., Ma, B., Kobayashi, R., and Hernandez, N. (1998). SNAP19 mediates the assembly of a functional core promoter complex (SNAPc) shared by RNA polymerases II and III. Genes Dev. *12*, 2664–2672.

Hochreiter-Hufford, A., and Ravichandran, K.S. (2013). Clearing the dead: apoptotic cell sensing, recognition, engulfment, and digestion. Cold Spring Harb. Perspect. Biol. *5*, a008748.

Hocking, W.G., and Golde, D.W. (1979). The pulmonary-alveolar macrophage (second of two parts). N. Engl. J. Med. *301*, 639–645.

Hsu, C.L., Lin, W., Seshasayee, D., Chen, Y.H., Ding, X., Lin, Z., Suto, E., Huang, Z., Lee, W.P., Park, H., et al. (2012). Equilibrative nucleoside transporter 3 deficiency perturbs lysosome function and macrophage homeostasis. Science *335*, 89–92.

Huang, J.Q., Trasler, J.M., Igdoura, S., Michaud, J., Hanal, N., and Gravel, R.A. (1997). Apoptotic cell death in mouse models of GM2 gangliosidosis and observations on human Tay-Sachs and Sandhoff diseases. Hum. Mol. Genet. *6*, 1879–1885.

Jain, V.V., and Yelwatkar, S. (2011). Unusual presentation of adult Gaucher's disease: a long and difficult road to diagnosis. Indian J. Endocrinol. Metab. *15*, 224–226.

Jeyakumar, M., Dwek, R.A., Butters, T.D., and Platt, F.M. (2005). Storage solutions: treating lysosomal disorders of the brain. Nat. Rev. Neurosci. *6*, 713–725.

Kieseier, B.C., Wisniewski, K.E., and Goebel, H.H. (1997). The monocyte-macrophage system is affected in lysosomal storage diseases: an immunoelectron microscopic study. Acta Neuropathol. *94*, 359–362.

Lehnert, B.E. (1992). Pulmonary and thoracic macrophage subpopulations and clearance of particles from the lung. Environ. Health Perspect. *97*, 17–46.

Liel, Y., Rudich, A., Nagauker-Shriker, O., Yermiyahu, T., and Levy, R. (1994). Monocyte dysfunction in patients with Gaucher disease: evidence for interference of glucocerebroside with superoxide generation. Blood *83*, 2646–2653.

Lin, H.H., Ezzati, M., and Murray, M. (2007). Tobacco smoke, indoor air pollution and tuberculosis: a systematic review and meta-analysis. PLoS Med. *4*, e20.

Machaczka, M., Lorenz, F., Kleinotiene, G., Bulanda, A., Markuszewska-Kuczyńska, A., Raistenskis, J., and Klimkowska, M. (2014). Recurrent pulmonary aspergillosis and mycobacterial infection in an unsplenectomized patient with type 1 Gaucher disease. Ups. J. Med. Sci. *119*, 44–49.

Malatack, J.J., Consolini, D.M., and Bayever, E. (2003). The status of hematopoietic stem cell transplantation in lysosomal storage disease. Pediatr. Neurol. *29*, 391–403.

Marques, L.J., Teschler, H., Guzman, J., and Costabel, U. (1997). Smoker's lung transplanted to a nonsmoker. Long-term detection of smoker's macrophages. Am. J. Respir. Crit. Care Med. *156*, 1700–1702.

Martin, R.R. (1973). Altered morphology and increased acid hydrolase content of pulmonary macrophages from cigarette smokers. Am. Rev. Respir. Dis. *107*, 596–601.

Murray, E.J., Grisanti, M.S., Bentley, G.V., and Murray, S.S. (1997). E64d, a membrane-permeable cysteine protease inhibitor, attenuates the effects of parathyroid hormone on osteoblasts in vitro. Metabolism *46*, 1090–1094.

Nepal, R.M., Vesosky, B., Turner, J., and Bryant, P. (2008). DM, but not cathepsin L, is required to control an aerosol infection with Mycobacterium tuberculosis. J. Leukoc. Biol. *84*, 1011–1018.

Norflus, F., Tifft, C.J., McDonald, M.P., Goldstein, G., Crawley, J.N., Hoffmann, A., Sandhoff, K., Suzuki, K., and Proia, R.L. (1998). Bone marrow transplantation prolongs life span and ameliorates neurologic manifestations in Sandhoff disease mice. J. Clin. Invest. *101*, 1881–1888.

O'Leary, S.M., Coleman, M.M., Chew, W.M., Morrow, C., McLaughlin, A.M., Gleeson, L.E., O'Sullivan, M.P., and Keane, J. (2014). Cigarette smoking impairs human pulmonary immunity to Mycobacterium tuberculosis. Am. J. Respir. Crit. Care Med. *190*, 1430–1436.

Pagán, A.J., Yang, C.T., Cameron, J., Swaim, L.E., Ellett, F., Lieschke, G.J., and Ramakrishnan, L. (2015). Myeloid growth factors promote resistance to mycobacterial infection by curtailing granuloma necrosis through macrophage replenishment. Cell Host Microbe *18*, 15–26.

Peão, M.N., Aguas, A.P., de Sá, C.M., and Grande, N.R. (1993). Morphological evidence for migration of particle-laden macrophages through the interalveolar pores of Kohn in the murine lung. Acta Anat. (Basel) *147*, 227–232.

Peri, F., and Nüsslein-Volhard, C. (2008). Live imaging of neuronal degradation by microglia reveals a role for v0-ATPase a1 in phagosomal fusion in vivo. Cell *133*, 916–927.

Philips, J.A., and Ernst, J.D. (2012). Tuberculosis pathogenesis and immunity. Annu. Rev. Pathol. *7*, 353–384.

Platt, F.M., Boland, B., and van der Spoel, A.C. (2012). The cell biology of disease: lysosomal storage disorders: the cellular impact of lysosomal dysfunction. J. Cell Biol. *199*, 723–734.

Ratcliffe, H.L., and Wells, W.F. (1948). Tuberculosis of rabbits induced by droplet nuclei infection; initial response to infection. J. Exp. Med. *87*, 575–584.

Srivastava, S., Ernst, J.D., and Desvignes, L. (2014). Beyond macrophages: the diversity of mononuclear cells in tuberculosis. Immunol. Rev. *262*, 179–192.

Sumpter, C., and Chandramohan, D. (2013). Systematic review and meta-analysis of the associations between indoor air pollution and tuberculosis. Trop. Med. Int. Health *18*, 101–108.

Sweeney, S., Grandolfo, D., Ruenraroengsak, P., and Tetley, T.D. (2015). Functional consequences for primary human alveolar macrophages following treatment with long, but not short, multiwalled carbon nanotubes. Int. J. Nanomedicine *10*, 3115–3129.

Takaki, K., Davis, J.M., Winglee, K., and Ramakrishnan, L. (2013). Evaluation of the pathogenesis and treatment of Mycobacterium marinum infection in zebrafish. Nat. Protoc. *8*, 1114–1124.

Tobin, D.M., Vary, J.C., Jr., Ray, J.P., Walsh, G.S., Dunstan, S.J., Bang, N.D., Hagge, D.A., Khadge, S., King, M.-C., Hawn, T.R., et al. (2010). The lta4h locus modulates susceptibility to mycobacterial infection in zebrafish and humans. Cell *140*, 717–730.

Traynor, D., and Kay, R.R. (2007). Possible roles of the endocytic cycle in cell motility. J. Cell Sci. *120*, 2318–2327.

Urdahl, K.B. (2014). Understanding and overcoming the barriers to T cell-mediated immunity against tuberculosis. Semin. Immunol. *26*, 578–587.

Varshney, G.K., Lu, J., Gildea, D.E., Huang, H., Pei, W., Yang, Z., Huang, S.C., Schoenfeld, D., Pho, N.H., Casero, D., et al. (2013). A large-scale zebrafish gene knockout resource for the genome-wide study of gene function. Genome Res. *23*, 727–735.

Veale, K.J., Offenhäuser, C., Whittaker, S.P., Estrella, R.P., and Murray, R.Z. (2010). Recycling endosome membrane incorporation into the leading edge regulates lamellipodia formation and macrophage migration. Traffic *11*, 1370–1379.

Verrall, A.J., Netea, M.G., Alisjahbana, B., Hill, P.C., and van Crevel, R. (2014). Early clearance of Mycobacterium tuberculosis: a new frontier in prevention. Immunology *141*, 506–513.

Zimran, A. (2011). How I treat Gaucher disease. Blood *118*, 1463–1471.

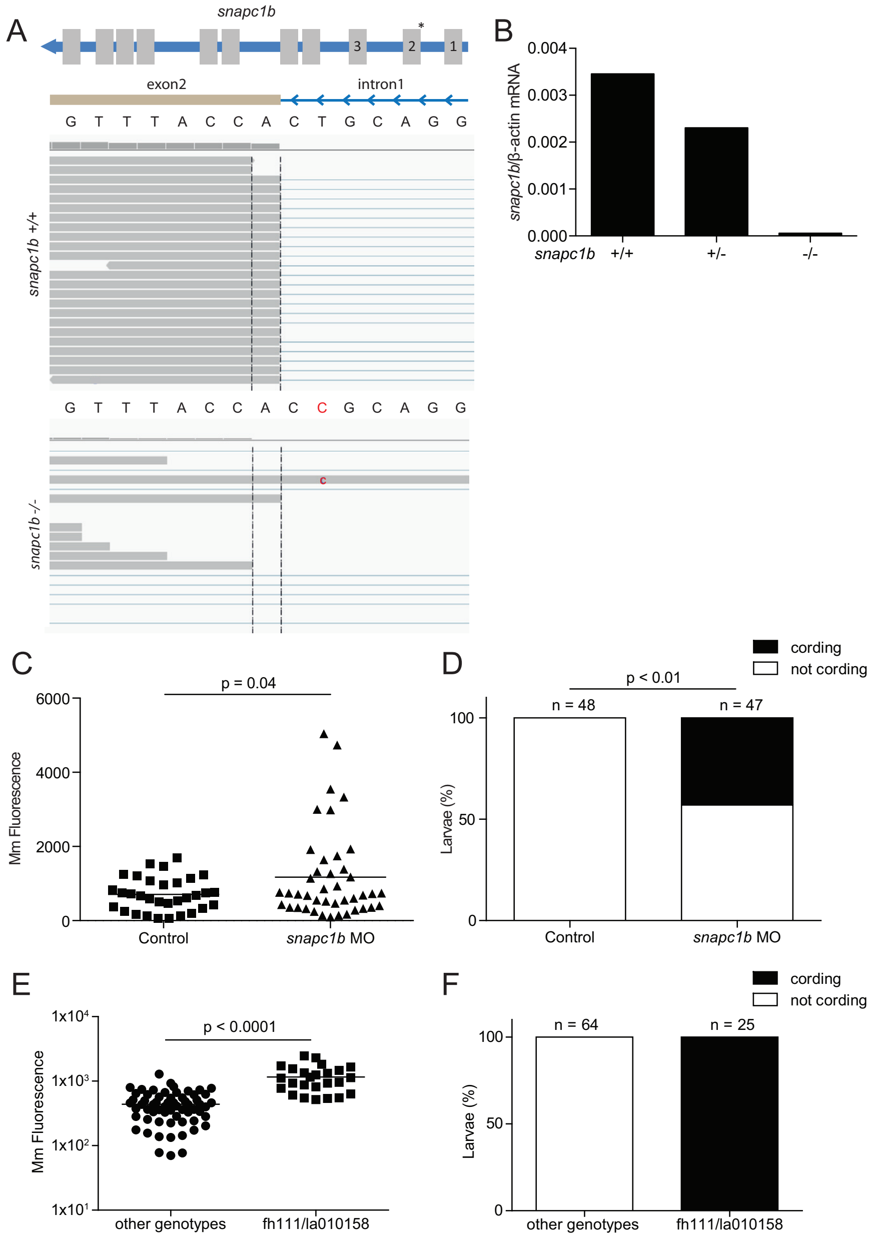

Figure S1. Genetic Disruption of the *snapc1b* Locus Confers Susceptibility to *M. marinum* Infection, Related to Figure 1

(A) Top: Diagram of *snapc1b* gene showing introns (blue), exons (gray), and location of the *fh111* splice acceptor mutation denoted by an asterisk above the relevant exon-intron boundary. Bottom: RNA-sequencing reads aligned to the exon 2 splice acceptor site from WT and *snapc1b*$^{fh111/fh111}$ mutant larvae with wild-type and mutant sequence. The *snapc1b(fh111)* mutation is denoted in red.

(B) Quantitative real-time PCR of properly spliced *snapc1b* transcript in *snapc1b*$^{+/-}$ incross larvae at 6 dpf. Values normalized to transcript level of β-*actin*, representative of two experiments.

(C and D) Quantification of bacterial burden (C) and cording (D) in control and morphant larvae at 4 dpi with 250 Mm.

(E and F) Quantification of bacterial burden and cording in *snapc1b*$^{Tg(la010158)/+}$ × *snapc1b*$^{fh111/+}$ cross larvae at 5 dpi with 150 Mm.

Statistical significance was assessed by Student's t test (C,E) and Fisher's exact test (D).

This is an open access article under the CC BY license (http://creativecommons.org/licenses/by/4.0/).

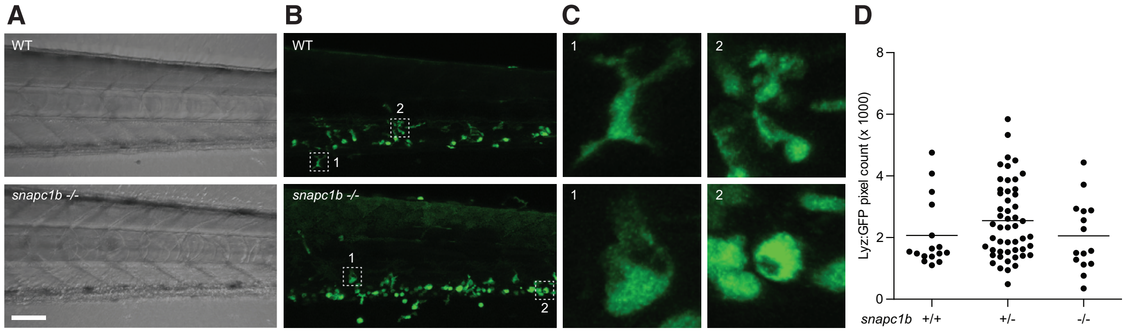

Figure S2. *snapc1b* Mutants Have Numerous Vacuolated Macrophages and Normal Neutrophil Numbers in the Caudal Hematopoietic Tissue, Related to Figure 1

(A and B) (A) Brightfield and (B) confocal images of the CHT of representative WT and snapc1b-/- mutant larvae at 5 dpf. Scale bar 50μm.

(C) 8X magnification of outlined regions in (B) showing normal (top) and vacuolated (bottom) morphology.

(D) Quantification of Lyz:eGFP positive, green fluorescent neutrophils in *snapc1b*$^{+/-}$ incross larvae at 6 dpf.

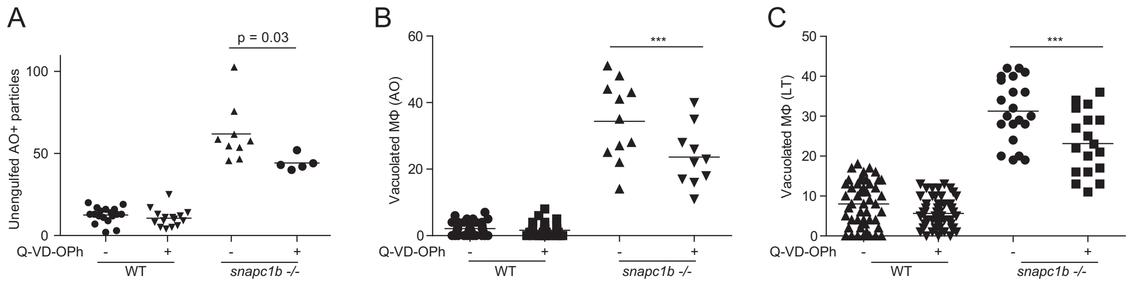

Figure S3. Global Inhibition of Apoptosis Reduces the Abundance of Vacuolated Macrophages in the *snapc1b* Mutant, Related to Figure 2

(A) Quantification of extracellular AO positive particles in WT and *snapc1b* mutant larvae at 3 dpf following treatment with 10 μM Q-VD-OPh or DMSO control.

(B) Quantification of AO-positive vacuolated macrophages in *snapc1b* mutant larvae and WT siblings at 3dpf following treatment with 10 μM Q-VD-OPh or DMSO control.

(C) Quantification of LysoTracker-positive macrophages in *snapc1b* mutant larvae and WT siblings at 3dpf following treatment with 50 μM Q-VD-OPh or DMSO.

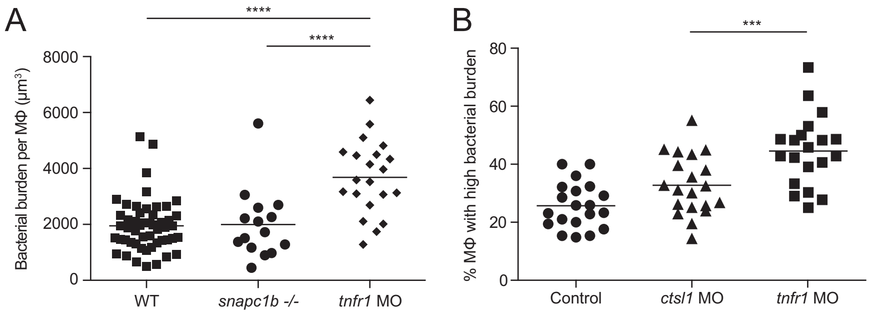

Figure S4. Macrophages of *snapc1b* Mutant and cathepsin L-Deficient Animals Restrict Mycobacterial Growth Normally, Related to Figure 4

(A) Macrophage intracellular bacterial burdens of *snapc1b*$^{+/-}$ incross larvae and *tnfr1* morphants infected with 100 red fluorescent Mm at 2 dpf in the caudal vein. Bacterial volume (μm^3) was quantified per animal from 3D confocal images captured in the tail region at 40 hpi. The intramacrophage replication of Mm is unrestricted in *tnfr1* morphants as expected (Clay et al., 2008; Pagán et al., 2015; Tobin et al., 2010).

(B) Percentage of macrophages with high intracellular bacterial burdens in control, *ctsl1* and *tnfr1* morphants infected with ~75 red fluorescent Mm at 2 dpf in the caudal vein. Bacterial burden was quantified per animal by counting the average number of bacteria per macrophage and categorizing as low (1-5 bacteria) or high (> 5 bacteria). Statistical significance was assessed by one-way ANOVA with Sidak's post test (A, B).

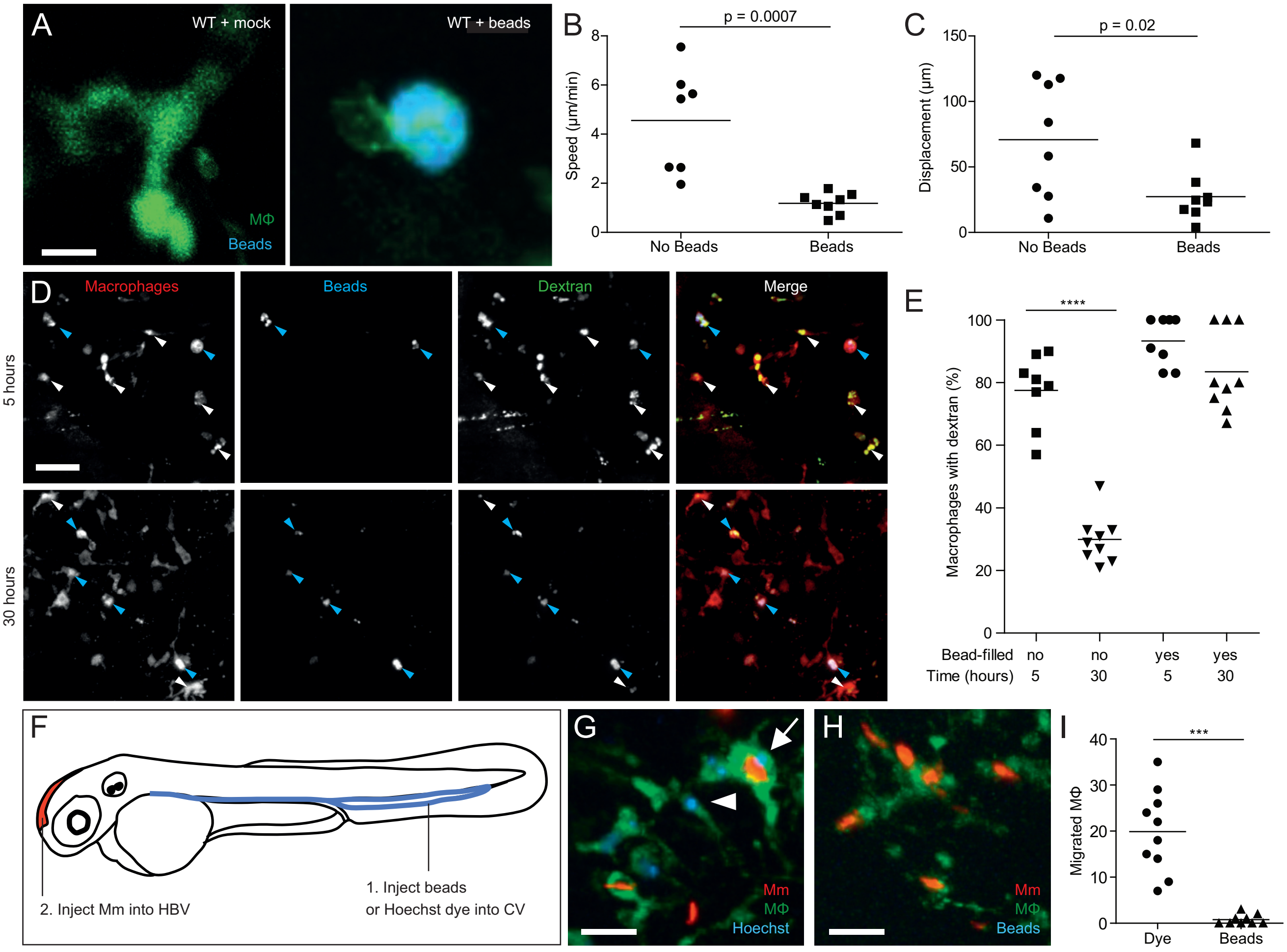

Figure S5. Lysosomal Accumulation of Inert Particles Compromises Endocytic Recycling and Migration to the Initial Site of Mycobacterial Infection, Related to Figure 6

(A) Confocal images of green fluorescent macrophages in larvae mock-injected or injected with $5x10^5$ blue fluorescent 1 μm polystyrene beads. Scale bar, 12 μm.

(B and C) Speed (B) and displacement (C) of macrophages with and without beads.

(D) Confocal images of red fluorescent macrophages in 3dpf larvae pre-loaded with blue fluorescent polystyrene beads as in (A), injected 12 hr later with green fluorescent dextran and imaged at 5 and 30 hr post-dextran injection. Blue and white arrowheads denote macrophages containing dextran, with and without blue beads, respectively. Scale bar, 50 μm.

(E) Quantification of macrophages that retained dextran at 5 and 30 hr post injection.

(F) Diagram showing the experimental outline in which 2 dpf larvae were injected with Hoechst dye or beads in the CV followed by infection in the HBV with 200 Mm.

(G and H) Confocal images of larval HBV containing green-fluorescent macrophages following CV injections with Hoechst (G) or blue fluorescent beads (H). Arrow and arrowhead denote Hoechst-positive macrophages that have migrated from the CHT, with and without phagocytosed red fluorescent Mm, respectively. Scale bar, 10 μm.

(I) Number of macrophages in the HBV after injection of dye or beads in the CV followed by Mm infection in the HBV.

Statistical significance was assessed using Student's t test (B, C, and I), and one-way ANOVA with Sidak's post test (E).

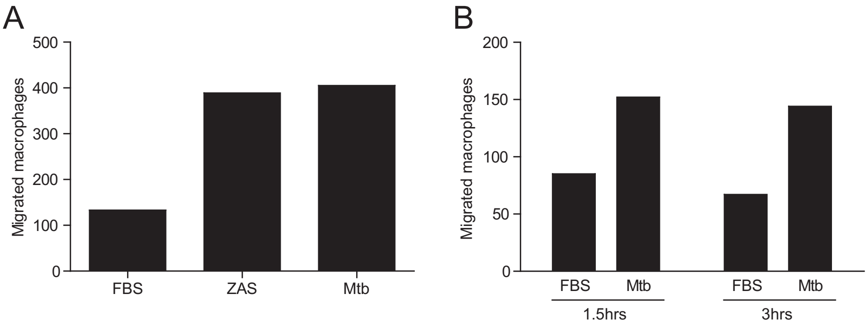

Figure S6. Alveolar Macrophage Migration to ZAS and Mtb, Related to Figure 7
(A) Migration of macrophages from nonsmoker SJH209 to 0.1% FBS, ZAS or Mtb at 2 hr.
(B) Migration of macrophages from ex-smoker to Mtb at 1.5 and 3 hr in transwell assay.

Cell, Volume *165*

Supplemental Information

Lysosomal Disorders Drive Susceptibility to Tuberculosis by Compromising Macrophage Migration

Russell D. Berg, Steven Levitte, Mary P. O'Sullivan, Seónadh M. O'Leary, C.J. Cambier, James Cameron, Kevin K. Takaki, Cecilia B. Moens, David M. Tobin, Joseph Keane, and Lalita Ramakrishnan

Supplemental Experimental Procedures

Bacterial culture

All Mm strains were grown under hygromycin (Mediatech) selection in Middlebrook's 7H9 medium (Difco) supplemented with glycerol, oleic acid, albumin, dextrose, and Tween-80 (Sigma) (Takaki et al., 2013). Single-cell stocks of Mm were prepared as previously described prior to injection (Takaki et al., 2013). Inocula were determined by microinjection onto 7H10 plates.

Zebrafish husbandry and larval injections

The Tg(*mpeg1:YFP)*^{w200}, Tg(*mpeg1:Brainbow)*^{w201} (expressing tdTomato) and Tg(*lysC:eGFP)*^{nz117} lines were used as previously described (Hall et al., 2007; Pagan et al., 2015; Roca and Ramakrishnan, 2013). All transgenic and *snapc1b(fh111)* lines were maintained as outcrosses to AB.

Bead injections

As a chemoattractant, heat killed bacteria were prepared by incubating a 2x10^4/uL culture of Mm in 7H9.OADS at 80°C for 30min. Sterile blue fluorescent 1 micron beads (Life Technologies F-8814) were mixed with heat killed Mm at a 1:9 ratio of beads:heat-killed Mm and then diluted to $3.64x10^3$ beads/nL. Approximately 5 nL of the resulting bead:heat-killed Mm mixture was injected into the hindbrain ventricle of 2 dpf larvae for a total of $1.8 x10^4$ beads per larva. Images were captured using confocal laser microscopy 24 hours following injection.

Dye and protein injections

LysoTracker Red DND-99 dye (DMSO solution, Life Technologies) was diluted 1:25 in PBS prior to injection of 5 nl into the HBV of 3 dpf larvae. MagicRed-Cathepsin (Immunochemistry Technologies, LLC) was resuspended at the concentration suggested by the manufacturer in DMSO, diluted 1:1 in 1xPBS and injected into the brain of 3 dpf larvae. Dextran-Alexa488 (10,000MW, Molecular Probes) was resuspended in 1xPBS to a concentration of 1 mg/mL, then diluted 1:100 prior to injection into the brain of 3 dpf larvae. CCL2 was injected into the HBV as previously described (Cambier et al., 2014).

Construction of Venus-V2A-*ctsl1* plasmid and *in vitro* transcription

The zebrafish *ctsl1* gene was amplified from cDNA and used to replace *csf1a* in a vector containing pCMV:nlsVenus-V2A (a gift from D. Parichy) by Gibson Assembly (Life Technologies). In vitro transcription was performed with mMessage mMachine SP6 kit (Life Technologies).

Morpholino and RNA injections

All morpholinos used in this work (Table S1) and *in vitro* transcribed *ctsl1* mRNA were diluted in a 1x Tango Buffer (Thermo Scientific), 2% phenol red sodium salt solution (Sigma) and injected 1 nL into the yolk of 1-2 cell stage embryos (Tobin et al., 2012).

Zebrafish mutagenesis, screening and positional cloning

fh111 carriers were identified by infection of gynodiploid larvae (Johnson et al., 1995) with 150-200 CFU of green fluorescent Mm. Putative *fh111* carriers were outcrossed to the wildtype WIK strain and mutants and carriers identified by random crosses between siblings. Bulk segregant analysis (BSA) was performed on pools of mutant progeny and phenotypically wildtype as described (Bahary et al., 2004). Intermediate and fine mapping were conducted using published markers and new markers generated by resequencing of mutant and wild-type progeny within the linked region. Fine mapping determined the causative locus lay between a WIK AseI site at 37.97 mb and a SNP at 38 mb on Chromosome 13. This region contained a single gene, *snapc1b*. Sequencing of all exons and splice junctions within the critical region in mutant embryos and their siblings identified a single splice acceptor site mutation immediately upstream of exon 2, in which the canonical A of the highly conserved AG immediately upstream of the splice acceptor site was converted to GG at position chr13:37996163 (A to G transition). We detected no recombination events at this locus in 854 total meioses from mapping crosses.

Staining

Neutral red staining of 6 dpf larvae was performed as described (Herbomel et al., 2001). Acridine Orange staining was performed by soaking larvae in fish water containing 2 μg/mL acridine orange (ImmunoChemistry, Bloomington, MN) for 30 minutes at 29°C in the dark. Larvae were washed for 5 minutes in fish water at room

temperature and then mounted for microscopy as previously described (Yang et al., 2012).TUNEL assay assay staining of larvae was performed by incubationfor 24 hr in 50 µM of Q-VD-OPh (R&D Systems, Inc.) dissolved in DMSO, after which TUNEL staining was performed as described (Volkman et al., 2004).

Drug treatments

E64d. E64d (Sigma) was dissolved in DMSO and added to fish water to a final concentration of 2-3 µg/mL in 0.5% DMSO. Larvae at 2 dpf were treated with the resulting E64d solution for 24 hours and then transferred to clean fish water for the duration of the experiment.

Q-VD-OPh: Larvae were treated with 50 µM Q-VD-OPh in 0.5% DMSO commencing at 2 dpf with daily water changes.

Genotyping

HRM genotyping: High resolution melt genotyping was conducted using the CFX Connect Real- Time PCR Detection System (BioRad) and the EvaGreen iTag Precision melt supermix. HRM primers were used at final concentration of 0.2 mM with ~10 ng of gDNA in 10 ul reaction. PCR was conducted using Biorad HRM standard cycling parameters.

snapc1b$^{\mathbf{Tg}(\boldsymbol{la010158})}$: The retroviral insertion mutant *snapc1b*$^{Tg(la010158)}$ (ZIRC) (GenBank ID JM495858.1) was genotyped using agarose gel electrophoresis using primers flanking the insertion site and a primer specific for the retroviral insertion (GT186). Wildtype Forward: 5' - GCTGAAATCCATGTCCTTCCA - 3'. GT186 Forward: 5' - GAGTGATTGACTACCCGTCAGCGG - 3'. Common Reverse: 5' - TGCTTTTCTCATGAGCTTCTCT - 3'

snapc1b$^{\boldsymbol{fh111}}$**:** *snapc1b*^{fh111} mutants were genotyped using High Resolution Melt (Tucker and Huynh, 2014). An insertion-deletion polymorphism is present in intron 1-2 in the wildtype AB population. The genomic sequences are

*

Reference 5'- ATTTTTTTCCCTG---C**A**GTGGTAAAC -3'

Fh111 5'- ATTTTTTTCCCTG---C**G**GTGGTAAAC -3'

WT INDEL 5'- ATT----------AAAC**A**GTGGTAAAC -3'

All genotypes are distinguishable with HRM sequencing primers, which bind outside of the polymorphic region: snapc1b_HRM Forward: 5' - CCACAAATTCCAAATGACATTGA -3'. snapc1b_HRM Reverse: 5' - CCTTTTCTCTCTTGGCTCTTGTTT -3'.

Quantitative RT-PCR

Quantitative RT-PCR was performed as previously described (Clay et al., 2007). Total RNA from batches of ~30 embryos per biological replicate was isolated with TRIzol Reagent (Life Technologies) and used to synthesize cDNA with Superscript II reverse transcriptase and oligo dT primers (Invitrogen). Quantitative RT-PCR assays were performed with SYBR green PCR Master Mix (Applied Biosystems) on an ABI Prism 7300 Real Time PCR System (Applied Biosystems). Each biological replicate was run in triplicate, and average values were plotted. Data were normalized to *b-actin* for ΔCt analysis. Primers used for RT-qPCR in this study were: Spliced *snapc1b* Forward: 5'- TGA AAC ACT GCT TGG TCG TC -3'; Spliced *snapc1b* Reverse: 5'- CCC GAC TCT GAT CTG GAA AG -3'; *ctsbb* Forward: 5'- GAG GAA CAG AGG AAC AGA CTT TA-3'; *ctsbb* Reverse: 5'- TGA TCA TCT CAT CAT GTG TGT GA-3'; *ctsl1* Forward: 5'- AGG AAG AGT CAC ACC GTC AG -3'; *ctsl1* Reverse: 5'- GTC ACA TAG CCC TTG TCC CT -3'*; b-actin* Forward 5'- ACC TGA CAG ACT ACC TGA TG -3'; *b-actin* Reverse 5'- TGA AGG TGG TCT CAT GGA TAC -3'; *tnf* Forward 5'- AGG CAA TTT CAC TTC CAA GG -3'; *tnf* Reverse 5'- CAA GCC ACC TGA AGA AAA GG -3'.

RNA-sequencing and analysis

Individual larvae were euthanized at 6 dpf and a small piece of tissue from the head of each animal removed with forceps. The head was placed in HotShot DNA extraction bufferand the torso was placed in 50 µL Trizol for RNA isolation (Invitrogen). gDNA was genotyped by HRM and mutant RNA pooled. Trizol extraction followed as per manufacturer's protocol. RNA was purified with LiCl extraction. Library construction was conducted by the Fred Hutchinson Cancer Research Center Genomics Core and proceeded as described (Miller et al., 2013). Approximately 1 µg RNA was poly-A selected, chemically fragmented and cDNA reverse transcribed using random hexamers. Libraries were prepared using the TruSeq Illumina protocol. Sequencing was performed on an Illumina HiSeq 2000 using 50 bp paired-end reads. RNA sequencing reads were aligned to the zebrafish reference genome

(Zv9.69) using the TopHat/Bowtie aligner (Trapnell et al., 2013). Transcriptome expression analysis was then conducted using the Cufflinks software pipeline (Trapnell et al., 2013). Validation of splicing perturbations in *snapc1b* mutants was assessed using IGV (Thorvaldsdóttir et al., 2013).

Microscopy

Zebrafish: Fluorescence microscopy was performed as previously described (Takaki et al., 2013; Yang et al., 2012). Quantification of bacterial burdens, enumeration of macrophage numbers, and assessments of mycobacterial cording were performed with a Nikon Eclipse Ti-E inverted microscope fitted with 2x, 4x and 10x objectives. Cording was visually assessed as previously described (Clay et al., 2008). For confocal microscopy, larvae were anesthetized in fish water containing 0.025% Tricaine and embedded in 1.5% low melting point agarose on optical bottom plates (MatTek Corporation). A Nikon A1 confocal microscope with a 20x Plan Apo 0.75 NA objective was used to generate 40-120 μm z-stacks with 1–2 μm step size. The galvano scanner was used for all static imaging, and the resonant scanner was used for time-lapse imaging. Time-lapse images were taken at 3-15 minute intervals for 5-20 hours. Data were acquired with NIS Elements Version 4.3 (Nikon). Macrophage tracks were generated and volume rendering performed using Imaris 7.7-8.0 (Bitplane Scientific Software). Movies were exported from Imaris to iMovie (Apple Corporation) for addition of labels and stitching. All microscope scoring of staining, bacterial morphology and macrophage morphology was performed blinded.

Human: Adherence purified AM were fixed with 2% paraformaldehyde, stained with Hoechst 33342 (10 μg/ml) and analyzed using a Zeiss LSM 510 laser confocal microscope (Carl Zeiss Microscopy GmbH, Oberkochen, Germany) to detect autofluorescence. Phase contrast images were collected with a transmitted light detector. Images were generated and viewed using Zen software (Carl Zeiss Microscopy).

Human alveolar macrophage migration assay

Human alveolar macrophages: Non-smokers were defined as those subjects reporting no smoking history, ex-smokers were defined as those having ceased smoking more than 6 months prior to sampling and smokers were defined as those currently smoking. Bronchial washing fluid was filtered through a 100 μm nylon strainer (BD Falcon, BD Bioscience, Belgium) and centrifuged at 390 g for 10 min. AM were resuspended in RPMI

1640 culture media supplemented with 0.1% heat inactivated fetal bovine serum (FBS, Gibco), 50 U/ml amphotericin B and 50 µg/ml cefotaxime. For characterization of initial cell population, 0.5 x 10^5 cells were seeded on an 8 chamber Lab-teks® (Thermo Fisher Scientific), fixed with ice cold methanol containing 10 µg/ml of Hoechst 33258 (Sigma Aldrich) for 4 minutes.

Mycobacteria: *M. tuberculosis* H37Ra was obtained from the American Type Culture Collection (ATCC 25177, Manassas, VA) and prepared as previously described (Ryan et al, 2011) and resuspended at 1 x 10^8 CFU/ml in RPMI 1640 with 0.1% heat inactivated fetal bovine serum (RPMI/0.1% hiFBS).

Migration assay: The migration assay was carried out in transwell chambers consisting of 8 µm pore polycarbonate cell culture inserts in a 24-well companion plate (Corning Costar™, Nijmegen, Netherlands) (Opalek et al., 2007). Experiments were performed in triplicate unless otherwise stated. Mtb, Tuberculin PPD (30µg/ml) (Statens Serum Institut, Denmark) diluted in RPMI/0.1% hiFBS, or RPMI/0.1% hiFBS alone or RPMI/10% zymosan-activated serum (ZAS) were placed in the lower chambers. AM (0.5 x 10^5 cells) were added to the upper chambers and plates were incubated at 37°C, 5% CO_2 for 2 hrs unless otherwise stated. Migrated cells were fixed in ice-cold methanol containing 10 µg/ml of Hoechst 33258 (Sigma Aldrich) for 4 minutes. AM were counted by fluorescent microscopy (Olympus IX51, Olympus Europa GmbH, Germany) to establish the number of migrating cells and the percentage vacuolated AM in the initial and migrating cell populations.

Neutral red staining of lysosomes: Adherence-purified AM cultured in 24 well plates were stained by adding neutral red (Sigma) (40 µg/ml) and incubating for 30 min at 37°C, 5% CO_2 (Repetto et al., 2008). Images were obtained using an Olympus IX51 microscope with a DP71 CCD camera and cellSens software.

Table S1. Morpholinos used in this study, related to Experimental Procedures.

Gene[a]	Used name[b]	Morpholino sequence	MO target	MO concentration (mM)	Reference
snapc1b	snapc1b	CTTGTTTACCACTGCAGGGAAA[illegible]	i1/e2	0.05	This work
snapc1b	snapc1b	ACCACTTCCCTGAAATGCTCCA[illegible]	atg/5'UTR	0.5	This work
ctsl.1	ctsl1	GTTTGTTTTAATTTCCTACCAT[illegible]	e3i3	1.3	This work
ctsbb	ctsbb	ACACTGCAGATGGAGAAAACA[illegible]	i3e4	0.05	This work
ctsbb	ctsbb atg	GCGAACACACACACACGCCAC[illegible]	atg/5'UTR	0.05	This work
tnfr1	tnfr1	CTGCATTGTGACTTACTTATCG[illegible]	E5/i6	0.32	(Roca & Ramakrishnan, 2013)
hexa	hexa	ACATTGCCAGGAAGTACTCAC[illegible]	e2/i2	1	This work
gba	gba	CGTCTGATTATTGTTACACACC[illegible]	e1/i1	0.5	This work
arsa	arsa	TATGTTTGACCTGGCTTTACCT[illegible]	e1/i1	1	This work

[a]Gene abbreviations follow the "Zebrafish Nomenclature Guidelines" (https://wiki.zfin.org/display/general/ZFIN+Zebrafish+Nomenclature+Guidelines)

[b]Name, often simplified, that was used in this paper

Table S2. Selected differentially expressed genes in *snapc1b* mutants, related to Figure 1.

Category	Gene	*snapc1b* +/+ FPKM	*snapc1b* -/- FPKM	Fold change (mutant/wt)	Associated LSD
Myelopoeisis	csf1rb	1.07	0.32	0.29	
	csf3r	0.85	0.71	0.84	
	csf1a	1.78	1.85	1.04	
	csf1ra	5.24	5.86	1.12	
	csf2rb	0.34	0.46	1.37	
	csf1b	2.60	3.75	1.44	
	spi1b	2.57	2.47	0.96	
	spi1a	1.63	2.46	1.51	
	irf8	0.89	1.99	2.23	
	mpeg1 (2 of 3)	11.05	32.07	2.90	
	mpeg1	3.12	4.82	1.54	
	gata1a	1.78	1.24	0.69	
	gata1b	0.09	0.03	0.35	
	gata2a	4.31	3.49	0.81	
	gata2b	0.49	0.40	0.80	
	gata3	20.87	15.79	0.76	
	gata4	3.26	2.70	0.83	
	gata5	5.73	7.57	1.32	
	gata6	9.49	10.00	1.05	
	cebp1	0.41	0.29	0.71	
	cebpa	16.82	17.12	1.02	
	cebpb	24.19	44.02	1.82	
	cebpd	116.14	137.37	1.18	
	cebpg	31.68	29.80	0.94	
Inflammation	atf3	8.93	15.99	1.79	
	cebpb	24.19	44.02	1.82	
	elf3	15.67	28.85	1.84	
	junbb	16.86	38.72	2.30	
	junba	11.23	36.13	3.22	
	ptgs2a	6.72	10.64	1.58	
	rgs4	22.31	45.92	2.06	
	ptgs2b	2.62	5.71	2.18	
	ncf1	2.48	5.44	2.20	
	plek	1.23	118.44	96.27	
	m17	0.04	0.21	5.10	
	il1b	0.89	9.07	10.24	
	mmp13a	2.23	10.09	4.52	
	mmp9	3.89	23.70	6.09	

	irg1	0.46	3.62	7.93	
	irg1l	9.42	91.73	9.74	
	tnfa	0.034	0.18	2.44	
Lysosomal	npc1	15.07	20.63	1.37	
	npc2	59.17	63.20	1.07	
	smpd1	7.82	9.84	1.26	
	hexb	26.29	25.22	0.96	
	hexa	8.24	6.58	0.80	Tay-Sachs
	arsa	3.48	2.59	0.75	Metachromatic leukodystrophy
	arsb	6.71	7.20	1.07	
	gba	4.82	5.00	1.04	Gaucher's
	gla	0.52	0.42	0.80	
	ctsa	23.99	24.66	1.03	
	ctsba	72.88	96.18	1.32	
	**ctsbb**	**57.38**	**5.18**	**0.09**	
	ctsc	25.25	24.17	0.96	
	ctsd	98.61	134.24	1.36	
	ctsf	66.41	60.22	0.91	
	ctsh	44.06	22.82	0.52	
	ctsk	14.80	19.61	1.33	
	ctskl	5.20	3.85	0.74	
	**ctsl.1**	**378.02**	**47.93**	**0.13**	
	ctsl1a	305.85	655.09	2.14	
	galns	2.57	2.56	0.99	
	gnsa	10.79	10.74	1.00	
	gnsb	13.39	10.69	0.80	
	naglu	1.03	1.16	1.13	
	neu1	15.07	20.63	1.37	
	psap	59.17	63.20	1.07	
	scpep1	7.82	9.84	1.26	
	sgsh	26.29	25.22	0.96	
	idua	8.24	6.58	0.80	
	tpp1	3.48	2.59	0.75	
	tmem55a	6.71	7.20	1.07	
	tmem55b(1 of 2)	4.82	5.00	1.04	
	tmem55b (2 of 2)	0.52	0.42	0.80	
	lamp1	23.99	24.66	1.03	
	lamp2	71.91	86.35	1.20	
	atp6v1h	29.75	30.88	1.04	
	clcn7	5.19	6.04	1.16	
	**FPKM = Fragments per kilobase mapped**				

Table S4. Characteristics and migration of alveolar macrophages retrieved by bronchoalveolar lavage from non-smokers, ex-smokers and smokers, related to Figure 7.

				Initial AM population		Migrated AM to Mtb H37Ra	
**Sample**	**Age years (mean ± SEM)**	**Smoking status**	**Pack years (years ceased)**	**# cells counted**	**% vacuolar cells (mean ± SEM)**	**# cells counted**	**% vacuolar cells (mean ± SEM)**
SJH209		non-smoker	-	320	2.81	405[a]	non-detectable
SJH222		non-smoker	-	441	1.36	-	-
SJH223		non-smoker	-	580	0.00	-	-
SJH226		non-smoker	-	479	0.42	-	-
	**52.75 ± 4.27**				**1.15 ± 0.62**		
SJH210		ex-smoker	40 (6)	107	9.35	288[ab] (284[c])	non-detectable
SJH211		ex-smoker	27 (16)	201	4.48	152[ab] (144[c])	non-detectable
SJH212		ex-smoker	(0.5)	418	8.61	338	non-detectable
SJH219		ex-smoker	54 (34)	439	9.57	-	-
	**55.25 ± 9.74**				**8.00 ± 1.19**		
SJH214		smoker	56	441	52.15	203	2.96
SJH215		smoker	100	398	60.80	40	30.00
SJH216		smoker	20	429	74.36	41	21.95
SJH220		smoker	5.7	412	69.66	250	19.20
SJH227		smoker	52.5	424	81.37	68	32.35
SJH218		smoker	47	531	68.17	-	-
	**65 ± 3.04**				**67.75 ± 4.18**		**21.29 ± 5.19**

[a] Experiments not performed in triplicate [b] migrated cells at 1.5hr timepoint [c] migrated cells at 3hr timepoint

Supplemental References

Bahary, N., Davidson, A., Ransom, D., Shepard, J., Stern, H., Trede, N., Zhou, Y., Barut, B., and Zon, L.I. (2004). The Zon laboratory guide to positional cloning in zebrafish. Methods Cell Biol *77*, 305-329.

Cambier, C.J., Takaki, K.K., Larson, R.P., Hernandez, R.E., Tobin, D.M., Urdahl, K.B., Cosma, C.L., and Ramakrishnan, L. (2014). Mycobacteria manipulate macrophage recruitment through coordinated use of membrane lipids. Nature *505*, 218-222.

Clay, H., Davis, J.M., Beery, D., Huttenlocher, A., Lyons, S.E., and Ramakrishnan, L. (2007). Dichotomous role of the macrophage in early Mycobacterium marinum infection of the zebrafish. Cell Host Microbe *2*, 29-39.

Clay, H., Volkman, H.E., and Ramakrishnan, L. (2008). Tumor necrosis factor signaling mediates resistance to mycobacteria by inhibiting bacterial growth and macrophage death. Immunity *29*, 283-294.

Cosma, C.L., Swaim, L.E., Volkman, H., Ramakrishnan, L., and Davis, J.M. (2006). Zebrafish and frog models of Mycobacterium marinum infection. Curr Protoc Microbiol *Chapter 10*, Unit 10B.12.

Hall, C., Flores, M.V., Storm, T., Crosier, K., and Crosier, P. (2007). The zebrafish lysozyme C promoter drives myeloid-specific expression in transgenic fish. BMC Dev Biol *7*, 42.

Herbomel, P., Thisse, B., and Thisse, C. (2001). Zebrafish Early Macrophages Colonize Cephalic Mesenchyme and Developing Brain, Retina, and Epidermis through a M-CSF Receptor-Dependent Invasive Process. Developmental Biology *238*, 274-288.

Johnson, S.L., Africa, D., Horne, S., and Postlethwait, J.H. (1995). Half-tetrad analysis in zebrafish: mapping the ros mutation and the centromere of linkage group I. Genetics *139*, 1727-1735.

Miller, A.C., Obholzer, N.D., Shah, A.N., Megason, S.G., and Moens, C.B. (2013). RNA-seq-based mapping and candidate identification of mutations from forward genetic screens. Genome Res *23*, 679-686.

Opalek, J.M., Ali, N.A., Lobb, J.M., Hunter, M.G., and Marsh, C.B. (2007). Alveolar macrophages lack CCR2 expression and do not migrate to CCL2. Journal of inflammation *4*, 19.

Pagan, A.J., Yang, C.T., Cameron, J., Swaim, L.E., Ellett, F., Lieschke, G.J., and Ramakrishnan, L. (2015). Myeloid Growth Factors Promote Resistance to Mycobacterial Infection by Curtailing Granuloma Necrosis through Macrophage Replenishment. Cell Host Microbe *18*, 15-26.

Repetto, G., del Peso, A., and Zurita, J.L. (2008). Neutral red uptake assay for the estimation of cell viability/cytotoxicity. Nat Protoc *3*, 1125-1131.

Roca, F.J., and Ramakrishnan, L. (2013). TNF dually mediates resistance and susceptibility to mycobacteria via mitochondrial reactive oxygen species. Cell *153*, 521-534.

Takaki, K., Davis, J.M., Winglee, K., and Ramakrishnan, L. (2013). Evaluation of the pathogenesis and treatment of Mycobacterium marinum infection in zebrafish. Nat Protoc *8*, 1114-1124.

Thorvaldsdóttir, H., Robinson, J.T., and Mesirov, J.P. (2013). Integrative Genomics Viewer (IGV): high-performance genomics data visualization and exploration. Brief Bioinform *14*, 178-192.

Tobin, D., Roca, F., Oh, S., McFarland, R., Vickery, T., Ray, J., Ko, D., Zou, Y., Bang, N., CHau, T., *et al.* (2012). Host genotype-specific therapies can optimize the inflammatory response to mycobacterial infections. Cell *148*, 434-446.

Trapnell, C., Hendrickson, D.G., Sauvageau, M., Goff, L., Rinn, J.L., and Pachter, L. (2013). Differential analysis of gene regulation at transcript resolution with RNA-seq. Nat Biotechnol *31*, 46-53.

Tucker, E.J., and Huynh, B.L. (2014). Genotyping by high-resolution melting analysis. Methods Mol Biol *1145*, 59-66.

Volkman, H.E., Clay, H., Beery, D., Chang, J.C., Sherman, D.R., and Ramakrishnan, L. (2004). Tuberculous granuloma formation is enhanced by a mycobacterium virulence determinant. PLoS Biol *2*, e367.

Yang, C.T., Cambier, C.J., Davis, J.M., Hall, C.J., Crosier, P.S., and Ramakrishnan, L. (2012). Neutrophils exert protection in the early tuberculous granuloma by oxidative killing of mycobacteria phagocytosed from infected macrophages. Cell Host Microbe *12*, 301-312.
